# Supplementary material for: Preferences of women in difficult life situations for a physical activity programme: protocol of a discrete choice experiment in the German NU-BIG project
Source: BMJ Open. 2023 Jul 17;13(7):e067235. doi: 10.1136/bmjopen-2022-067235 (PMC10357700; doi:10.1136/bmjopen-2022-067235)
Supplement: Supplementary data [file bmjopen-2022-067235supp001.pdf]

Preferences of women in difficult life situations for a physical activity program: protocol of a discrete choice experiment in the NU-BIG project

Sara Pedron, Annika Herbert-Maul, Alexandra, Stephanie Linder, Raluca Sommer, Markus Vomhof, Veronika Gontscharuk, Karim Abu-Omar, Ansgar, Heiko Ziemainz, Rolf Holle & Michael Laxy

Supplementary material

Appendix 1: course description in each BIG-site

Table A.1: description of courses in active sites in 2019

	time unit	Number of participants	Number of fitness courses	Number of water courses	Additional offers	Additional social activities	Planning & participation	mean number of participants per course
Erlangen	year	695	57	13	women-only bathing time, additional sport activities (dancing 2x per year, archery, climbing,…)	women´s breakfast (8x year), healthy diet course (2x year), arabic cuisine tasting (1x year)	Planning meeting (2x year)	10
Regensburg	I	166	34	3	women-only bathing time, biking course			10
	II	132						
	III	88						
Bayreuth	I	127	18	8	biking course			13
	II	112						
	III	108						
Straubing		144	10	0	boxing	integration course		14
Berlin (Treptow-Köpenick)		4	1					4
Nürnberg	I	63	0	3				21
	II	61	0	3				
	III	81	0	4				
Neustadt a.d. Aisch		20		2				10

Note: the table shows the number of participants in each active site in 2019, either for the whole years or for different course seasons (I, II or III).

Appendix 2: Systematic literature search protocol and results

Goal: finding studies which evaluated individual preferences for lifestyle programs/physical activity programs

Search in PubMed:

(physical activity[Title/Abstract]) AND (discrete choice[Title/Abstract])

((physical activity[Title/Abstract]) AND (conjoint analysis[Title/Abstract])

(lifestyle[Title/Abstract]) AND (discrete choice[Title/Abstract])

(coaching[Title/Abstract]) AND (discrete choice[Title/Abstract])

(coaching[Title/Abstract]) AND (conjoint analysis[Title/Abstract])

(sport[Title/Abstract]) AND (discrete choice[Title/Abstract])

(sport[Title/Abstract]) AND (conjoint analysis[Title/Abstract])

(empowerment[Title/Abstract]) AND (discrete choice[Title/Abstract])

(empowerment[Title/Abstract]) AND (conjoint analysis[Title/Abstract])

(exercise[Title/Abstract]) AND (discrete choice[Title/Abstract])

(exercise[Title/Abstract]) AND (conjoint analysis[Title/Abstract])

The two search phrases in gray did not lead to any result.

Flowchart of the retrieved studies and screening process

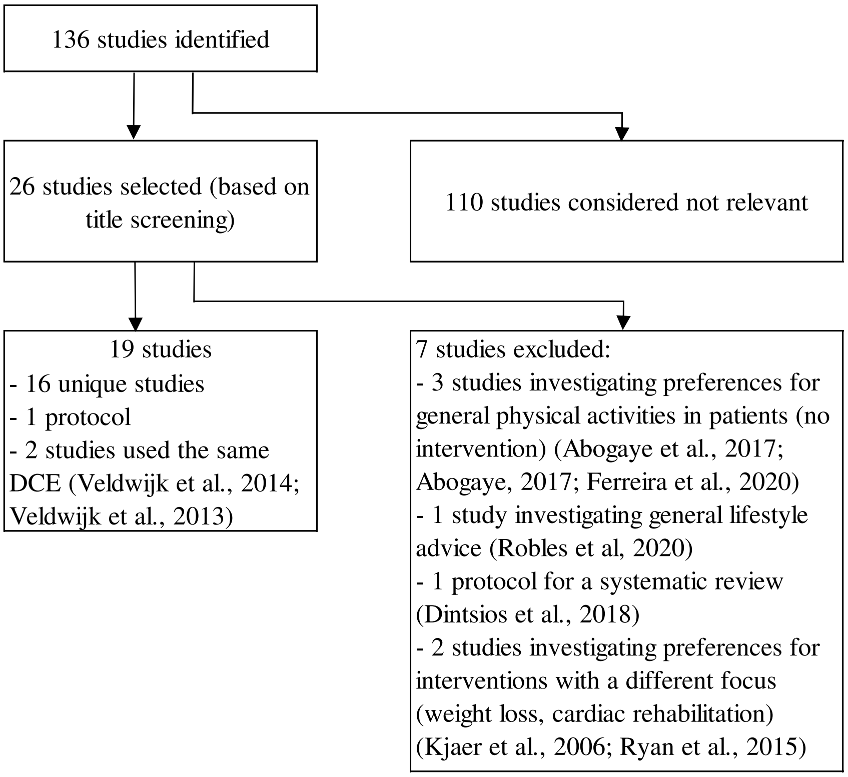

General overview of the retrieved studies

The search led to 18 studies and one protocol of one of the studies (Pinto et al., 2017). Two studies (Veldwijk et al., 2013; Veldwijk et al., 2014) used the same DCE, so we will refer to only one of them for simplicity. The studies focused on different topics but were all concerned in deploying DCE techniques to understand preferences for a physical activity intervention (sometimes labeled as "lifestyle intervention", including also diet). They dealt with the following topics:

- Lifestyle intervention for:
    - Diabetes prevention (Owen et al., 2010)
    - Stroke survivors (Geidl et al., 2018)
    - Knee osteoarthritis (Pinto et al., 2017; Pinto et al., 2019) [Study + Protocol]
    - Parkinson´s disease (Paul et al., 2021)
    - Diabetes patients (Salampessy et al., 2015; Sommer et al., 2020; Veldwijk et al., 2014, Veldwijk et al., 2013, Ramirez & Beale, 2016; van Gils et al., 2011) [two studies by Veldwijk using the same DCE]
    - Older adults (Brown, 2009)
- Focus on outcomes of health promotion interventions (Alayli-Goebbels, 2013)
- Focus on goals of health promotion interventions (Benning, 2020)
- Focus on financial incentives for physical activity (Farooqui, 2014; Giles, 2016; Molema, 2019, Wanders, 2014, Matsushita, 2017)

Systematic extraction of attributes and levels

We collected all attributes and levels from the selected studies. We eliminated those who were specific of the context analyzed and not transferrable to our context (diet related items, items related to a specific disease). Furthermore, we excluded those that were potentially relevant for a general physical activity intervention but were not applicable to our context (mixed groups including men, exercising alone/at home, schedule and goal setting by coach/physician, monetary interventions, goal setting, consultations with coach or physician, etc.). The remaining attributes are presented in Table A.2.

Table A.2: attributes and levels retrieved via systematic literature search

Attribute	Levels	Study
**Exercise frequency and duration**		
Exercise structure	Daily 30 min<br>daily 45 min<br>weekly 3h<br>weekly 5h	Owen et al., 2020
Frequency	1-2 sessions per week<br>3 sessions per week<br>4-5 sessions per week	Geidl et al., 2018
Duration	20-30 minutes per session<br>45-60 minutes per session	Geidl et al., 2018
Number of 45-minute exercise sessions per week	1x45-minute session per week<br>2x45-minute sessions per week<br>3x45-minute sessions per week<br>4x45-minute sessions per week	Paul et al., 2021
Total time required	12 hours/week<br>10 hours/week<br>8 hours/week<br>6 hours/week	Sommer et al., 2020
Time spent on the program	2.5 hours per week<br>4 hours per week	van Gils et al., 2011
Number of walking days per week for 3 months	2 days<br>3 days<br>5 days	Brown et al., 2009
walking time perday	20 min<br>30 min<br>45 min<br>75 min	Brown et al., 2009
time per week	0 h<br>2 h<br>4 h<br>6 h	Alayli-Goebbels et al., 201
Exercise per week	2 h<br>4 h<br>6 h	Benning et al., 2020
Average number of sessions required per week	1 session<br>2 sessions	Farooqui et al., 2014
Time per physical activity occasion	Low<br>Medium<br>High	Pinto et al., 2019

Attribute	Levels	Study
**Context**		
Group activities	No group activities<br>Group activities	Sommer et al., 2020
**Type of exercise**		
Type of exercise	Endurance<br>Muscular strength<br>Neuromuscular and flexibility<br>Mixed program	Geidl et al., 2018
Intensity	Light activity<br>Moderate activity<br>Vigorous activity	Geidl et al., 2018
Physical activity effort	Low<br>Medium<br>High	Pinto et al., 2019
Sports activity	Walking/cycling<br>Fitness (treadmill, rowing machine, bicycle)	van Gils et al., 2011
Exercise type	Dance program (eg, tango or waltz/foxtrot or Irish set dancing)<br>Balance exercise program (exercises to challenge balance, performed while standing, may include Tai-chi)<br>Muscle strength exercise program (exercises aimed at increasing muscle strength, eg, resistance exercises using free weights, exercises using your body weight, weight machines, weighted vests, TheraBand)<br>Aerobic exercise program (exercises to challenge the heart and lungs and make you puff, eg, fast walking, jogging, running, cycling)<br>Walking exercise program (eg, walking indoors and/or outdoors, treadmill walking, walking using visual and/or auditory cues)<br>Multimodal exercise program (including at least 2 of the following exercises): balance exercises, strength exercises, aerobic exercises, walking exercises	Paul et al., 2021
**Outcomes & goals**		
Outcomes	0.25 kg, no body change<br>0.5kg no body change<br>0.5kg body change<br>0.25 kg, body change	Owen et al., 2020
Expected outcome	No weight loss but feeling fitter 0<br>Weight loss of 5 kilograms and feeling fitter 5<br>Weight loss of 10 kilograms and feeling fitter 10	Wanders et al., 2014

Attribute	Levels	Study
Expected outcomes	No weights loss (reference) but you feel fitter<br>A weight loss of 5 kg and you feel fitter<br>A weight loss of 10 kg and you feel fitter	Salampessy et al., 2015
Expected outcomes	No weight loss but feeling more healthy<br>5 kilograms of weight lost and feeling more healthy<br>10 kilograms of weight lost and feeling more healthy	Veldwijk et al., 2013
expected weight loss	5 kg<br>10 kg	Benning et al., 2020
Goal	Looking better by losing weight<br>Increasing life expectancy by losing weight	Benning et al., 2020
clothing size above ideal	0 sizes<br>1/2 size<br>1 size<br>2 sizes	Alayli-Goebbels et al., 2013
Health benefits	Low<br>Medium<br>High	Pinto et al., 2019
Effect on your overall feeling of wellbeing	You will experience no improvement in overall feeling of wellbeing<br>You will experience small improvement in overall feeling of wellbeing<br>You will experience moderate improvement in overall feeling of wellbeing<br>You will experience large improvement in overall feeling of wellbeing	Paul et al., 2021
Future health state value	0.5<br>0.7<br>0.8<br>1	Alayli-Goebbels et al., 2013
start point of future health state	in 2 yrs<br>in 5 yrs<br>in 10 yrs<br>in 20 yrs	Alayli-Goebbels et al., 2013
life expectancy	80 yrs<br>81 yrs<br>82 yrs<br>83 yrs	Alayli-Goebbels et al., 2013
days without sufficient relaxation	0-1 per week<br>2-3 per week<br>4-5 per week<br>6-7 per week	Alayli-Goebbels et al., 2013

Attribute	Levels	Study
endurance	poor<br>modest<br>good<br>vey good	Alayli-Goebbels et al., 2013
experienced control over lifestyle choices	little<br>some<br>moderate<br>much	Alayli-Goebbels et al., 2013
Enjoyment	Low<br>Medium<br>High	Pinto et al., 2019
lifestyle improvemens of potential partner/children	yes<br>no	Alayli-Goebbels et al., 2013
**Schedule**		
Convenience (how well activity fits into your schedule)	Low<br>Medium<br>High	Pinto et al., 2019
**Costs**		
Enrollment fee	Free<br>$20<br>$50	Farooqui et al., 2014
Additional cost of gym/healthier food options	no additional cost<br>AU$ 15/week	Owen et al., 2020
Monthly cost	Low<br>Medium<br>High	Pinto et al., 2019
Out of pocket cost (in AU$) including travel costs per session	Out of pocket cost = $0 per session<br>Out of pocket cost = $10 per session<br>Out of pocket cost = $25 per session<br>Out of pocket cost = $50 per session<br>Out of pocket cost = $100 per session<br>Out of pocket cost = $150 per session	Paul et al., 2021
OOP costs	OOP costs of €75 per 3–6 months<br>OOP costs of €150 per 3–6 months<br>OOP costs of €225 per 3–6 months	Salampessy et al., 2015
Out-of-pocket costs	75 euro per year<br>150 euro per year<br>225 euro per year	Veldwijk et al., 2013
money per month	0 €<br>25 €<br>50 €<br>100 €	Alayli-Goebbels et al., 2013

Attribute	Levels	Study
Cost to travel to sessions	3 sessions<br>Free<br>$2<br>$5	Farooqui et al., 2014
**Travel time & location**		
Exercise location	In the local neighbourhood (eg, local parks, streets or outdoor public spaces)<br>At a hospital or health centre/practice<br>At a community centre or facility (eg, gym, community hall)<br>At multiple locations including home<br>At multiple locations excluding home	Paul et al., 2021
Travel time per exercise session	Less than 5 minutes each way travel time<br>5 minutes each way travel time<br>10 minutes each way travel time<br>15 minutes each way travel time<br>30 minutes each way travel time<br>60 minutes or more each way travel time	Paul et al., 2021
Time to travel to sessions	15 minutes<br>25 minutes<br>30 minutes<br>45 minutes	Farooqui et al., 2014
**Support**		
Amount of supervision	All of the exercise is supervised<br>Some of the exercise is supervised<br>None of the exercise is supervised	Paul et al., 2021
**Other**		
other information provided	no other information<br>written leaflet on benefits of activity and ways to be more active<br>face-to-face discussions on benefits of activity and ways to be more active<br>written leaflet and face-to-face idscussion on benefits of activity and ways to be more active	Giles et al., 2016

Appendix 3: further definition of attributes and levels based on experts feedback

In the meeting with the expert, 10 attributes were presented:

- *Price per course unit*
- *Course times*
- *Travel time to hall/course*
- *Additional social activities*
- *Additional course content*
- *Participation in organization*
- *Course registration*
- *Group composition*
- *Presence of childcare*
- *Course duration (in minutes)*

The discussion led to a reformulation of two attributes and the identification of an additional one. The additional attribute identified was "flexible intensity of the course" (i.e. if the intensity of the exercises within a course could be flexibly adjusted or not). The two attributes that were reformulated were:

- "course registration" (i.e. if all costs for the whole course must be paid in advance or if each appointment can be paid separately) was substituted by the attribute "trial lecture" (i.e. if a trial lecture is offered or not).
- "group composition" (i.e. women from the same social background/migration background vs mixed group) was substituted by an attribute specifically targeting respect of religious standards (i.e. framework: the hall is visible from the outside or not).

Based on the insights gained during the consultation with the experts, we performed a selection of those attributes which respected the following characteristics:

- Relevant for the overall utility
- Influenceable
- Close to reality (in the BIG program)
- Substitutive relation
- No dominance

Based on the insights from the experts we considered *presence of childcare* and *presence of trial lessons* as dominant criteria for the decision. These attributes, together with the presence of a female coach were considered as fix characteristics for the DCE.

According to the experts, the attribute "*course duration (in minutes)*" was distributed between 60 and 90 minutes for almost all offered courses. Given the limited variation, we decided to discard this attribute and fix it at 60 Minutes along the previously mentioned course characteristics.

The attribute "*course registration*" (paying everything at the beginning or paying each lesson at a time) was perceived as not relevant by the experts. Therefore we decided to exclude this attribute altogether.

The attribute "*flexible intensity (yes or no)*" was also deemed unimportant for the present DCE, since basically in all courses offered the intensity can be flexibly adjusted. Women also have the possibility of visiting a trial lesson and receive individual advice regarding their fitness level. This information will be included also in the fix course components.

The attribute "*framework (hall is visible from the outside yes or no)*" was deemed as a dominant criterion for some women (especially Muslim women) and not relevant for some other women. In both cases the attribute should not be included in the DCE. We therefore decided to fix this attribute as well.

The remaining attributes were included in the DCE. These are:

- *Price per course unit*
- *Course times*
- *Travel time to hall/course*
- *Additional activities (social + content)*
- *Participation in organization*

The attributes "*additional social activities*" and "*additional course content*" were merged into one attribute for the following reasons:

- The attribute additional course content was not clearly formulated and led experts to different interpretations/questions of what it means (e.g. additional lectures or additional content in the same appointment as the sport course? Etc.)
- At the moment in most BIG courses additional social activities include also the informal exchange on other topics (healthy diet, mental health, integration, help with translations,…) so that the two additional activities (social and content) largely overlap at the moment

The final attributes and levels included are (original language):

Attribute	Levels
**Kurszeiten**	Morgens (8-10 Uhr)
	Vormittags (10-12 Uhr)
	Nachmittags (16-18 Uhr)
	Abends (18-20 Uhr)
**Dauer Geh-/Fahrweg bis zum BIG-Kurs**	Maximal 10 Minuten
	Maximal 20 Minuten
	Maximal 30 Minuten
**zusätzliche soziale Aktivitäten im Rahmen von BIG organisiert**	Keine zusätzliche Aktivität wird organisiert
	Zusätzliche soziale Aktivitäten werden organisiert
**Berücksichtigung von Interessen und Wünsche bei der weiteren Kursplanung**	Interessen/Wünsche werden nicht abgefragt/nicht berücksichtigt
	Interessen/Wünsche werden abgefragt/berücksichtigt
**Kurskosten pro Kurseinheit**	0 €
	1 €
	2 €
	3 €
	4 €
	5 €

Appendix 4: list of attributes and levels in the original language (German)

Attribute	Levels
**Kurszeiten**	Morgens (8-10 Uhr)
	Vormittags (10-12 Uhr)
	Nachmittags (16-18 Uhr)
	Abends (18-20 Uhr)
**Dauer Geh-/Fahrweg bis zum BIG-Kurs**	Maximal 10 Minuten
	Maximal 20 Minuten
	Maximal 30 Minuten
**Zusätzliche soziale Aktivitäten im Rahmen von BIG organisiert**	Keine zusätzliche Aktivität
	Zusätzliche soziale Aktivitäten
**Berücksichtigung von Interessen und Wünsche bei der weiteren Kursplanung**	Interessen/Wünsche werden nicht berücksichtigt
	Interesse/Wünsche werden berücksichtigt
**Kurskosten pro Kurseinheit**	2,0 €
	3,5 €
	5,0 €
	6,5 €
	8,0 €
	9,5 €

Appendix 5: final DCE questionnaire (original language)

NU-BIG
Nach - Untersuchung von Bewegung als Investition in Gesundheit

BIG-Projekt

Fragebogen

Liebe Teilnehmerin,
„BIG" steht für „Bewegung als Investition in Gesundheit". Bei BIG werden Sport- und Bewegungskurse für Frauen angeboten. Bei diesen Kursen ist es sehr wichtig, dass jede interessierte Frau teilnehmen kann. Deswegen sollen die Kurse ihren Wünschen, Bedürfnissen und Interessen so gut wie möglich entsprechen. Vielleicht wussten Sie das noch nicht, aber auch der Kurs an dem Sie teilnehmen, ist aus dem BIG-Projekt entstanden.

Um BIG weiter zu verbessern, möchten wir gerne mehr über Ihre Wünsche und Interessen erfahren. Außerdem interessiert uns, ob BIG bei Ihnen eine Wirkung erzielt hat, z.B. auf Ihre Gesundheit. In dem Fragebogen werden deshalb Fragen zu den Kursen und Ihrer persönlichen Situation gestellt. Wir bitten Sie diese Fragen zu beantworten.
Wir bedanken uns herzlich für Ihre Unterstützung.

Ihr BIG-Team.

wichtige Informationen:

- Bitte beantworten Sie die Fragen, wie es Ihrer persönlichen Meinung entspricht.
- Es gibt keine richtigen oder falschen Antworten.
- Ihre Angaben sind anonym. Es sind keine Rückschlüsse auf Ihre Person möglich.
- Wir versichern Ihnen, dass Ihre Angaben vertraulich behandelt werden.
- Ihre Teilnahme ist freiwillig.
- Sollten Sie eine Frage nicht verstehen oder beantworten wollen, lassen Sie diese aus.

K. **Kurspräferenzen**

Wir möchten in diesem Teil herausfinden, welche Wünsche Sie haben, wie BIG-Sportkurse gestaltet sein sollen. Ziel ist es, Kurse anzubieten, an denen Sie auch weiterhin gerne teilnehmen.

In den folgenden Fragen werden jeweils zwei Kurse gegenübergestellt. Die Kurse unterscheiden sich in den Kurskosten, den Kurszeiten, der Dauer des Geh-/Fahrwegs, der Berücksichtigung von Interessen und zusätzlichen sozialen Aktivitäten.

Alle BIG-Sportkurse dauern ungefähr 60 Minuten und finden in Räumlichkeiten statt, die von außen nicht einsehbar sind. Sie können alle BIG-Sportkurse in einer Schnupperstunde testen, bevor Sie sich für den Kurs entscheiden. In allen BIG-Sportkursen besteht die Möglichkeit einer Kinderbetreuung. Ein Kurs hat jeweils 10 Termine. Die Termine finden unter der Woche statt.

Bitte kreuzen Sie jeweils an, ob Sie lieber an Kurs A oder Kurs B teilnehmen würden.

Sie können entweder Kurs A oder Kurs B wählen. Sie können nicht einen Kurs aus einzelnen Eigenschaften zusammenstellen. Bitte wählen Sie immer den Kurs, den Sie bevorzugen beziehungsweise den Kurs, den Sie weniger ablehnen.

Hier finden Sie eine Beschreibung der Eigenschaften, welche die zwei Kurse haben können:

Kurszeiten: Wann der Kurs stattfindet. Mögliche Uhrzeiten sind morgens (8-10 Uhr), vormittags (11-12 Uhr), nachmittags (16-18 Uhr) und abends (18-20 Uhr).

Dauer Fahrweg: Wie lange Sie zu der Halle/dem Kursort brauchen (zu Fuß oder mit dem für Sie bequemsten Verkehrsmittel): die Möglichkeiten sind 10, 20 oder 30 Minuten.

Zusätzliche soziale Aktivitäten im Rahmen von BIG organisiert: Ob zusätzliche soziale Aktivitäten von BIG organisiert werden oder nicht (z.B. Frauenfrühstück oder gemeinsame Kochabende einmal im Monat).

Berücksichtigung von Interessen und Wünsche: Ob Ihre persönlichen Interessen oder Wünsche bei der Planung von Kursen abgefragt und (wenn möglich) berücksichtigt werden oder nicht.

Kurskosten pro Termin: Wie viel Sie für jeden Kurstermin zahlen müssen (in €). In unserem Fragebogen liegt der Preis der Kurse zwischen 2,00 und 9,50 Euro pro Termin.

Pedron S, *et al. BMJ Open* 2023; 13:e067235. doi: 10.1136/bmjopen-2022-067235

Bitte wählen Sie den Kurs aus, der Ihnen mehr zusagt. Bitte wählen Sie immer einen Kurs.

L1. Auswahlmöglichkeit	Kurs A	Kurs B
**Kurszeiten**	Vormittags (10-12 Uhr)	Morgens (8-10 Uhr)
**Dauer Geh-/Fahrweg bis zum BIG-Kurs**	Maximal 20 Minuten	Maximal 10 Minuten
**Zusätzliche soziale Aktivitäten im Rahmen von BIG organisiert**	Keine zusätzliche Aktivität	Zusätzliche soziale Aktivitäten
**Berücksichtigung von Interessen und Wünsche bei der weiteren Kursplanung**	Interessen/Wünsche werden berücksichtigt	Interessen/Wünsche werden nicht berücksichtigt
**Kurskosten (pro Termin)**	8,00 €	5,00 €
**Welchen Kurs bevorzugen Sie? (Kreuzen Sie bitte das entsprechende Kästchen an)**	☐	☐

Bitte wählen Sie den Kurs aus, der Ihnen mehr zusagt. Bitte wählen Sie immer einen Kurs.

L2. Auswahlmöglichkeit	Kurs A	Kurs B
**Kurszeiten**	Abends (18-20 Uhr)	Nachmittags (16-18 Uhr)
**Dauer Geh-/Fahrweg bis zum BIG-Kurs**	Maximal 30 Minuten	Maximal 20 Minuten
**Zusätzliche soziale Aktivitäten im Rahmen von BIG organisiert**	Keine zusätzliche Aktivität	Zusätzliche soziale Aktivitäten
**Berücksichtigung von Interessen und Wünsche bei der weiteren Kursplanung**	Interessen/Wünsche werden berücksichtigt	Interessen/Wünsche werden nicht berücksichtigt
**Kurskosten (pro Termin)**	5,00 €	9,50 €
**Welchen Kurs bevorzugen Sie? (Kreuzen Sie bitte das entsprechende Kästchen an)**	☐	☐

Bitte wählen Sie den Kurs aus, der Ihnen mehr zusagt. Bitte wählen Sie immer einen Kurs.

L3. Auswahlmöglichkeit	Kurs A	Kurs B
**Kurszeiten**	Abends (18-20 Uhr)	Vormittags (10-12 Uhr)
**Dauer Geh-/Fahrweg bis zum BIG-Kurs**	Maximal 20 Minuten	Maximal 10 Minuten
**Zusätzliche soziale Aktivitäten im Rahmen von BIG organisiert**	Keine zusätzliche Aktivität	Zusätzliche soziale Aktivitäten
**Berücksichtigung von Interessen und Wünsche bei der weiteren Kursplanung**	Interessen/Wünsche werden nicht berücksichtigt	Interessen/Wünsche werden berücksichtigt
**Kurskosten (pro Termin)**	3,50 €	9,50 €
**Welchen Kurs bevorzugen Sie? (Kreuzen Sie bitte das entsprechende Kästchen an)**	☐	☐

Pedron S, *et al. BMJ Open* 2023; 13:e067235. doi: 10.1136/bmjopen-2022-067235

Bitte wählen Sie den Kurs aus, der Ihnen mehr zusagt. Bitte wählen Sie immer einen Kurs.

L4. Auswahlmöglichkeit	Kurs A	Kurs B
**Kurszeiten**	Morgens (8-10 Uhr)	Vormittags (10-12 Uhr)
**Dauer Geh-/Fahrweg bis zum BIG-Kurs**	Maximal 30 Minuten	Maximal 10 Minuten
**Zusätzliche soziale Aktivitäten im Rahmen von BIG organisiert**	Keine zusätzliche Aktivität	Keine zusätzliche Aktivität
**Berücksichtigung von Interessen und Wünsche bei der weiteren Kursplanung**	Interessen/Wünsche werden berücksichtigt	Interessen/Wünsche werden nicht berücksichtigt
**Kurskosten (pro Termin)**	6,50 €	5,00 €
**Welchen Kurs bevorzugen Sie? (Kreuzen Sie bitte das entsprechende Kästchen an)**	☐	☐

Bitte wählen Sie den Kurs aus, der Ihnen mehr zusagt. Bitte wählen Sie immer einen Kurs.

L5. Auswahlmöglichkeit	Kurs A	Kurs B
**Kurszeiten**	Nachmittags (16-18 Uhr)	Abends (18-20 Uhr)
**Dauer Geh-/Fahrweg bis zum BIG-Kurs**	Maximal 10 Minuten	Maximal 30 Minuten
**Zusätzliche soziale Aktivitäten im Rahmen von BIG organisiert**	Keine zusätzliche Aktivität	Zusätzliche soziale Aktivitäten
**Berücksichtigung von Interessen und Wünsche bei der weiteren Kursplanung**	Interessen/Wünsche werden berücksichtigt	Interessen/Wünsche werden nicht berücksichtigt
**Kurskosten (pro Termin)**	3,50 €	8,00 €
**Welchen Kurs bevorzugen Sie? (Kreuzen Sie bitte das entsprechende Kästchen an)**	☐	☐

Bitte wählen Sie den Kurs aus, der Ihnen mehr zusagt. Bitte wählen Sie immer einen Kurs.

L6. Auswahlmöglichkeit	Kurs A	Kurs B
**Kurszeiten**	Nachmittags (16-18 Uhr)	Morgens (8-10 Uhr)
**Dauer Geh-/Fahrweg bis zum BIG-Kurs**	Maximal 30 Minuten	Maximal 10 Minuten
**Zusätzliche soziale Aktivitäten im Rahmen von BIG organisiert**	Zusätzliche soziale Aktivitäten	Keine zusätzliche Aktivität
**Berücksichtigung von Interessen und Wünsche bei der weiteren Kursplanung**	Interessen/Wünsche werden berücksichtigt	Interessen/Wünsche werden nicht berücksichtigt
**Kurskosten (pro Termin)**	2,00 €	9,50 €
**Welchen Kurs bevorzugen Sie? (Kreuzen Sie bitte das entsprechende Kästchen an)**	☐	☐

Bitte wählen Sie den Kurs aus, der Ihnen mehr zusagt. Bitte wählen Sie immer einen Kurs.

L7. Auswahlmöglichkeit	Kurs A		Kurs B
**Kurszeiten**	Morgens (8-10 Uhr)		Nachmittags (16-18 Uhr)
**Dauer Geh-/Fahrweg bis zum BIG-Kurs**	Maximal 20 Minuten		Maximal 30 Minuten
**Zusätzliche soziale Aktivitäten im Rahmen von BIG organisiert**	Zusätzliche soziale Aktivitäten		Keine zusätzliche Aktivität
**Berücksichtigung von Interessen und Wünsche bei der weiteren Kursplanung**	Interessen/Wünsche werden berücksichtigt		Interessen/Wünsche werden nicht berücksichtigt
**Kurskosten (pro Termin)**	2,00 €		6,50 €
**Welchen Kurs bevorzugen Sie? (Kreuzen Sie bitte das entsprechende Kästchen an)**	☐		☐

Bitte wählen Sie den Kurs aus, der Ihnen mehr zusagt. Bitte wählen Sie immer einen Kurs.

L8. Auswahlmöglichkeit	Kurs A		Kurs B
**Kurszeiten**	Vormittags (10-12 Uhr)		Morgens (8-10 Uhr)
**Dauer Geh-/Fahrweg bis zum BIG-Kurs**	Maximal 10 Minuten		Maximal 30 Minuten
**Zusätzliche soziale Aktivitäten im Rahmen von BIG organisiert**	Keine zusätzliche Aktivität		Keine zusätzliche Aktivität
**Berücksichtigung von Interessen und Wünsche bei der weiteren Kursplanung**	Interessen/Wünsche werden nicht berücksichtigt		Interesse/Wünsche werden berücksichtigt
**Kurskosten (pro Termin)**	5,00 €		6,50 €
**Welchen Kurs bevorzugen Sie? (Kreuzen Sie bitte das entsprechende Kästchen an)**	☐		☐

Bitte wählen Sie den Kurs aus, der Ihnen mehr zusagt. Bitte wählen Sie immer einen Kurs.

L9. Auswahlmöglichkeit	Kurs A		Kurs B
**Kurszeiten**	Vormittags (10-12 Uhr)		Abends (18-20 Uhr)
**Dauer Geh-/Fahrweg bis zum BIG-Kurs**	Maximal 30 Minuten		Maximal 10 Minuten
**Zusätzliche soziale Aktivitäten im Rahmen von BIG organisiert**	Zusätzliche soziale Aktivitäten		Zusätzliche soziale Aktivitäten
**Berücksichtigung von Interessen und Wünsche bei der weiteren Kursplanung**	Interessen/Wünsche werden nicht berücksichtigt		Interessen/Wünsche werden berücksichtigt
**Kurskosten (pro Termin)**	2,00 €		8,00 €
**Welchen Kurs bevorzugen Sie? (Kreuzen Sie bitte das entsprechende Kästchen an)**	☐		☐

References

Aboagye, E. (2017). Valuing Individuals' Preferences and Health Choices of Physical Exercise. *Pain Ther*, 6(1), 85-91. https://doi.org/10.1007/s40122-017-0067-4

Aboagye, E., Hagberg, J., Axén, I., Kwak, L., Lohela-Karlsson, M., Skillgate, E., Dahlgren, G., & Jensen, I. (2017). Individual preferences for physical exercise as secondary prevention for non-specific low back pain: A discrete choice experiment. *PLoS One*, 12(12), e0187709. https://doi.org/10.1371/journal.pone.0187709

Alayli-Goebbels, A. F., Dellaert, B. G., Knox, S. A., Ament, A. J., Lakerveld, J., Bot, S. D., Nijpels, G., & Severens, J. L. (2013). Consumer preferences for health and nonhealth outcomes of health promotion: results from a discrete choice experiment. *Value Health*, 16(1), 114-123. https://doi.org/10.1016/j.jval.2012.08.2211

Benning, T. M., Dellaert, B. G. C., & Arentze, T. A. (2020). The impact of health vs. non-health goals on individuals' lifestyle program choices: a discrete choice experiment approach. *BMC Public Health*, 20(1), 411. https://doi.org/10.1186/s12889-020-8416-3

Brown, D. S., Finkelstein, E. A., Brown, D. R., Buchner, D. M., & Johnson, F. R. (2009). Estimating older adults' preferences for walking programs via conjoint analysis. *Am J Prev Med*, 36(3), 201-207.e204. https://doi.org/10.1016/j.amepre.2008.10.014

Dintsios, C. M., Chernyak, N., Grehl, B., & Icks, A. (2018). Quantified patient preferences for lifestyle intervention programs for diabetes prevention-a protocol for a systematic review. *Syst Rev*, 7(1), 214. https://doi.org/10.1186/s13643-018-0884-5

Farooqui, M. A., Tan, Y. T., Bilger, M., & Finkelstein, E. A. (2014). Effects of financial incentives on motivating physical activity among older adults: results from a discrete choice experiment. *BMC Public Health*, 14, 141. https://doi.org/10.1186/1471-2458-14-141

Ferreira, G. E., Howard, K., Zadro, J. R., O'Keeffe, M., Lin, C. C., & Maher, C. G. (2020). People considering exercise to prevent low back pain recurrence prefer exercise programs that differ from programs known to be effective: a discrete choice experiment. *J Physiother*, 66(4), 249-255. https://doi.org/10.1016/j.jphys.2020.09.011

Geidl, W., Knocke, K., Schupp, W., & Pfeifer, K. (2018). Measuring stroke patients' exercise preferences using a discrete choice experiment. *Neurol Int*, 10(1), 6993. https://doi.org/10.4081/ni.2018.6993

Giles, E. L., Becker, F., Ternent, L., Sniehotta, F. F., McColl, E., & Adams, J. (2016). Acceptability of Financial Incentives for Health Behaviours: A Discrete Choice Experiment. *PLoS One*, 11(6), e0157403. https://doi.org/10.1371/journal.pone.0157403

Kjaer, T., Gyrd-Hansen, D., & Willaing, I. (2006). Investigating patients' preferences for cardiac rehabilitation in Denmark. *Int J Technol Assess Health Care*, 22(2), 211-218. https://doi.org/10.1017/s0266462306051038

Matsushita, M., Harada, K., & Arao, T. (2017). [Incentive program to strengthen motivation for increasing physical activity via conjoint analysis]. *Nihon Koshu Eisei Zasshi*, 64(4), 197-206. https://doi.org/10.11236/jph.64.4_197

Molema, C., Veldwijk, J., Wendel-Vos, W., de Wit, A., van de Goor, I., & Schuit, J. (2019). Chronically ill patients' preferences for a financial incentive in a lifestyle intervention. Results of a discrete choice experiment. *PLoS One*, 14(7), e0219112. https://doi.org/10.1371/journal.pone.0219112

Owen, K., Pettman, T., Haas, M., Viney, R., & Misan, G. (2010). Individual preferences for diet and exercise programmes: changes over a lifestyle intervention and their link with outcomes. *Public Health Nutr*, 13(2), 245-252. https://doi.org/10.1017/s1368980009990784

Paul, S. S., Canning, C. G., Löfgren, N., Sherrington, C., Lee, D. C., Bampton, J., & Howard, K. (2021). People with Parkinson's disease are more willing to do additional exercise if the exercise program has specific attributes: a discrete choice experiment. *J Physiother*, 67(1), 49-55. https://doi.org/10.1016/j.jphys.2020.12.007

Pinto, D., Bockenholt, U., Lee, J., Chang, R. W., Sharma, L., Finn, D. J., Heinemann, A. W., Holl, J. L., & Hansen, P. (2019). Preferences for physical activity: a conjoint analysis involving people with chronic knee pain. *Osteoarthritis Cartilage*, 27(2), 240-247. https://doi.org/10.1016/j.joca.2018.10.002

Pinto, D., Danilovich, M. K., Hansen, P., Finn, D. J., Chang, R. W., Holl, J. L., Heinemann, A. W., & Bockenholt, U. (2017). Qualitative Development of a Discrete Choice Experiment for Physical Activity Interventions to Improve Knee Osteoarthritis. *Arch Phys Med Rehabil*, 98(6), 1210-1216.e1211. https://doi.org/10.1016/j.apmr.2016.11.024

Ramirez, M., Wu, S., & Beale, E. (2016). Designing a Text Messaging Intervention to Improve Physical Activity Behavior Among Low-Income Latino Patients With Diabetes: A Discrete-Choice Experiment, Los Angeles, 2014-2015. *Prev Chronic Dis*, 13, E171. https://doi.org/10.5888/pcd13.160035

Robles, L. A., Wright, S. J., Hackshaw-McGeagh, L., Shingler, E., Shiridzinomwa, C., Lane, J. A., Martin, R. M., & Burden, S. (2020). Prostate cancer survivors' preferences on the delivery of diet and lifestyle advice: a pilot best-worst discrete choice experiment. *Pilot Feasibility Stud*, 6, 2. https://doi.org/10.1186/s40814-019-0549-8

Ryan, M., Yi, D., Avenell, A., Douglas, F., Aucott, L., van Teijlingen, E., & Vale, L. (2015). Gaining pounds by losing pounds: preferences for lifestyle interventions to reduce obesity. *Health Econ Policy Law*, 10(2), 161-182. https://doi.org/10.1017/s1744133114000413

Salampessy, B. H., Veldwijk, J., Jantine Schuit, A., van den Brekel-Dijkstra, K., Neslo, R. E., Ardine de Wit, G., & Lambooij, M. S. (2015). The Predictive Value of Discrete Choice Experiments in Public Health: An Exploratory Application. *Patient*, 8(6), 521-529. https://doi.org/10.1007/s40271-015-0115-2

Sommer, J., Dyczmons, J., Grobosch, S., Gontscharuk, V., Vomhof, M., Roden, M., & Icks, A. (2020). Preferences of people with type 2 diabetes for telemedical lifestyle programmes in Germany: protocol of a discrete choice experiment. *BMJ Open*, 10(9), e036995. https://doi.org/10.1136/bmjopen-2020-036995

van Gils, P. F., Lambooij, M. S., Flanderijn, M. H., van den Berg, M., de Wit, G. A., Schuit, A. J., Struijs, J. N., & van den Berg, B. (2011). Willingness to participate in a lifestyle intervention program of patients with type 2 diabetes mellitus: a conjoint analysis. *Patient Prefer Adherence*, 5, 537-546. https://doi.org/10.2147/ppa.S16854

Veldwijk, J., Lambooij, M. S., de Bekker-Grob, E. W., Smit, H. A., & de Wit, G. A. (2014). The effect of including an opt-out option in discrete choice experiments. *PLoS One*, 9(11), e111805. https://doi.org/10.1371/journal.pone.0111805

Veldwijk, J., Lambooij, M. S., van Gils, P. F., Struijs, J. N., Smit, H. A., & de Wit, G. A. (2013). Type 2 diabetes patients' preferences and willingness to pay for lifestyle programs: a discrete choice experiment. *BMC Public Health*, 13, 1099. https://doi.org/10.1186/1471-2458-13-1099

Wanders, J. O., Veldwijk, J., de Wit, G. A., Hart, H. E., van Gils, P. F., & Lambooij, M. S. (2014). The effect of out-of-pocket costs and financial rewards in a discrete choice experiment: an application to lifestyle programs. *BMC Public Health*, 14, 870. https://doi.org/10.1186/1471-2458-14-870
