# Supplementary material for: Preferences of women in difficult life situations for a physical activity programme: protocol of a discrete choice experiment in the German NU-BIG project
Source: BMJ Open. 2023 Jul 17;13(7):e067235. doi: 10.1136/bmjopen-2022-067235 (PMC10357700; doi:10.1136/bmjopen-2022-067235)
Supplement: Author's manuscript [file bmjopen-2022-067235.draft_revisions.pdf]

BMJ Open is committed to open peer review. As part of this commitment we make the peer review history of every article we publish publicly available.

When an article is published we post the peer reviewers' comments and the authors' responses online. We also post the versions of the paper that were used during peer review. These are the versions that the peer review comments apply to.

The versions of the paper that follow are the versions that were submitted during the peer review process. They are not the versions of record or the final published versions. They should not be cited or distributed as the published version of this manuscript.

BMJ Open is an open access journal and the full, final, typeset and author-corrected version of record of the manuscript is available on our site with no access controls, subscription charges or pay-per-view fees (http://bmjopen.bmj.com).

If you have any questions on BMJ Open's open peer review process please email info.bmjopen@bmj.com

BMJ Open

Preferences of women in difficult life situations for a physical activity program: protocol of a discrete choice experiment in the German NU-BIG project

Journal:	*BMJ Open*
Manuscript ID	bmjopen-2022-067235
Article Type:	Protocol
Date Submitted by the Author:	23-Sep-2022
Complete List of Authors:	Pedron, Sara; Technical University of Munich, Department of Sport and Health Sciences; Helmholtz Center Munich German Research Center for Environmental Health, Institute of Health Economics and Healthcare Management<br>Herbert-Maul, Annika; Friedrich-Alexander-Universitat Erlangen-Nurnberg, Department of Sport Science and Sport<br>Sauter, Alexandra; University of Regensburg, Department for Epidemiology and Preventive Medicine, Medical Sociology<br>Linder, Stephanie; Friedrich-Alexander-Universitat Erlangen-Nurnberg, Department of Sport Science and Sport<br>Sommer, Raluca; Friedrich-Alexander-Universitat Erlangen-Nurnberg, Department of Sport Science and Sport<br>Vomhof, Markus; Heinrich Heine University Düsseldorf; German Center for Diabetes Research<br>Gontscharuk, Veronika; Heinrich Heine University Düsseldorf; German Center for Diabetes Research<br>Abu-Omar, Karim ; Friedrich-Alexander-Universitat Erlangen-Nurnberg, Department of Sport Science and Sport<br>Thiel, Ansgar; Eberhard Karls Universitat Tubingen, Institute of Sports Science, Social and Public Health Sciences and Interfaculty Research Institute for Sport and Physical Activity<br>Ziemainz, Heiko; Friedrich-Alexander-Universitat Erlangen-Nurnberg, Department of Sport Science and Sport<br>Holle, Rolf; Ludwig-Maximilians-Universitat Munchen, Institute for Medical Informatics, Biometry and Epidemiology<br>Laxy, Michael; Technical University of Munich, Department of Sport and Health Sciences; Helmholtz Center Munich German Research Center for Environmental Health
Keywords:	HEALTH ECONOMICS, SPORTS MEDICINE, SOCIAL MEDICINE

SCHOLARONE™
Manuscripts

Preferences of women in difficult life situations for a physical activity program: protocol of a discrete choice experiment in the German NU-BIG project

Sara Pedron [a,b,c], Annika Herbert-Maul [d], Alexandra Sauter [e], Stephanie Linder [d], Raluca Sommer [d], Markus Vomhof [f,c,g], Veronika Gontscharuk [f,c,g], Karim Abu-Omar [d], Ansgar Thiel [h], Heiko Ziemainz [d], Rolf Holle [h] & Michael Laxy [a,b,c]*

[a] Professorship of Public Health and Prevention, Department of Sport and Health Sciences, Technical University of Munich, Germany
[b] Institute of Health Economics and Health Care Management, Helmholtz Zentrum München, German Research Center for Environmental Health (GmbH), Germany
[c] German Center for Diabetes Research, Neuherberg, Germany
[d] Department of Sport Science and Sport, Friedrich-Alexander-Universität Erlangen-Nuremberg, Erlangen, Germany
[e] Department for Epidemiology and Preventive Medicine, Medical Sociology, University of Regensburg, Regensburg, Germany
[f] Institute for Health Services Research and Health Economics, Centre for Health and Society, Medical Faculty and University Hospital Düsseldorf, Heinrich-Heine-University Düsseldorf, Duesseldorf, Germany.
[g] Institute for Health Services Research and Health Economics, German Diabetes Center (DDZ), Leibniz Center for Diabetes Research at the Heinrich-Heine-University Düsseldorf, Duesseldorf, Germany
[h] Institute of Sports Science, Social and Public Health Sciences and Interfaculty Research Institute for Sport and Physical Activity, Eberhard Karls University Tübingen, Tübingen, Germany
[h] Institute for Medical Informatics, Biometry and Epidemiology, University Hospital, Ludwig-Maximilian-University Munich, Munich, Germany

Sara Pedron (sara.pedron@tum.de)
Annika Herbert-Maul (annika.herbert-maul@fau.de)
Alexandra Sauter (alexandra.sauter@klinik.uni-regensburg.de)
Stephanie Linder (stephanie.linder@fau.de)
Raluca Sommer (raluca.sommer@fau.de)
Markus Vomhof (Markus.Vomhof@uni-duesseldorf.de)
Veronika Gontscharuk (Veronika.Gontscharuk@ddz.de)
Karim Abu-Omar (karim.abu-omar@fau.de)
Ansgar Thiel (ansgar.thiel@uni-tuebingen.de)
Heiko Ziemainz (heiko.ziemainz@fau.de)
Rolf Holle (rholle@ibe.med.uni-muenchen.de)
Michael Laxy (Michael.laxy@tum.de)

*Corresponding author:
Sara Pedron, PhD
Technical University of Munich, Professorship of Public Health and Prevention, Georg-Brauchle-Ring 60, 80992 München, Germany
Sara.pedron@tum.de
+49 (0) 89/289 24979

Word count: 4177

For peer review only

For peer review only - http://bmjopen.bmj.com/site/about/guidelines.xhtml

Abstract

Introduction: The BIG project was developed in 2005 as a community-based participatory research program to offer accessible opportunities for physical activity to women in difficult life situations. Since then the program has grown and is currently actively implemented at eight sites in Germany. Within the NU-BIG project, a systematic evaluation of BIG is currently being conducted. As part of this effort, we strive to understand the preferences of participating women for different aspects of the program, and to analyze their willingness to pay.

Methods and analysis: In this protocol we describe the development and evaluation of a discrete choice experiment (DCE) to investigate participants´ preferences for a physical activity program for women in difficult life situations. The experiment will be embedded in a questionnaire covering several aspects of participation to the program (e.g., reach, efficacy, further effects) and socioeconomic characteristics of all active participants. After a thorough search of the literature, BIG documents review and expert interviews, we identified 5 important attributes of the program: course times, travel time to the course venue, additional social activities organized by BIG, consideration of wishes and interests for the further planning of courses, costs per course unit. Thereafter, we piloted the experiment with a sample of participants from the target group. After data collection, the experiment will be analyzed using a conditional logit model. Furthermore, we will conduct a latent class analysis to assess eventual heterogeneity in preferences.

Ethics and dissemination: Understanding women's preferences will provide useful insights for the further development of the program and ultimately increase participation and retention. The questionnaire, the included DCE and the pretest on participants received ethical approval (Application Nr. 20-247_1-B). We plan to disseminate the results of the DCE in peer-reviewed journals, national conferences and among participants and program coordinators and organizers.

Keywords: discrete choice experiment, disadvantaged groups, physical activity, preferences, willingness to pay

For peer review only

For peer review only - http://bmjopen.bmj.com/site/about/guidelines.xhtml

Strengths and limitations

- We line out the details of a discrete choice experiment (DCE) to understand preferences and willingness to pay of women in difficult life situation for their sustained participation to physical activity courses
- The study lines out our plans of evaluating also dimensions of participatory planning and inclusion activities organized by the BIG program
- The program that is object of the DCE is a complex one, where many different attributes might play a role in driving women´s decisions to keep participating
- Since the target group consists of women who already participate in the program, we will analyze preferences for a sustained participation, but will not be able to evaluate preferences for starting anew in the offered physical activity courses

For peer review only

For peer review only - http://bmjopen.bmj.com/site/about/guidelines.xhtml

1. Introduction

Several studies have demonstrated that leisure time physical activity follows a socioeconomic gradient: individuals with a lower education, lower income and lower occupational position were consistently shown to engage less frequently in physical activity than their higher socioeconomic status (SES) counterparts [1–3]. In fact, socioeconomic factors are one of the major determinants of cardiovascular and metabolic conditions, which are among the most important drivers of morbidity and mortality worldwide [4]. The effect of socioeconomic factors on cardio-metabolic health is multifaceted, but runs mostly via physiological, psychosocial and behavioral factors, with varying degrees of influence in different contexts and life-stages [5].

In the German context, it has been shown that women from disadvantaged socioeconomic backgrounds are especially at risk for physical inactivity [6–9], probably because of the presence of several barriers (economic, time-related, cultural and social) that hamper their participation to available physical activity offers [10, 11]. Therefore, policies and programs with the aim of reducing such barriers and increasing the amount of leisure time physical activity, especially in socially disadvantaged populations, are needed. While the majority of programs involve individual cognitive-behavioral approaches to enhance physical activity [12], it has been argued that programs targeting structural aspects, instead of individual-level determinants, might prove more effective [13]. This is especially the case if such programs manage to activate already existing assets in the communities and increase the offer of targeted possibilities, for example by directly considering and overcoming actual and perceived barriers of the targeted groups [14].

One example of such programs from the German context is the "BIG" project ("Bewegung als Investition in Gesundheit" – "Movement as Investment in Health"). The program is based on a community-based participatory research approach, aiming at involving the communities to shape and manage physical activity courses for women in difficult life situations, e.g. having a low household income, a migration background, are unemployed, rely on welfare aid, or are single-raising mothers [15, 16]. The program has since expanded to other twenty communities (eight of them are still active). In each one of these, a "cooperative planning approach" was adopted, where women from the target group were explicitly and regularly included in the planning and organization of the courses, with the aim of overcoming barriers, increase participation and retention [17–22].

Even though the project strives to include women from the target group in the planning and organizational phase, little is known about their decision-making process regarding a sustained

For peer review only - http://bmjopen.bmj.com/site/about/guidelines.xhtml

participation in the program. Understanding and evaluating the preferences of participating women is important in order to develop the program further, make it more diversified to reach specific groups of the population, and encourage participation over a longer period of time. In order to elicit patient or participant preferences, economic stated-preferences methods such as discrete choice experiments (DCE) have been increasingly used in healthcare contexts [23]. This preference elicitation method relies on the economic theory of demand [24] and on random utility theory [25, 26]. According to these theories, when confronted with a choice, individuals will choose the option with the highest level of utility, i.e. the option whose characteristics combined generate the highest utility value for the individual. DCE are a choice-based method where individuals are confronted with a series of hypothetical choice scenarios to simulate realistic choices. Respondents have to trade-off one option with all its characteristics with a second option, finally indicating which one of the two they prefer. By evaluating the final choice pattern, the method allows the computation of the relative importance of attributes. If an attribute representing costs is also assessed, then the willingness to pay (WTP) for levels can be elicited, i.e. how much a participant on average is willing to pay for an improvement in one attribute [27].

In order to deepen our understanding of the decision-making process of women regarding their sustained participation in the program, we aim at carrying out a DCE, targeted at understanding and quantifying the preferences of participating women for specific characteristics of the program and investigate how they differ with respect to socio-demographic and cultural backgrounds. The aim of the present protocol is to line out the preparatory steps for the DCE and to present the final version of the experiment used in the questionnaire. This DCE is the first one to evaluate the participatory aspect and the social component of a community-based participatory research program in the physical activity promotion field. The results will not only help to capture the decision-making process of women from disadvantaged social backgrounds for participation in physical activity, but will also help to further develop the program in the active BIG sites and help to achieve a greater involvement of participating women, thus fostering their integration and the benefits from participation. Finally, identifying components that are essential or highly valuated for the target group can be useful to inform policymakers to design better strategies for physical activity promotion for women in difficult life situations.

2. Methods and Analysis

2.1 The BIG project

For peer review only - http://bmjopen.bmj.com/site/about/guidelines.xhtml

The BIG project ("Bewegung als Investition in Gesundheit", i.e. "Movement as Investment in Health") was started in 2005 by the Friedrich-Alexander Universität Erlangen-Nürnberg (FAU), Germany. The project specifically aims at increasing physical activity among women in difficult life situations [15]. Since its start, the program has been transferred to other twenty communities/municipalities, mostly but not only in Bavaria (Germany) [17]. Currently the program is actively implemented at eight sites, with three sites in the starting phase. In the remaining nine sites the program was stopped because of different implementation and sustainability issues in the local communities.

The target group of the BIG programs are women in difficult life situations, which include women with a low income, a migration background, unemployed women, women relying on welfare aids or single mothers. However, BIG courses are open to all women who decide to take part in them.

Using a "cooperative planning approach" [18–21], the program aims at involving women from the target group also in the planning, organization and evaluation stages of the project. This way, highly tailored exercise programs could be constructed by eliminating barriers (e.g. by offering childcare possibilities during the course, by offering courses which respect religious norms, etc.). Ultimately, the program aims at maximizing participation and inclusion of women, thereby achieving a broader empowerment, with potential effects on self-efficacy and other health behaviors (e.g. healthy diet and smoking). Furthermore, this approach facilitates the inclusion of other women from their social network with a similar socioeconomic background, fostering integration and social exchange within the program itself.

At each site, the program offers several courses, both in gyms/halls (fitness courses) and in swimming pools (water courses). The fitness courses include for example Pilates, yoga, and aerobics. The water courses include water-fitness and swimming courses. Furthermore, at some sites, additional social and cultural activities are organized, such as a "breakfast for women" ("Frauenfrühstück") several times per year, cook-ins evenings with healthy dishes from different cultures and further occasions for mutual exchange. Given the participatory nature of the program and the active role that women have in shaping its contents and forms, the offered courses and additional activities differ from site to site (e.g. workshops for conflict management, stress reduction or self-defense) [17]. A detailed overview of the types of courses offered in each active site in the last year before the pandemic (2019), the number of participants for each course and the additional activities can be found in Appendix 1.

For the success of the program, women from the target group should be reached by these offers and should agree to participate. Furthermore, another very important aspect is their continued

For peer review only - http://bmjopen.bmj.com/site/about/guidelines.xhtml

participation, since only via a sustained participation can health improvements be achieved. Therefore, understanding the factors that drive their sustained participation (for example, via a DCE) is of paramount importance.

2.2 The NU-BIG evaluation and the target group

The present DCE is part of a general effort of evaluating the impact of the BIG program, called the NU-BIG study ("Nachuntersuchung des BIG-Projekts"), funded by the German Federal Ministry of Education and Research. The evaluation will follow the RE-AIM framework and was described in detail in the study protocol [28]. The evaluation includes investigating the long-term effects of BIG on health behavior, inclusion and social participation, as well as factors facilitating long-term implementation and transfer. The study consists of a cross-sectional evaluation using a mixed-methods approach, including questionnaires for participating women, qualitative interviews for local organizers and focus groups with participating women.

The present DCE will be part of the questionnaire, which will be distributed to all women who participate in BIG courses between May-December 2022 in active sites (thus excluding sites in the starting phase). Considering pre-pandemic participation rates, it was estimated that the study population will amount to around 800 participating women at eight sites. The questionnaire will be administered as "paper and pen" survey, by handing the paper questionnaire to the target group in person during the BIG-courses. Due to the diversity of BIG-activities at each site, an adjustment of the mode of administration will be required for several courses. In consultation with the trainers of each course, we will adapt the way we distribute the questionnaire to the specific requirements of each course (e.g., outdoor courses, swimming classes) and to the specific linguistic background of participating women by making the questionnaire available in German, Arabic, English, Russian and Turkish.

2.3 Development of the DCE to assess participant preferences

To derive preferences it is important to carefully develop and the transfer the DCE into an experimental design following methodological standards [25]. This involves a thorough identification of attributes and levels and the construction of choice sets within the experimental design. In the next paragraphs, we describe this process in detail in the context of the NU-BIG study.

2.3.1 Problem and target group definition

For peer review only - http://bmjopen.bmj.com/site/about/guidelines.xhtml

The main aim of the DCE is understanding which aspects of the program favor a sustained participation to the program and help keeping participating women interested in new courses. The target group consists of all women who are actively participating in BIG courses, since the questionnaire will be handed in during classes starting from the month of May 2022 until the end of December 2022.

2.3.2 Identification of attributes and levels and transfer to the experimental design

The DCE will be embedded in the questionnaire and will present women with a series of hypothetical comparisons between two physical activity courses with predefined characteristics, asking them to choose which option they like the most.

In a first step, all program characteristics (attributes) that might influence individual preferences for participation in BIG courses should be identified. At the same time, appropriate levels of these attributes should be determined. All included attributes should be relevant for the choice of the course, should be easily influenced and close to reality, should have a substitutive relation and should not be dominant [29]. To identify attributes and their relative levels we followed a systematic approach. We describe all steps below.

Compilation of evidence (literature search)

The literature was searched for physical activity programs/interventions, which were evaluated using a DCE or conjoint analysis. The full search strategy, flow chart and results can be browsed in Appendix 2. We identified 19 studies, of which we systematically retrieved attributes and corresponding levels. We divided these attributes in meaningful groups: exercise frequency and duration, context, type of exercise, outcomes and goals, schedule, costs, travel time and location, support and other. It is important to note, that no study specifically included exclusively women as a target group.

Compilation of evidence (program specific)

We reviewed previous documents of the BIG-program (qualitative and quantitative data, protocol of the kick-off meeting) to identify potential attributes and levels in addition to those identified by the literature search. Each document was searched independently from two researchers (from the group AS, SL, AHM, SP). Resulting attributes and levels were then pooled, discussed by the group (SP, AHM, SL, AS) and compared with the results from the systematic literature search (full list available in Appendix 2).

For peer review only - http://bmjopen.bmj.com/site/about/guidelines.xhtml

In a further step, we selected only attributes that are applicable in the context of the BIG courses and that are already implemented or potentially implementable in the program. For example, attributes and levels which included mixed classes with men or individual courses were excluded since BIG offers only group courses for women. Furthermore, attributes involving the presence of a personal coach were also excluded since this is not intended nor feasible in BIG. Additionally, attributes referring to programs targeting specific illnesses were also excluded. This selection led to a list of ten potentially important attributes (Appendix 3), which served as basis for discussion with the experts in the following step.

Consultation of experts

This step involved an online discussion of the previously identified list of ten attributes and their respective levels with five "BIG-experts". They are BIG course instructors, organizers or long-term participants, who actively took part in shaping BIG courses at their site and who are routinely interviewed and involved in the planning phases of the NU-BIG evaluation.

In the meeting, which was headed by SP, AHM and AS, we explained to the experts the goals of the DCE and we presented them with a first example of trade-off decision within a fictitious choice set. After this, each attribute and the respective level was thoroughly discussed in the group. Thereby we specifically asked if the ten reported characteristics are relevant ones and whether they represent important drivers in the decision-making process of participating women. Furthermore, we discussed their clarity and understandability and we explicitly asked if some important aspects might be missing. At the end of the meeting, we also asked the five experts to rank the attributes based on the perceived relevance for participating women with an interactive tool to spark further discussion.

The discussion led to interesting insights in the decision-making process of participating women, from the point of view of the course instructors and organizers. Following this discussion, one additional attribute was identified as important one ("flexible intensity of the course") and two others were re-framed in a different way. We report the results of this discussion, including the new list of attributes and levels, in Appendix 3.

Based on these insights, the list of eleven attributes was further analyzed by researchers (SP, AHM, MV). In our screening of the attributes we ensured that all attributes were relevant for the choice of a course and were easily influenced within the BIG program. Furthermore, we also ensured that they were in a substitutive relation (i.e. the worsening of one attribute can be compensated by an improvement in another attribute) and were not dominant (i.e. no attribute should be a necessary criterion for or against participation) [29]. The detailed decision steps

For peer review only - http://bmjopen.bmj.com/site/about/guidelines.xhtml

and the resulting list of attributes and levels are reported in Appendix 3. The resulting list includes five attributes, i.e. (1) course time, (2) travel time to the BIG course, (3) additional activities organized by BIG, (4) consideration of wishes or interests and (5) costs per course unit. Based on the discussion with the experts, we decided to keep some attributes fixed, i.e. to introduce an initial statement clarifying that all courses would have specific characteristics. These include the presence of childcare, the presence of trial lessons, course duration, and the framework (e.g. hall is not visible from the outside). These fixed attributes are in fact usual component of all BIG courses that are already implemented to reduce barriers to participation.

Pre-test with participants

Based on the selected attributes and levels, a preliminary experimental design was conducted to prepare a full DCE for a pre-test. We tested the full DCE questionnaire to a set of voluntary participants from the target group. For this aim, we recruited participating women and performed semi-structured qualitative interviews using the think aloud technique based on a topic guide until a saturation of new content was achieved [30, 31]. This led us to include 6 women from two BIG sites (Erlangen and Nürnberg). The interviews were carried out by telephone and were recorded after asking for consent of the participants. The interviewee was asked to read a paper version of the questionnaire that was previously sent by mail, and loudly report her thoughts and choices. The notes and recordings of the phone call were then revised by the interviewer (SP) and by a second researcher (MV), in order to identify the main problems and improvements necessary.

In the pre-test with participants we particularly paid attention to the following criteria:

(1) Are all the most important attributes included in the DCE?
(2) Is one or more of the included attributes dominant?
(3) Are all levels appropriate?
(4) Are the task and the questionnaire understandable and feasible?

The interviews followed a pre-specified outline and were recorded for quality assurance and analyzed using content analysis methods by two researchers (SP and MV) [32]. This step led to a general confirmation of the understandability of the experiment among women with different backgrounds, linguistic skills and from different cities. The task was understood properly and all women engaged in the tradeoffs proposed in the DCE with ease. Drawing from the feedback of participating women, we made some adjustments in missing context information (e.g., the courses take place only during the week, the number of appointments was specified).

For peer review only

For peer review only - http://bmjopen.bmj.com/site/about/guidelines.xhtml

Table 1: Attributes and levels included

Attributes	Levels
**Course time**	Early morning (8-10 am)
	Morning (10 am -12 pm)
	Afternoon (4-6 pm)
	Evening (6-8 pm)
**Travel time to the BIG course**	Maximum 10 Minutes
	Maximum 20 Minutes
	Maximum 30 Minutes
**Additional activities by BIG**	No additional social activity is organized
	Additional social activities are organized
**Consideration of wishes and interests for the further planning of courses**	Interests/Wishes are not asked/considered
	Interests/Wishes are asked/considered
**Costs per course unit**	2,00 €
	3,50 €
	5,00 €
	6,50 €
	8,00 €
	9,50 €

Notes: the original version in the German language is available in Appendix 4.

only

Furthermore, we modified the range of costs per training unit proposed in the DCE. Initially we considered a price range between 0 € and 5 € per training unit, with 1 € intervals (the levels were: 0, 1, 2, 3, 4, 5 €). However, the price was perceived as an irrelevant attribute by the participants, since even the highest level was considered very affordable and comparable with the levels below. Based on specific indications of participating women on the price of courses that they currently participate in (up to 8€ per course unit), we decided to increase the price range up to 9,5 €. Furthermore, we received mixed feedbacks regarding including the level 0 €: while some women perceived this as very advantageous, some others interpreted this as a sign of bad quality. Since we assume linearity in the cost attribute, we decided to eliminate this level. The final cost levels thus range between 2 € and 9,5 €, with 1,5 € intervals.

Final attributes and levels included

The final list of attributes and levels included in the DCE is reported in Table 1 (original language version in Appendix 4). We included 5 attributes, with varying number of levels. The

For peer review only - http://bmjopen.bmj.com/site/about/guidelines.xhtml

first attribute is the course time, i.e. when the course takes place during the day. For this attribute we considered four levels: early morning, morning, afternoon and evening. The second attribute represents the travel time needed to reach the course location. This encompasses three levels: maximum 10, 20 or 30 minutes. The third attribute represents the possibility of organized social activities within the BIG course, such as a "women´s breakfast" or joint dinners where all participants of the course are invited to participate to socialize beyond the course. This attribute has two levels: activities are organized or not. The fourth attribute considers the possibility of listening to participants´ interests and wishes, to be considered in the further planning of courses. Also this attribute is a binary one, either interests/wishes are considered or not. The final attribute is the costs of a course unit, ranging from 2 € to 9,5 € in 1,5 € intervals.

2.3.3 DCE questionnaire design and construction of choice sets

We constructed the final DCE experimental design following best practice guidelines [33]. The number of attributes and levels included in our DCE would generated 288 potential combinations (2^2 x 3 x 4 x 6). The attribute costs per course unit entered were considered as a continuous variable in the experimental design. Due to a decentralized administration of questionnaires, we were not able to implement a block design. Since the number of possible choice scenarios is very large, we used a D-efficient design to select the most efficient combination of choice sets, while at the same time allowing the estimation of all the main effects [23, 34].

We employed SAS macros (SAS V.9.4) by Kuhfeld 2010 [35] to derive the optimal number of choice sets. The final efficient factorial fractional design (D-Efficiency=5.8409, Relative D-Efficiency=73.0117, D-error=0.1712) consisted of eight unique choice tasks.

All attributes and level combinations were checked for plausibility. In order to test reliability of the choices, we repeated one item (choice set XX), leading to nine choice sets included in the final DCE. Participants are asked to choose between course A and course B. In order to keep the cognitive effort to a minimum, we did not include any opt-out option.

2.3.4 The DCE questionnaire

The DCE will be included in the NU-BIG paper-based questionnaire, after a general questionnaire introduction and questions regarding health behavior, health status, self-efficacy and integration and sociodemographic aspects. The DCE includes one page of detailed information on the content of the DCE. Participants are asked to choose out of two options the

For peer review only - http://bmjopen.bmj.com/site/about/guidelines.xhtml

one they prefer most, i.e. the one they would less likely reject. An example of choice set is presented in Fig.1.

*** Insert Fig. 1 about here ***

2.3.5 Data analysis

The included population will be described with respect to their sociodemographic characteristics drawing from the information collected in the NU-BIG questionnaire.

In order to estimate the preference weight of each level within each attribute we will analyze the DCE data using a conditional logit model. Thereby we will calculate the relative importance of attributes, using an effects-coding procedure [36]. In a further step, we will express the preference weights as money equivalents (i.e. as the marginal rate of substitution between the attributes and the cost attribute), in order to compute the marginal willingness to pay for a change in the level of the attributes [27].

The target population of women who participates to BIG is likely to be a heterogeneous one, especially with regard to sociodemographic characteristics, which might impact preferences (e.g. presence of children in the household, migration background and linguistic barriers, religious affiliation etc.). Therefore, we will carry out a latent class analysis in order to explore the underlying heterogeneity and identify classes of women with different preferences. We will also analyze whether these classes will be different according to age, employment status, education, number of children, marital status, health status, migration status and religious affiliation [27].

2.3.6 Sample size calculation

All participating women in the active BIG sites will be offered the chance to participate to the NU-BIG survey. Therefore, we will have limited possibilities of influencing the sample size for the DCE. Nevertheless, computing the minimum required size of the sample, also taking into account different response rates, is crucial for ensuring the feasibility of the DCE.

In order to compute the minimum sample size, we used the following rule of thumb [37, 38]:

$$n > \frac{1000\ c}{t\ \times a}$$

Here n is the number of participants, c is the highest number of levels (in our case, c=4), t is the number of choice tasks (in our case, t=8) and a is the number of alternatives per choice task (in our case, a=2). In our case, this formula yields a minimum of 250 participants.

For peer review only - http://bmjopen.bmj.com/site/about/guidelines.xhtml

Based on participation prior to the Covid-19 pandemic, the number of participants to the BIG courses was estimated around 800. However, the context and participation are likely to be different now, so that a conservative approach to the estimated target group is necessary. Even though assuming a 50% reduction in the sample, or in the response rate, the sample size is still higher than the minimum suggestion of 250.

2.3.7 Participants and public involvement

The BIG project is carried out using a "cooperative planning approach" [15], where women from the target group are involved in the planning and implementation of physical activity courses. Furthermore, in the NU-BIG project, as a comprehensive evaluation of BIG, the point of view of women from the target group was always taken into account by regularly involving the group of "BIG experts" in every step of the project for targeted feedback [17, 28].

Concretely, the idea for the DCE sparked from the meetings with the "BIG experts", as a way of formally investigating the preferences of participating women. Both the "BIG experts" and women from the target group of participants were involved in setting up of the DCE in the pre-test phases. Their helpful critical feedback was used to further develop the instrument.

3. Ethics and dissemination

The NU-BIG project was approved by the Ethics Commission of the Friedrich-Alexander-University of Erlangen-Nurnberg (Application Nr. 20-247_1-B). The DCE described in this protocol was included as part of the NU-BIG project and described in detail, including also recruitment for the pre-test with women from the target group.

All women participating in the pre-test read an extensive information sheet regarding the DCE and gave written informed consent and received a compensation (20 €). All participants to the final NU-BIG paper questionnaire will receive an information sheet and will be required to sign an informed consent form.

The DCE questionnaire and the relative data analysis will be carried out following the principles of good scientific practice [27, 33]. The results will not only be disseminated in academic journals and conferences, but also among participants groups and organizing bodies, with the explicit aim of improving the courses offered by BIG in all sites.

For peer review only - http://bmjopen.bmj.com/site/about/guidelines.xhtml

Funding: This work was supported by the German Federal Ministry of Education and Research (grant no. 01EL20122D)

Author Contributions: SP and ML identified the research question. SP, MV and VG planned the described DCE in all phases. SP, AHM, AS and SL carried out the necessary steps for the identification of potential attributes and interviewed the experts. SP and AS carried out the pre-test with participants. SP wrote a first draft of the manuscript, all authors read, commented and approved the manuscript. KAO, HZ, AHM and RH were responsible for funding application and work as coordinators of the NU-BIG evaluation. They contributed with expert knowledge to the identification of the research question, the setup of the DCE and the final manuscript.

Competing interests statement: the authors declare no competing interests.

Data statement section: after publication of the main analysis paper, all data will be made available upon request and after anonymization to researchers who provide a sound analysis plan to replicate our results.

For peer review only

For peer review only - http://bmjopen.bmj.com/site/about/guidelines.xhtml

References

1. Gidlow C, Johnston LH, Crone D, Ellis N, James D. A systematic review of the relationship between socio-economic position and physical activity. Health Educ J. 2016;65:338–67.

2. Beenackers MA, Kamphuis CB, Giskes K, Brug J, Kunst AE, Burdorf A, et al. Socioeconomic inequalities in occupational, leisure-time, and transport related physical activity among European adults: A systematic review. Int J Behav Nutr Phys Act 2012 91. 2012;9:1–23.

3. Abu-Omar K, Messing S, Sarshar M, Gelius P, Ferschl S, Finger J, et al. Sociodemographic correlates of physical activity and sport among adults in Germany: 1997–2018. Ger J Exerc Sport Res. 2021;51:170–82.

4. Petrovic D, de Mestral C, Bochud M, Bartley M, Kivimäki M, Vineis P, et al. The contribution of health behaviors to socioeconomic inequalities in health: A systematic review. Prev Med (Baltim). 2018;113:15–31.

5. Ben-Shlomo Y, Kuh D. A life course approach to chronic disease epidemiology: conceptual models, empirical challenges and interdisciplinary perspectives. Int J Epidemiol. 2002;31:285–93.

6. Rütten A, Abu-Omar K, Adlwarth W, Meierjürgen R. Bewegungsarme lebensstile - Zur klassifizierung unterschiedlicher zielgruppen für eine gesundheitsförderliche körperliche aktivierung. Gesundheitswesen. 2007;69:393–400.

7. Hoebel J, Finger JD, Kuntz B, Kroll LE, Manz K, Lange C, et al. Changing educational inequalities in sporting inactivity among adults in Germany: a trend study from 2003 to 2012. BMC Public Health. 2017;17:1–10.

8. Krug S, Jordan S, Mensink GBM, Müters S, Finger J, Lampert T. Körperliche Aktivität: Ergebnisse der Studie zur Gesundheit Erwachsener in Deutschland (DEGS1). Bundesgesundheitsblatt - Gesundheitsforsch - Gesundheitsschutz. 2013;56:765–71.

9. Koch-Institut R. Gesundheitliche Lage der Frauen in Deutschland | 2020.

10. Frahsa A, Streber A, Wolff AR, Rütten A. Capabilities for Physical Activity by Turkish- and Russian-Speaking Immigrants Aged 65 Years and Older in Germany: A Qualitative Study. J Aging Phys Act. 2020;28:567–79.

For peer review only - http://bmjopen.bmj.com/site/about/guidelines.xhtml

For peer review only

11. Withall J, Jago R, Fox KR. Why some do but most don't. Barriers and enablers to engaging low-income groups in physical activity programmes: A mixed methods study. BMC Public Health. 2011;11:1–13.

12. Rütten A, Schow D, Breda J, Galea G, Kahlmeier S, Oppert JM, et al. Three types of scientific evidence to inform physical activity policy: results from a comparative scoping review. Int J Public Health. 2016;61:553–63.

13. Rutter H, Bes-Rastrollo M, De Henauw S, Lahti-Koski M, Lehtinen-Jacks S, Mullerova D, et al. Balancing Upstream and Downstream Measures to Tackle the Obesity Epidemic: A Position Statement from the European Association for the Study of Obesity. Obes Facts. 2017;10:61–3.

14. Rütten A, Abu-Omar K, Frahsa A, Morgan A. Assets for policy making in health promotion: Overcoming political barriers inhibiting women in difficult life situations to access sport facilities - ScienceDirect. Soc Sci Med. 2009;69:1667–73.

15. Rütten A, Röger U, Abu-Omar K, Frahsa A. Empowerment von frauen in sozial benachteiligten lebenslagen: Das BIG-projekt. Gesundheitswesen. 2008;70:742–7.

16. Röger U, Rütten A, Frahsa A, Abu-Omar K, Morgan A. Differences in individual empowerment outcomes of socially disadvantaged women: Effects of mode of participation and structural changes in a physical activity promotion program. Int J Public Health. 2011;56:465–73.

17. Herbert-Maul A, Abu-Omar K, Frahsa A, Streber A, Reimers AK. Transferring a Community-Based Participatory Research Project to Promote Physical Activity Among Socially Disadvantaged Women—Experiences From 15 Years of BIG. Front Public Heal. 2020;8:546.

18. Haldane V, Chuah FLH, Srivastava A, Singh SR, Koh GCH, Seng CK, et al. Community participation in health services development, implementation, and evaluation: A systematic review of empowerment, health, community, and process outcomes. PLoS One. 2019;14:e0216112.

19. O'Mara-Eves A, Brunton G, Oliver S, Kavanagh J, Jamal F, Thomas J. The effectiveness of community engagement in public health interventions for disadvantaged groups: A meta-analysis. BMC Public Health. 2015;15:1–23.

20. Rütten A, Gelius P. Building policy capacities: an interactive approach for linking

For peer review only - http://bmjopen.bmj.com/site/about/guidelines.xhtml

knowledge to action in health promotion. Health Promot Int. 2014;29:569–82.

21. Gelius PC, Jansen M, King AC. Cooperative planning and its utilization in German physical activity promotion: a brief introduction. Health Promot Int. 2021;36 Supplement_2:ii1–7.

22. Sanz-Remacha M, García-González L, Sevil Serrano J, Aibar Solana A. A Qualitative Evaluation of a Community-Based Intervention on Health-Related Behaviors in Disadvantaged Women. Res Q Exerc Sport. 2022. https://doi.org/10.1080/02701367.2021.1971149.

23. Bekker-Grob EW de, Ryan M, Gerard K. Discrete choice experiments in health economics: a review of the literature. Health Econ. 2012;21:145–72.

24. Lancaster KJ. A New Approach to Consumer Theory. J Polit Econ. 1966;74:132–57.

25. Louviere JJ, Hensher DA, Swait JD, Adamowicz W. Stated Choice Methods: Analysis and Applications. Cambridge University Press; 2000.

26. Lancsar E, Savage E. Deriving welfare measures from discrete choice experiments: inconsistency between current methods and random utility and welfare theory. Health Econ. 2004;13:901–7.

27. Hauber AB, González JM, Groothuis-Oudshoorn CGM, Prior T, Marshall DA, Cunningham C, et al. Statistical Methods for the Analysis of Discrete Choice Experiments: A Report of the ISPOR Conjoint Analysis Good Research Practices Task Force. Value Heal. 2016;19:300–15.

28. Abu-Omar K, Ziemainz H, Loss J, Laxy M, Holle R, Thiel A, et al. The Long-Term Public Health Impact of a Community-Based Participatory Research Project for Health Promotion Among Socially Disadvantaged Women—A Case Study Protocol. Front Public Heal. 2021;9:317.

29. Telser H. Nutzenmessung im Gesundheitswesen: Die Methode der Discrete-Choice-Experimente. Hamburg: Verlag Dr. Kovač; 2002.

30. Drennan J. Cognitive interviewing: verbal data in the design and pretesting of questionnaires. J Adv Nurs. 2003;42:57–63.

31. Charters E. The Use of Think-aloud Methods in Qualitative Research An Introduction to Think-aloud Methods. Brock Educ J. 2003;12.

For peer review only

32. Clarke V, Braun V. Thematic Analysis. Encycl Crit Psychol. 2014;:1947–52.

33. Johnson FR, Lancsar E, Marshall D, Kilambi V, Mühlbacher A, Regier DA, et al. Constructing Experimental Designs for Discrete-Choice Experiments: Report of the ISPOR Conjoint Analysis Experimental Design Good Research Practices Task Force. Value Heal. 2013;16:3–13.

34. Lancsar E, Louviere J. Conducting discrete choice experiments to inform healthcare decision making: A user's guide. Pharmacoeconomics. 2008;26:661–77.

35. Kuhfeld WF. Marketing Research Methods in SAS: Experimental Design, Choice, Conjoint, and Graphical Techniques. MR-2010. 2010.

36. Bech M, Gyrd-Hansen D. Effects coding in discrete choice experiments. Health Econ. 2005;14:1079–83.

37. de Bekker-Grob EW, Donkers B, Jonker MF, Stolk EA. Sample Size Requirements for Discrete-Choice Experiments in Healthcare: a Practical Guide. Patient. 2015;8:373–84.

38. Orme BK. Getting started with conjoint analysis : strategies for product design and pricing research. 2nd edition. Madison, WI: Research Publishers; 2010.

For peer review only

For peer review only - http://bmjopen.bmj.com/site/about/guidelines.xhtml

Figure legends

Fig. 1: Example of choice set

Note: own translation from German.

For peer review only

For peer review only - http://bmjopen.bmj.com/site/about/guidelines.xhtml

	Course A	Course B
**Course times**	Morning (10 am – 12 pm)	Early morning (8-10 am)
**Travel time to BIG course**	Max. 20 minutes	Max. 10 minutes
**Additional activities orgsnized by BIG**	No additional activity	Additional social activities
**Consideration of wishes and interests for the further planning of courses**	Interests/wishes are considered	Interests/wishes are not considered
**Costs per course unit**	8,0 €	5,0 €
**Which course do you prefer? (Please cross the relative box)**	☐	☐

Fig. 1: Example of choice set

318x117mm (120 x 120 DPI)

Preferences of women in difficult life situations for a physical activity program: protocol of a discrete choice experiment in the NU-BIG project

Sara Pedron, Annika Herbert-Maul, Alexandra, Stephanie Linder, Raluca Sommer, Markus Vomhof, Veronika Gontscharuk, Karim Abu-Omar, Ansgar, Heiko Ziemainz, Rolf Holle & Michael Laxy

Supplementary material

For peer review only

Appendix 1: course description in each BIG-site

Table A.1: description of courses in active sites in 2019

	time unit	Number of participants	Number of fitness courses	Number of water courses	Additional offers	Additional social activities	Planning & participation	mean number of participants per course
Erlangen	year	695	57	13	women-only bathing time, additional sport activities (dancing 2x per year, archery, climbing,...)	women´s breakfast (8x year), healthy diet course (2x year), arabic cuisine tasting (1x year)	Planning meeting (2x year)	10
Regensburg	I	166	34	3	women-only bathing time, biking course			10
	II	132						
	III	88						
Bayreuth	I	127	18	8	biking course			13
	II	112						
	III	108						
Straubing		144	10	0	boxing	integration course		14
Berlin (Treptow-Köpenick)		4	1					4
Nürnberg	I	63	0	3				21
	II	61	0	3				
	III	81	0	4				
Neustadt a.d. Aisch		20		2				10

Note: the table shows the number of participants in each active site in 2019, either for the whole years or for different course seasons (I, II or III).

review only

For peer review only - http://bmjopen.bmj.com/site/about/guidelines.xhtml

Appendix 2: Systematic literature search protocol and results

Goal: finding studies which evaluated individual preferences for lifestyle programs/physical activity programs

Search in PubMed:

(physical activity[Title/Abstract]) AND (discrete choice[Title/Abstract])

((physical activity[Title/Abstract]) AND (conjoint analysis[Title/Abstract])

(lifestyle[Title/Abstract]) AND (discrete choice[Title/Abstract])

(coaching[Title/Abstract]) AND (discrete choice[Title/Abstract])

(coaching[Title/Abstract]) AND (conjoint analysis[Title/Abstract])

(sport[Title/Abstract]) AND (discrete choice[Title/Abstract])

(sport[Title/Abstract]) AND (conjoint analysis[Title/Abstract])

(empowerment[Title/Abstract]) AND (discrete choice[Title/Abstract])

(empowerment[Title/Abstract]) AND (conjoint analysis[Title/Abstract])

(exercise[Title/Abstract]) AND (discrete choice[Title/Abstract])

(exercise[Title/Abstract]) AND (conjoint analysis[Title/Abstract])

The two search phrases in gray did not lead to any result.

For peer review only

For peer review only - http://bmjopen.bmj.com/site/about/guidelines.xhtml

Flowchart of the retrieved studies and screening process

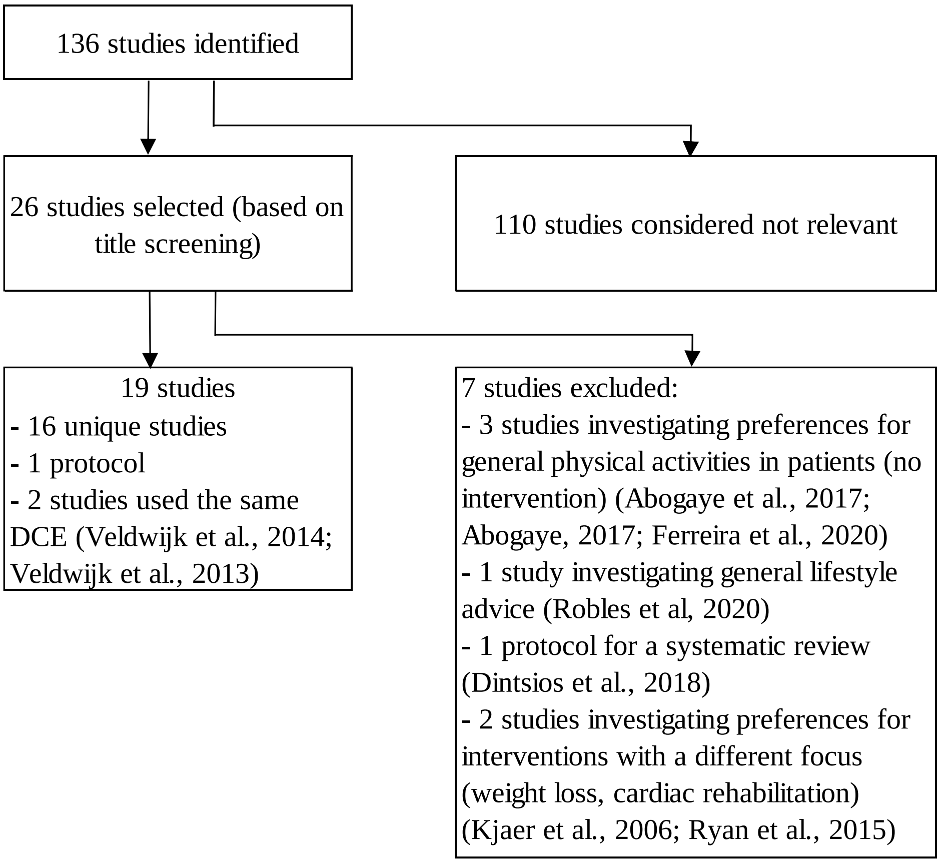

General overview of the retrieved studies

The search led to 18 studies and one protocol of one of the studies (Pinto et al., 2017). Two studies (Veldwijk et al., 2013; Veldwijk et al., 2014) used the same DCE, so we will refer to only one of them for simplicity. The studies focused on different topics but were all concerned in deploying DCE techniques to understand preferences for a physical activity intervention (sometimes labeled as "lifestyle intervention", including also diet). They dealt with the following topics:

- Lifestyle intervention for:
  - Diabetes prevention (Owen et al., 2010)
  - Stroke survivors (Geidl et al., 2018)
  - Knee osteoarthritis (Pinto et al., 2017; Pinto et al., 2019) [Study + Protocol]
  - Parkinson´s disease (Paul et al., 2021)
  - Diabetes patients (Salampessy et al., 2015; Sommer et al., 2020; Veldwijk et al., 2014, Veldwijk et al., 2013, Ramirez & Beale, 2016; van Gils et al., 2011) [two studies by Veldwijk using the same DCE]
  - Older adults (Brown, 2009)
- Focus on outcomes of health promotion interventions (Alayli-Goebbels, 2013)
- Focus on goals of health promotion interventions (Benning, 2020)
- Focus on financial incentives for physical activity (Farooqui, 2014; Giles, 2016; Molema, 2019, Wanders, 2014, Matsushita, 2017)

For peer review only - http://bmjopen.bmj.com/site/about/guidelines.xhtml

Systematic extraction of attributes and levels

We collected all attributes and levels from the selected studies. We eliminated those who were specific of the context analyzed and not transferrable to our context (diet related items, items related to a specific disease). Furthermore, we excluded those that were potentially relevant for a general physical activity intervention but were not applicable to our context (mixed groups including men, exercising alone/at home, schedule and goal setting by coach/physician, monetary interventions, goal setting, consultations with coach or physician, etc.). The remaining attributes are presented in Table A.2.

For peer review only

For peer review only - http://bmjopen.bmj.com/site/about/guidelines.xhtml

Table A.2: attributes and levels retrieved via systematic literature search

Attribute	Levels	Study
**Exercise frequency and duration**		
Exercise structure	Daily 30 min<br>daily 45 min<br>weekly 3h<br>weekly 5h	Owen et al., 2020
Frequency	1-2 sessions per week<br>3 sessions per week<br>4-5 sessions per week	Geidl et al., 2018
Duration	20-30 minutes per session<br>45-60 minutes per session	Geidl et al., 2018
Number of 45-minute exercise sessions per week	1x45-minute session per week<br>2x45-minute sessions per week<br>3x45-minute sessions per week<br>4x45-minute sessions per week	Paul et al., 2021
Total time required	12 hours/week<br>10 hours/week<br>8 hours/week<br>6 hours/week	Sommer et al., 2020
Time spent on the program	2.5 hours per week<br>4 hours per week	van Gils et al., 2011
Number of walking days per week for 3 months	2 days<br>3 days<br>5 days	Brown et al., 2009
walking time perday	20 min<br>30 min<br>45 min<br>75 min	Brown et al., 2009
time per week	0 h<br>2 h<br>4 h<br>6 h	Alayli-Goebbels et al., 201
Exercise per week	2 h<br>4 h<br>6 h	Benning et al., 2020
Average number of sessions required per week	1 session<br>2 sessions	Farooqui et al., 2014
Time per physical activity occasion	Low<br>Medium<br>High	Pinto et al., 2019

For peer review only - http://bmjopen.bmj.com/site/about/guidelines.xhtml

Attribute	Levels	Study
**Context**		
Group activities	No group activities<br>Group activities	Sommer et al., 2020
**Type of exercise**		
Type of exercise	Endurance<br>Muscular strength<br>Neuromuscular and flexibility<br>Mixed program	Geidl et al., 2018
Intensity	Light activity<br>Moderate activity<br>Vigorous activity	Geidl et al., 2018
Physical activity effort	Low<br>Medium<br>High	Pinto et al., 2019
Sports activity	Walking/cycling<br>Fitness (treadmill, rowing machine, bicycle)	van Gils et al., 2011
Exercise type	Dance program (eg, tango or waltz/foxtrot or Irish set dancing)<br>Balance exercise program (exercises to challenge balance, performed while standing, may include Tai-chi)<br>Muscle strength exercise program (exercises aimed at increasing muscle strength, eg, resistance exercises using free weights, exercises using your body weight, weight machines, weighted vests, TheraBand)<br>Aerobic exercise program (exercises to challenge the heart and lungs and make you puff, eg, fast walking, jogging, running, cycling)<br>Walking exercise program (eg, walking indoors and/or outdoors, treadmill walking, walking using visual and/or auditory cues)<br>Multimodal exercise program (including at least 2 of the following exercises): balance exercises, strength exercises, aerobic exercises, walking exercises	Paul et al., 2021
**Outcomes & goals**		
Outcomes	0.25 kg, no body change<br>0.5kg no body change<br>0.5kg body change<br>0.25 kg, body change	Owen et al., 2020
Expected outcome	No weight loss but feeling fitter 0<br>Weight loss of 5 kilograms and feeling fitter 5<br>Weight loss of 10 kilograms and feeling fitter 10	Wanders et al., 2014

For peer review only - http://bmjopen.bmj.com/site/about/guidelines.xhtml

Attribute	Levels	Study
Expected outcomes	No weights loss (reference) but you feel fitter<br>A weight loss of 5 kg and you feel fitter<br>A weight loss of 10 kg and you feel fitter	Salampessy et al., 2015
Expected outcomes	No weight loss but feeling more healthy<br>5 kilograms of weight lost and feeling more healthy<br>10 kilograms of weight lost and feeling more healthy	Veldwijk et al., 2013
expected weight loss	5 kg<br>10 kg	Benning et al., 2020
Goal	Looking better by losing weight<br>Increasing life expectancy by losing weight	Benning et al., 2020
clothing size above ideal	0 sizes<br>1/2 size<br>1 size<br>2 sizes	Alayli-Goebbels et al., 2013
Health benefits	Low<br>Medium<br>High	Pinto et al., 2019
Effect on your overall feeling of wellbeing	You will experience no improvement in overall feeling of wellbeing<br>You will experience small improvement in overall feeling of wellbeing<br>You will experience moderate improvement in overall feeling of wellbeing<br>You will experience large improvement in overall feeling of wellbeing	Paul et al., 2021
Future health state value	0.5<br>0.7<br>0.8<br>1	Alayli-Goebbels et al., 2013
start point of future health state	in 2 yrs<br>in 5 yrs<br>in 10 yrs<br>in 20 yrs	Alayli-Goebbels et al., 2013
life expectancy	80 yrs<br>81 yrs<br>82 yrs<br>83 yrs	Alayli-Goebbels et al., 2013
days without sufficient relaxation	0-1 per week<br>2-3 per week<br>4-5 per week<br>6-7 per week	Alayli-Goebbels et al., 2013

Attribute	Levels	Study
endurance	poor<br>modest<br>good<br>vey good	Alayli-Goebbels et al., 2013
experienced control over lifestyle choices	little<br>some<br>moderate<br>much	Alayli-Goebbels et al., 2013
Enjoyment	Low<br>Medium<br>High	Pinto et al., 2019
lifestyle improvemens of potential partner/children	yes<br>no	Alayli-Goebbels et al., 2013
**Schedule**		
Convenience (how well activity fits into your schedule)	Low<br>Medium<br>High	Pinto et al., 2019
**Costs**		
Enrollment fee	Free<br>$20<br>$50	Farooqui et al., 2014
Additional cost of gym/healthier food options	no additional cost<br>AU$ 15/week	Owen et al., 2020
Monthly cost	Low<br>Medium<br>High	Pinto et al., 2019
Out of pocket cost (in AU$) including travel costs per session	Out of pocket cost = $0 per session<br>Out of pocket cost = $10 per session<br>Out of pocket cost = $25 per session<br>Out of pocket cost = $50 per session<br>Out of pocket cost = $100 per session<br>Out of pocket cost = $150 per session	Paul et al., 2021
OOP costs	OOP costs of €75 per 3–6 months<br>OOP costs of €150 per 3–6 months<br>OOP costs of €225 per 3–6 months	Salampessy et al., 2015
Out-of-pocket costs	75 euro per year<br>150 euro per year<br>225 euro per year	Veldwijk et al., 2013
money per month	0 €<br>25 €<br>50 €<br>100 €	Alayli-Goebbels et al., 2013

For peer review only - http://bmjopen.bmj.com/site/about/guidelines.xhtml

Attribute	Levels	Study
Cost to travel to sessions	3 sessions<br>Free<br>$2<br>$5	Farooqui et al., 2014
**Travel time & location**		
Exercise location	In the local neighbourhood (eg, local parks, streets or outdoor public spaces)<br>At a hospital or health centre/practice<br>At a community centre or facility (eg, gym, community hall)<br>At multiple locations including home<br>At multiple locations excluding home	Paul et al., 2021
Travel time per exercise session	Less than 5 minutes each way travel time<br>5 minutes each way travel time<br>10 minutes each way travel time<br>15 minutes each way travel time<br>30 minutes each way travel time<br>60 minutes or more each way travel time	Paul et al., 2021
Time to travel to sessions	15 minutes<br>25 minutes<br>30 minutes<br>45 minutes	Farooqui et al., 2014
**Support**		
Amount of supervision	All of the exercise is supervised<br>Some of the exercise is supervised<br>None of the exercise is supervised	Paul et al., 2021
**Other**		
other information provided	no other information<br>written leaflet on benefits of activity and ways to be more active<br>face-to-face discussions on benefits of activity and ways to be more active<br>written leaflet and face-to-face idscussion on benefits of activity and ways to be more active	Giles et al., 2016

For peer review only - http://bmjopen.bmj.com/site/about/guidelines.xhtml

Appendix 3: further definition of attributes and levels based on experts feedback

In the meeting with the expert, 10 attributes were presented:

- *Price per course unit*
- *Course times*
- *Travel time to hall/course*
- *Additional social activities*
- *Additional course content*
- *Participation in organization*
- *Course registration*
- *Group composition*
- *Presence of childcare*
- *Course duration (in minutes)*

The discussion led to a reformulation of two attributes and the identification of an additional one. The additional attribute identified was "flexible intensity of the course" (i.e. if the intensity of the exercises within a course could be flexibly adjusted or not). The two attributes that were reformulated were:

- "course registration" (i.e. if all costs for the whole course must be paid in advance or if each appointment can be paid separately) was substituted by the attribute "trial lecture" (i.e. if a trial lecture is offered or not).
- "group composition" (i.e. women from the same social background/migration background vs mixed group) was substituted by an attribute specifically targeting respect of religious standards (i.e. framework: the hall is visible from the outside or not).

Based on the insights gained during the consultation with the experts, we performed a selection of those attributes which respected the following characteristics:

- Relevant for the overall utility
- Influenceable
- Close to reality (in the BIG program)
- Substitutive relation
- No dominance

Based on the insights from the experts we considered *presence of childcare* and *presence of trial lessons* as dominant criteria for the decision. These attributes, together with the presence of a female coach were considered as fix characteristics for the DCE.

According to the experts, the attribute "*course duration (in minutes)*" was distributed between 60 and 90 minutes for almost all offered courses. Given the limited variation, we decided to discard this attribute and fix it at 60 Minutes along the previously mentioned course characteristics.

The attribute "*course registration*" (paying everything at the beginning or paying each lesson at a time) was perceived as not relevant by the experts. Therefore we decided to exclude this attribute altogether.

The attribute "*flexible intensity (yes or no)*" was also deemed unimportant for the present DCE, since basically in all courses offered the intensity can be flexibly adjusted. Women also have the possibility of visiting a trial lesson and receive individual advice regarding their fitness level. This information will be included also in the fix course components.

The attribute "*framework (hall is visible from the outside yes or no)*" was deemed as a dominant criterion for some women (especially Muslim women) and not relevant for some other women. In both cases the attribute should not be included in the DCE. We therefore decided to fix this attribute as well.

The remaining attributes were included in the DCE. These are:

For peer review only - http://bmjopen.bmj.com/site/about/guidelines.xhtml

- *Price per course unit*
- *Course times*
- *Travel time to hall/course*
- *Additional activities (social + content)*
- *Participation in organization*

The attributes "*additional social activities*" and "*additional course content*" were merged into one attribute for the following reasons:

- The attribute additional course content was not clearly formulated and led experts to different interpretations/questions of what it means (e.g. additional lectures or additional content in the same appointment as the sport course? Etc.)
- At the moment in most BIG courses additional social activities include also the informal exchange on other topics (healthy diet, mental health, integration, help with translations,…) so that the two additional activities (social and content) largely overlap at the moment

The final attributes and levels included are (original language):

**Attribute**	**Levels**
**Kurszeiten**	Morgens (8-10 Uhr)<br>Vormittags (10-12 Uhr)<br>Nachmittags (16-18 Uhr)<br>Abends (18-20 Uhr)
**Dauer Geh-/Fahrweg bis zum BIG-Kurs**	Maximal 10 Minuten<br>Maximal 20 Minuten<br>Maximal 30 Minuten
**zusätzliche soziale Aktivitäten im Rahmen von BIG organisiert**	Keine zusätzliche Aktivität wird organisiert<br>Zusätzliche soziale Aktivitäten werden organisiert
**Berücksichtigung von Interessen und Wünsche bei der weiteren Kursplanung**	Interessen/Wünsche werden nicht abgefragt/nicht berücksichtigt<br>Interessen/Wünsche werden abgefragt/berücksichtigt
**Kurskosten pro Kurseinheit**	0 €<br>1 €<br>2 €<br>3 €<br>4 €<br>5 €

For peer review only - http://bmjopen.bmj.com/site/about/guidelines.xhtml

Appendix 4: list of attributes and levels in the original language (German)

Attribute	Levels
**Kurszeiten**	Morgens (8-10 Uhr)<br>Vormittags (10-12 Uhr)<br>Nachmittags (16-18 Uhr)<br>Abends (18-20 Uhr)
**Dauer Geh-/Fahrweg bis zum BIG-Kurs**	Maximal 10 Minuten<br>Maximal 20 Minuten<br>Maximal 30 Minuten
**Zusätzliche soziale Aktivitäten im Rahmen von BIG organisiert**	Keine zusätzliche Aktivität<br>Zusätzliche soziale Aktivitäten
**Berücksichtigung von Interessen und Wünsche bei der weiteren Kursplanung**	Interessen/Wünsche werden nicht berücksichtigt<br>Interesse/Wünsche werden berücksichtigt
**Kurskosten pro Kurseinheit**	2,0 €<br>3,5 €<br>5,0 €<br>6,5 €<br>8,0 €<br>9,5 €

For peer review only - http://bmjopen.bmj.com/site/about/guidelines.xhtml

References

Aboagye, E. (2017). Valuing Individuals' Preferences and Health Choices of Physical Exercise. *Pain Ther*, 6(1), 85-91. https://doi.org/10.1007/s40122-017-0067-4

Aboagye, E., Hagberg, J., Axén, I., Kwak, L., Lohela-Karlsson, M., Skillgate, E., Dahlgren, G., & Jensen, I. (2017). Individual preferences for physical exercise as secondary prevention for non-specific low back pain: A discrete choice experiment. *PLoS One*, 12(12), e0187709. https://doi.org/10.1371/journal.pone.0187709

Alayli-Goebbels, A. F., Dellaert, B. G., Knox, S. A., Ament, A. J., Lakerveld, J., Bot, S. D., Nijpels, G., & Severens, J. L. (2013). Consumer preferences for health and nonhealth outcomes of health promotion: results from a discrete choice experiment. *Value Health*, 16(1), 114-123. https://doi.org/10.1016/j.jval.2012.08.2211

Benning, T. M., Dellaert, B. G. C., & Arentze, T. A. (2020). The impact of health vs. non-health goals on individuals' lifestyle program choices: a discrete choice experiment approach. *BMC Public Health*, 20(1), 411. https://doi.org/10.1186/s12889-020-8416-3

Brown, D. S., Finkelstein, E. A., Brown, D. R., Buchner, D. M., & Johnson, F. R. (2009). Estimating older adults' preferences for walking programs via conjoint analysis. *Am J Prev Med*, 36(3), 201-207.e204. https://doi.org/10.1016/j.amepre.2008.10.014

Dintsios, C. M., Chernyak, N., Grehl, B., & Icks, A. (2018). Quantified patient preferences for lifestyle intervention programs for diabetes prevention-a protocol for a systematic review. *Syst Rev*, 7(1), 214. https://doi.org/10.1186/s13643-018-0884-5

Farooqui, M. A., Tan, Y. T., Bilger, M., & Finkelstein, E. A. (2014). Effects of financial incentives on motivating physical activity among older adults: results from a discrete choice experiment. *BMC Public Health*, 14, 141. https://doi.org/10.1186/1471-2458-14-141

Ferreira, G. E., Howard, K., Zadro, J. R., O'Keeffe, M., Lin, C. C., & Maher, C. G. (2020). People considering exercise to prevent low back pain recurrence prefer exercise programs that differ from programs known to be effective: a discrete choice experiment. *J Physiother*, 66(4), 249-255. https://doi.org/10.1016/j.jphys.2020.09.011

Geidl, W., Knocke, K., Schupp, W., & Pfeifer, K. (2018). Measuring stroke patients' exercise preferences using a discrete choice experiment. *Neurol Int*, 10(1), 6993. https://doi.org/10.4081/ni.2018.6993

Giles, E. L., Becker, F., Ternent, L., Sniehotta, F. F., McColl, E., & Adams, J. (2016). Acceptability of Financial Incentives for Health Behaviours: A Discrete Choice Experiment. *PLoS One*, 11(6), e0157403. https://doi.org/10.1371/journal.pone.0157403

Kjaer, T., Gyrd-Hansen, D., & Willaing, I. (2006). Investigating patients' preferences for cardiac rehabilitation in Denmark. *Int J Technol Assess Health Care*, 22(2), 211-218. https://doi.org/10.1017/s0266462306051038

Matsushita, M., Harada, K., & Arao, T. (2017). [Incentive program to strengthen motivation for increasing physical activity via conjoint analysis]. *Nihon Koshu Eisei Zasshi*, 64(4), 197-206. https://doi.org/10.11236/jph.64.4_197

Molema, C., Veldwijk, J., Wendel-Vos, W., de Wit, A., van de Goor, I., & Schuit, J. (2019). Chronically ill patients' preferences for a financial incentive in a lifestyle intervention. Results of a discrete choice experiment. *PLoS One*, 14(7), e0219112. https://doi.org/10.1371/journal.pone.0219112

For peer review only - http://bmjopen.bmj.com/site/about/guidelines.xhtml

Owen, K., Pettman, T., Haas, M., Viney, R., & Misan, G. (2010). Individual preferences for diet and exercise programmes: changes over a lifestyle intervention and their link with outcomes. *Public Health Nutr*, 13(2), 245-252. https://doi.org/10.1017/s1368980009990784

Paul, S. S., Canning, C. G., Löfgren, N., Sherrington, C., Lee, D. C., Bampton, J., & Howard, K. (2021). People with Parkinson's disease are more willing to do additional exercise if the exercise program has specific attributes: a discrete choice experiment. *J Physiother*, 67(1), 49-55. https://doi.org/10.1016/j.jphys.2020.12.007

Pinto, D., Bockenholt, U., Lee, J., Chang, R. W., Sharma, L., Finn, D. J., Heinemann, A. W., Holl, J. L., & Hansen, P. (2019). Preferences for physical activity: a conjoint analysis involving people with chronic knee pain. *Osteoarthritis Cartilage*, 27(2), 240-247. https://doi.org/10.1016/j.joca.2018.10.002

Pinto, D., Danilovich, M. K., Hansen, P., Finn, D. J., Chang, R. W., Holl, J. L., Heinemann, A. W., & Bockenholt, U. (2017). Qualitative Development of a Discrete Choice Experiment for Physical Activity Interventions to Improve Knee Osteoarthritis. *Arch Phys Med Rehabil*, 98(6), 1210-1216.e1211. https://doi.org/10.1016/j.apmr.2016.11.024

Ramirez, M., Wu, S., & Beale, E. (2016). Designing a Text Messaging Intervention to Improve Physical Activity Behavior Among Low-Income Latino Patients With Diabetes: A Discrete-Choice Experiment, Los Angeles, 2014-2015. *Prev Chronic Dis*, 13, E171. https://doi.org/10.5888/pcd13.160035

Robles, L. A., Wright, S. J., Hackshaw-McGeagh, L., Shingler, E., Shiridzinomwa, C., Lane, J. A., Martin, R. M., & Burden, S. (2020). Prostate cancer survivors' preferences on the delivery of diet and lifestyle advice: a pilot best-worst discrete choice experiment. *Pilot Feasibility Stud*, 6, 2. https://doi.org/10.1186/s40814-019-0549-8

Ryan, M., Yi, D., Avenell, A., Douglas, F., Aucott, L., van Teijlingen, E., & Vale, L. (2015). Gaining pounds by losing pounds: preferences for lifestyle interventions to reduce obesity. *Health Econ Policy Law*, 10(2), 161-182. https://doi.org/10.1017/s1744133114000413

Salampessy, B. H., Veldwijk, J., Jantine Schuit, A., van den Brekel-Dijkstra, K., Neslo, R. E., Ardine de Wit, G., & Lambooij, M. S. (2015). The Predictive Value of Discrete Choice Experiments in Public Health: An Exploratory Application. *Patient*, 8(6), 521-529. https://doi.org/10.1007/s40271-015-0115-2

Sommer, J., Dyczmons, J., Grobosch, S., Gontscharuk, V., Vomhof, M., Roden, M., & Icks, A. (2020). Preferences of people with type 2 diabetes for telemedical lifestyle programmes in Germany: protocol of a discrete choice experiment. *BMJ Open*, 10(9), e036995. https://doi.org/10.1136/bmjopen-2020-036995

van Gils, P. F., Lambooij, M. S., Flanderijn, M. H., van den Berg, M., de Wit, G. A., Schuit, A. J., Struijs, J. N., & van den Berg, B. (2011). Willingness to participate in a lifestyle intervention program of patients with type 2 diabetes mellitus: a conjoint analysis. *Patient Prefer Adherence*, 5, 537-546. https://doi.org/10.2147/ppa.S16854

Veldwijk, J., Lambooij, M. S., de Bekker-Grob, E. W., Smit, H. A., & de Wit, G. A. (2014). The effect of including an opt-out option in discrete choice experiments. *PLoS One*, 9(11), e111805. https://doi.org/10.1371/journal.pone.0111805

Veldwijk, J., Lambooij, M. S., van Gils, P. F., Struijs, J. N., Smit, H. A., & de Wit, G. A. (2013). Type 2 diabetes patients' preferences and willingness to pay for lifestyle programs: a discrete choice experiment. *BMC Public Health*, 13, 1099. https://doi.org/10.1186/1471-2458-13-1099

For peer review only - http://bmjopen.bmj.com/site/about/guidelines.xhtml

Wanders, J. O., Veldwijk, J., de Wit, G. A., Hart, H. E., van Gils, P. F., & Lambooij, M. S. (2014). The effect of out-of-pocket costs and financial rewards in a discrete choice experiment: an application to lifestyle programs. *BMC Public Health*, 14, 870. https://doi.org/10.1186/1471-2458-14-870

For peer review only

For peer review only - http://bmjopen.bmj.com/site/about/guidelines.xhtml

BMJ Open

Preferences of women in difficult life situations for a physical activity program: protocol of a discrete choice experiment in the German NU-BIG project

Journal:	*BMJ Open*
Manuscript ID	bmjopen-2022-067235.R1
Article Type:	Protocol
Date Submitted by the Author:	17-Mar-2023
Complete List of Authors:	Pedron, Sara; Technical University of Munich, Department of Sport and Health Sciences; Helmholtz Center Munich German Research Center for Environmental Health, Institute of Health Economics and Healthcare Management<br>Herbert-Maul, Annika; Friedrich-Alexander-Universitat Erlangen-Nurnberg, Department of Sport Science and Sport<br>Sauter, Alexandra; University of Regensburg, Department for Epidemiology and Preventive Medicine, Medical Sociology<br>Linder, Stephanie; Friedrich-Alexander-Universitat Erlangen-Nurnberg, Department of Sport Science and Sport<br>Sommer, Raluca; Friedrich-Alexander-Universitat Erlangen-Nurnberg, Department of Sport Science and Sport<br>Vomhof, Markus; Heinrich Heine University Düsseldorf; German Center for Diabetes Research<br>Gontscharuk, Veronika; Heinrich Heine University Düsseldorf; German Center for Diabetes Research<br>Abu-Omar, Karim ; Friedrich-Alexander-Universitat Erlangen-Nurnberg, Department of Sport Science and Sport<br>Thiel, Ansgar; Eberhard Karls Universitat Tubingen, Institute of Sports Science, Social and Public Health Sciences and Interfaculty Research Institute for Sport and Physical Activity<br>Ziemainz, Heiko; Friedrich-Alexander-Universitat Erlangen-Nurnberg, Department of Sport Science and Sport<br>Holle, Rolf; Ludwig-Maximilians-Universitat Munchen, Institute for Medical Informatics, Biometry and Epidemiology<br>Laxy, Michael; Technical University of Munich, Department of Sport and Health Sciences; Helmholtz Center Munich German Research Center for Environmental Health, Institute of Health Economics and Health Care Management
**Primary Subject Heading**:	Public health
Secondary Subject Heading:	Health economics, Sports and exercise medicine
Keywords:	HEALTH ECONOMICS, SPORTS MEDICINE, SOCIAL MEDICINE

Preferences of women in difficult life situations for a physical activity program: protocol of a discrete choice experiment in the German NU-BIG project

Sara Pedron [a,b,c], Annika Herbert-Maul [d], Alexandra Sauter [e], Stephanie Linder [d], Raluca Sommer [d], Markus Vomhof [f,c,g], Veronika Gontscharuk [f,c,g], Karim Abu-Omar [d], Ansgar Thiel [h], Heiko Ziemainz [d], Rolf Holle [h] & Michael Laxy [a,b,c]*

[a] Professorship of Public Health and Prevention, Department of Sport and Health Sciences, Technical University of Munich, Germany
[b] Institute of Health Economics and Health Care Management, Helmholtz Center Munich, German Research Center for Environmental Health (GmbH), Germany
[c] German Center for Diabetes Research, Neuherberg, Germany
[d] Department of Sport Science and Sport, Friedrich-Alexander-Universität Erlangen-Nuremberg, Erlangen, Germany
[e] Department for Epidemiology and Preventive Medicine, Medical Sociology, University of Regensburg, Regensburg, Germany
[f] Institute for Health Services Research and Health Economics, Centre for Health and Society, Medical Faculty and University Hospital Düsseldorf, Heinrich-Heine-University Düsseldorf, Duesseldorf, Germany.
[g] Institute for Health Services Research and Health Economics, German Diabetes Center (DDZ), Leibniz Center for Diabetes Research at the Heinrich-Heine-University Düsseldorf, Duesseldorf, Germany
[h] Institute of Sports Science, Social and Public Health Sciences and Interfaculty Research Institute for Sport and Physical Activity, Eberhard Karls University Tübingen, Tübingen, Germany
[h] Institute for Medical Informatics, Biometry and Epidemiology, University Hospital, Ludwig-Maximilian-University Munich, Munich, Germany

Sara Pedron (sara.pedron@tum.de)
Annika Herbert-Maul (annika.herbert-maul@fau.de)
Alexandra Sauter (alexandra.sauter@klinik.uni-regensburg.de)
Stephanie Linder (stephanie.linder@fau.de)
Raluca Sommer (raluca.sommer@fau.de)
Markus Vomhof (Markus.Vomhof@uni-duesseldorf.de)
Veronika Gontscharuk (Veronika.Gontscharuk@ddz.de)
Karim Abu-Omar (karim.abu-omar@fau.de)
Ansgar Thiel (ansgar.thiel@uni-tuebingen.de)
Heiko Ziemainz (heiko.ziemainz@fau.de)
Rolf Holle (rholle@ibe.med.uni-muenchen.de)
Michael Laxy (Michael.laxy@tum.de)

*Corresponding author:
Sara Pedron, PhD
Technical University of Munich, Professorship of Public Health and Prevention, Georg-Brauchle-Ring 60, 80992 München, Germany
Sara.pedron@tum.de
+49 (0) 89/289 24979

Word count: 4667

For peer review only - http://bmjopen.bmj.com/site/about/guidelines.xhtml

Abstract

Introduction: The BIG project ("Bewegung als Investition in die Gesundheit", i.e. „Movement as Investment in Health") was developed in 2005 as a community-based participatory research program to offer accessible opportunities for physical activity to women in difficult life situations. Since then, the program has been expanded to eight sites in Germany. A systematic evaluation of BIG is currently being conducted. As part of this effort, we strive to understand the preferences of participating women for different aspects of the program, and to analyze their willingness to pay.

Methods and analysis: In this protocol we describe the development and analysis plan of a discrete choice experiment (DCE) to investigate participants´ preferences for a physical activity program for women in difficult life situations. The experiment will be embedded in a questionnaire covering several aspects of participation to the program (e.g., reach, efficacy, further effects) and socioeconomic characteristics of all active participants. After a thorough search of the literature, BIG documents review and expert interviews, we identified five important attributes of the program: course times, travel time to the course venue, additional social activities organized by BIG, consideration of wishes and interests for the further planning of courses, costs per course unit. Thereafter, we piloted the experiment with a sample of participants from the target group. After data collection, the experiment will be analyzed using a conditional logit model and a latent class analysis to assess eventual heterogeneity in preferences.

Ethics and dissemination: Understanding women's preferences will provide useful insights for the further development of the program and ultimately increase participation and retention. The questionnaire, the included DCE and the pretest on participants received ethical approval (Application Nr. 20-247_1-B). We plan to disseminate the results of the DCE in peer-reviewed journals, national conferences and among participants and program coordinators and organizers.

Keywords: discrete choice experiment, disadvantaged groups, physical activity, preferences, willingness to pay

For peer review only - http://bmjopen.bmj.com/site/about/guidelines.xhtml

Strengths and limitations

- This DCE is the first one to evaluate participants` preferences for cooperative planning and social inclusion aspects of a community-based participatory research program in the physical activity promotion field.
- This DCE is the first one targeting preferences for physical activity opportunities of a vulnerable group, namely women in difficult life situations.
- This DCE necessarily represents a simplification of the decision-making process of women participating in the program, where several other aspects might play a role in driving women´s decisions to keep participating.
- Since the target group consists of women who already participate in the program, we will analyze preferences for a sustained participation, but will not be able to evaluate preferences for starting anew in the offered physical activity courses.

For peer review only

1. Introduction

Several studies have demonstrated that leisure time physical activity follows a socioeconomic gradient: individuals with a lower education, lower income and lower occupational position were consistently shown to engage less frequently in physical activity than their higher socioeconomic status (SES) counterparts [1–3]. In fact, socioeconomic factors are one of the major determinants of cardiovascular and metabolic conditions, which are among the most important drivers of morbidity and mortality worldwide [4]. The effect of socioeconomic factors on cardio-metabolic health is multifaceted, but runs mostly via physiological, psychosocial and behavioral factors, with varying degrees of influence in different contexts and life-stages [5]. Furthermore, several reviews from different contexts have shown a gender gap in physical activity, demonstrating that women are more at risk of sedentary behavior than men because of several barriers, such as family responsibilities, safety concerns or lack of culturally appropriate sport opportunities [6–10]. Therefore, the intersection of socioeconomic factors and sex specific factors makes women from disadvantaged socioeconomic backgrounds a vulnerable group at serious risk of sedentary behavior.

The fact that women from disadvantaged socioeconomic backgrounds are especially at risk for physical inactivity was demonstrated also in the German context [11–14]. Therefore, policies and programs with the aim of reducing barriers to participation (economic, time-related, cultural and social) and increasing the amount of leisure time physical activity, especially in socially disadvantaged populations, are needed. While the majority of programs involve individual cognitive-behavioral approaches to enhance physical activity [15], it has been argued that a broader approach targeting also structural aspects might be necessary to effectively improve lifestyle [16]. This is especially the case if such programs manage to activate already existing assets in the communities and increase the offer of targeted possibilities, for example by directly considering and overcoming actual and perceived barriers of the targeted groups [17].

One example of such programs from the German context is the "BIG" project ("Bewegung als Investition in Gesundheit" – "Movement as Investment in Health"). The program is based on a community-based participatory research approach, i.e. a research paradigm that aims primarily at a closer communication and involvement of the communities object of the investigation, to shape, manage and evaluate the intervention or the context studied [18]. In this case, the program targets specifically physical activity courses for women in difficult life situations, e.g. having a low household income, a migration background, are unemployed, rely on welfare aid, or are single-raising mothers [19, 20]. The program has since expanded to other twenty communities (eight of them are still active). In each one of these, a "cooperative planning

For peer review only - http://bmjopen.bmj.com/site/about/guidelines.xhtml

approach" was adopted, where women from the target group were explicitly and regularly included in the planning and organization of the courses, with the aim of overcoming barriers, increase participation and retention [21–26].

Even though the project strives to include women from the target group in the planning and organizational phase, little is known about their preferences. Understanding and evaluating the preferences of participating women is important in order to develop the program further, make it more diversified to reach specific groups of the population, and encourage participation over a longer period of time. In order to elicit patient or participant preferences, economic stated-preferences methods such as discrete choice experiments (DCE) have been increasingly used in healthcare contexts [27]. This preference elicitation method relies on the economic theory of demand [28] and on random utility theory [29, 30]. According to these theories, when confronted with a choice, individuals will choose the option with the highest level of utility, i.e. the option whose characteristics combined generate the highest utility value for the individual. DCE are a choice-based method where individuals are confronted with a series of hypothetical choice scenarios to simulate realistic choices. Respondents have to trade-off one option with all its characteristics with a second option, finally indicating which one of the two they prefer. By evaluating the final choice pattern, the method allows the computation of the relative importance of attributes. If an attribute representing costs is also assessed, then the willingness to pay (WTP) for levels can be elicited, i.e. how much a participant on average is willing to pay for an improvement in one attribute [31].

Aims of this protocol and of the DCE

Using a DCE, we aim to understand and quantify the preferences of participating women for specific structural characteristics of the BIG program and to investigate how they differ with respect to socio-demographic and cultural backgrounds. The aim of the present protocol is to line out the preparatory steps for the DCE and to present the final version of the experiment used in the questionnaire. This DCE is the first one to evaluate the cooperative planning aspect and social activities component, as structural characteristics of a community-based participatory research program in the physical activity promotion field. Investigating preferences for the BIG physical activity courses will help to deduce possible reasons of women from disadvantaged social backgrounds for participation, using a tool that mimics real life choices including a limited offer of alternatives and potential tradeoffs. Furthermore, the findings will also help to further develop the program in the active BIG sites and help to achieve a greater involvement of participating women by aligning the programs to preferences and thus

For peer review only - http://bmjopen.bmj.com/site/about/guidelines.xhtml

fostering their integration and the benefits of women from participation. Finally, identifying components that are essential or highly valuated for the target group can be useful to inform policymakers to design better strategies for physical activity promotion for women in difficult life situations.

2. Methods and Analysis

In this section we explain the background and context in which the DCE is embedded (2.1 and 2.2), the preparatory steps that were carried out to setup the DCE (2.3), the planned analyses (2.4) and patient involvement activities (2.5).

2.1 The BIG project

The BIG project ("Bewegung als Investition in Gesundheit", i.e. "Movement as Investment in Health") was started in 2005 by the Friedrich-Alexander Universität Erlangen-Nürnberg (FAU), Germany. The project specifically strives to increase physical activity among women in difficult life situations [19]. Since its start, the program has been transferred to other twenty communities/municipalities, mostly but not only in Bavaria (Germany) [21]. Currently the program is actively implemented at eight sites, with three sites in the starting phase. In the remaining nine sites the program was stopped because of different implementation and sustainability issues in the local communities.

The target group of the BIG programs are women in difficult life situations, which include women with a low income, a migration background, unemployed women, women relying on welfare aids or single mothers. However, BIG courses are open to all women who decide to participate.

Using a "cooperative planning approach" [22–25], the program aims at involving women from the target group also in the planning, organization and evaluation stages of the project. This way, highly tailored exercise programs could be constructed by eliminating barriers (e.g. by offering childcare possibilities during the course, by offering courses which respect religious norms, etc.). Ultimately, the program aims at maximizing participation and inclusion of women, thereby achieving a broader empowerment, with potential effects on self-efficacy and other health behaviors (e.g. healthy diet and smoking). Furthermore, this approach facilitates the inclusion of other women from their social network with a similar socioeconomic background, fostering integration and social exchange within the program itself.

At each site, the program offers several courses, both in gyms/halls (fitness courses) and in swimming pools (water courses). The fitness courses include for example Pilates, yoga, and

For peer review only - http://bmjopen.bmj.com/site/about/guidelines.xhtml

aerobics. The water courses include water-fitness and swimming courses. Furthermore, at some sites, additional social and cultural activities are organized, such as a “breakfast for women” (“Frauenfrühstück”) several times per year, cook-ins evenings with healthy dishes from different cultures and further occasions for mutual exchange. Given the participatory nature of the program and the active role that women have in shaping its contents and forms, the offered courses and additional activities differ from site to site (e.g. workshops for conflict management, stress reduction or self-defense) [21]. A detailed overview of the types of courses offered in each active site in the last year before the pandemic (2019), the number of participants for each course and the additional activities can be found in Appendix 1.

For the success of the program, women from the target group should be reached by these offers and should agree to participate. Furthermore, another very important aspect is their continued participation, since only a sustained participation leads to health improvements. Therefore, understanding the factors that drive their sustained participation (for example, via a DCE) is of paramount importance.

2.2 The NU-BIG evaluation and the target group

The present DCE is part of a general effort of evaluating the impact of the BIG program, called the NU-BIG study (“Nachuntersuchung des BIG-Projekts”), funded by the German Federal Ministry of Education and Research. The evaluation will follow the RE-AIM framework and was described in detail in the study protocol for the NU-BIG project [32]. The evaluation focuses on investigating the long-term effects of BIG on health behavior, inclusion and social participation, as well as factors facilitating long-term implementation and transfer. The study consists of a cross-sectional evaluation using a mixed-methods approach, including questionnaires for participating women, qualitative interviews for local organizers and focus groups with participating women.

The present DCE will be part of the questionnaire, which will be distributed to all women who participate in BIG courses between May 2022 and winter 2022/2023 in active sites (excluding sites in the starting phase). Based on pre-pandemic participation rates, it was estimated that the study population will amount to around 800 participating women at eight sites. The questionnaire will be administered as “paper and pen” survey, by handing the paper questionnaire to the target group in person during the BIG courses. Due to the diversity of BIG-activities at each site, an adjustment of the mode of administration will be required for several courses. In consultation with the trainers of each course, we will adapt the way we distribute the questionnaire to the specific requirements of each course (e.g., outdoor courses, swimming

For peer review only - http://bmjopen.bmj.com/site/about/guidelines.xhtml

classes) and to the specific linguistic background of participating women by making the questionnaire available in German, Arabic, English, Russian and Turkish.

2.3 Development of the DCE to assess participant preferences

To derive preferences it is important to carefully develop and the transfer the DCE into an experimental design following methodological standards [29, 33]. The DCE is a type of conjoint analysis, i.e. a stated-preference method that involves an indirect comparison of choices, for example via ranking, rating or choice designs, to evaluate and quantify preferences for several attributes of an intervention [33]. Therefore, designing a DCE involves a thorough identification of attributes and levels that define the intervention, and the construction of choice sets within the experimental design. In the next paragraphs, we describe this process in detail in the context of the NU-BIG study.

2.3.1 Problem and target group definition

The main aim of the DCE is understanding which aspects of the program favor a sustained participation to the program and help keeping participating women interested in new courses. The target group consists of all women who are actively participating in BIG courses, since the questionnaire will be handed in during classes starting from the month of May 2022 until the winter 2022/2023.

2.3.2 Identification of attributes and levels and transfer to the experimental design

The DCE will be embedded in the questionnaire and will present women with a series of hypothetical comparisons between two physical activity courses with predefined characteristics, asking them to choose which option they like the most.

In a first step, all program characteristics (attributes) that might influence individual preferences for participation in BIG courses should be identified. At the same time, appropriate levels of these attributes should be determined. All included attributes should be relevant for the choice of the course, should be easily influenced and close to reality, should have a substitutive relation and should not be dominant [34]. To identify attributes and their relative levels we followed a systematic approach. We describe all steps below.

Compilation of evidence (literature search)

The literature was searched for physical activity programs/interventions that were evaluated using a DCE. The full search strategy, flow chart and results can be browsed in Appendix 2.

For peer review only - http://bmjopen.bmj.com/site/about/guidelines.xhtml

We identified 19 studies, of which we systematically retrieved attributes and corresponding levels. We divided these attributes in meaningful groups: exercise frequency and duration, context, type of exercise, outcomes and goals, schedule, costs, travel time and location, support and other. It is important to note that no study specifically included exclusively women as a target group.

Compilation of evidence (program specific)

We reviewed previous documents of the BIG program (qualitative and quantitative data, protocol of the kick-off meeting) to identify potential attributes and levels in addition to those identified by the literature search. Each document was searched independently from two researchers (from the group AS, SL, AHM, SP). Resulting attributes and levels were then pooled, discussed by the group (SP, AHM, SL, AS) and compared with the results from the systematic literature search (full list available in Appendix 2).

In a further step, we selected only attributes that are applicable in the context of the BIG courses and that are already implemented or potentially implementable in the program. For example, attributes and levels which included mixed classes with men or individual courses were excluded since BIG offers only group courses for women. Furthermore, attributes involving the presence of a personal coach were also excluded since this is not intended nor feasible in BIG. Additionally, attributes referring to programs targeting specific illnesses were also excluded.

This selection led to a list of ten potentially important attributes (Appendix 3), which served as basis for discussion with the experts in the following step.

Consultation of experts

This step involved an online discussion of the previously identified list of ten attributes and their respective levels with five "BIG experts". They are BIG course instructors, organizers or long-term participants, who actively took part in shaping BIG courses at their site and who are routinely interviewed and involved in the planning phases of the NU-BIG evaluation. The expert group of women was created at the beginning of the NU-BIG evaluation to implement the grounding principles of community-based participatory research and includes highly motivated women that are active in their sites in different roles. They receive a small compensation for their participation and are involved in multiple projects within the NU-BIG evaluation.

In the meeting, which was headed by SP, AHM and AS, we explained to the experts the goals of the DCE and we presented them with a first example of trade-off decision within a fictitious

For peer review only - http://bmjopen.bmj.com/site/about/guidelines.xhtml

choice set. After this, each attribute and the respective level was thoroughly discussed in the group. Thereby we specifically asked if the ten reported characteristics are relevant ones and whether they represent important drivers in the decision to participate . Furthermore, we discussed their clarity and understandability and we explicitly asked if some important aspects might be missing. At the end of the meeting, we also asked the five experts to rank the attributes based on the perceived relevance for participating women with an interactive tool to spark further discussion.

The discussion led to interesting insights in the reasons for participating, from the point of view of the course instructors and organizers. Following this discussion, one additional attribute was identified as important one ("flexible intensity of the course") and two others were re-framed in a different way. We report the results of this discussion, including the new list of attributes and levels, in Appendix 3.

Based on these insights, the list of eleven attributes was further analyzed by researchers (SP, AHM, MV). In our screening of the attributes, we ensured that all attributes were relevant for the choice of a course and were easily influenced within the BIG program. Furthermore, we also ensured that they were in a substitutive relation (i.e. the worsening of one attribute can be compensated by an improvement in another attribute) and were not dominant (i.e. no attribute should be a necessary criterion for or against participation) [34]. The detailed decision steps and the resulting list of attributes and levels are reported in Appendix 3. The resulting list includes five attributes, i.e. (1) course time, (2) travel time to the BIG course, (3) additional activities organized by BIG, (4) consideration of wishes or interests and (5) costs per course unit. Based on the discussion with the experts, we decided to keep some attributes fixed, i.e. to introduce an initial statement clarifying that all courses would have specific characteristics. These include the presence of childcare, the presence of trial lessons, course duration, and the framework (e.g. hall is not visible from the outside). These fixed attributes are in fact usual component of all BIG courses that are already implemented to reduce barriers to participation.

Pre-test with participants

Based on the selected attributes and levels, a preliminary experimental design was conducted to prepare a full DCE for a pre-test. We tested the full DCE questionnaire to a set of voluntary participants from the target group. To this aim, we recruited participating women and performed semi-structured qualitative interviews using the think aloud technique based on a topic guide until a saturation of new content was achieved [35, 36]. This led us to include six women from two BIG sites (Erlangen and Nürnberg). The interviews were carried out by telephone and were

For peer review only - http://bmjopen.bmj.com/site/about/guidelines.xhtml

recorded after asking for consent of the participants. The interviewee was asked to read a paper version of the questionnaire that was previously sent by mail, and loudly report her thoughts and choices. The notes and recordings of the phone call were then revised by the interviewer (SP) and by a second researcher (MV), in order to identify the main problems and improvements necessary.

We are aware that both samples selected for the expert interviews and the pre-test might not be representative of the targeted population of participating women. However, it was impossible for the study team to directly access participants from the study population (the study was planned before late spring 2022, when only selected courses were taking place due to Covid-19 restrictions). The potential bias induced by this issue will be considered in the final evaluation.

In the pre-test with participants, we particularly paid attention to the following criteria:

(1) Are all the most important attributes included in the DCE?

(2) Is one or more of the included attributes dominant?

(3) Are all levels appropriate?

(4) Are the task and the questionnaire understandable and feasible?

For peer review only

Table 1: Attributes and levels included

Attributes	Levels
**Course time**	Early morning (8-10 am)
	Morning (10 am -12 pm)
	Afternoon (4-6 pm)
	Evening (6-8 pm)
**Travel time to the BIG course**	Maximum 10 Minutes
	Maximum 20 Minutes
	Maximum 30 Minutes
**Additional activities by BIG**	No additional social activity is organized
	Additional social activities are organized
**Consideration of wishes and interests for the further planning of courses**	Interests/Wishes are not asked/considered
	Interests/Wishes are asked/considered
**Costs per course unit**	2,00 €
	3,50 €
	5,00 €
	6,50 €
	8,00 €
	9,50 €

Notes: the original version in the German language is available in Appendix 4.

The interviews followed a pre-specified outline and were recorded for quality assurance and analyzed using content analysis methods by two researchers (SP and MV) [37]. This step led to a general confirmation of the understandability of the experiment among women with different backgrounds, linguistic skills and from different cities. The task was understood properly and all women engaged in the tradeoffs proposed in the DCE with ease. Drawing from the feedback of participating women, we made some adjustments in missing context information (e.g., the courses take place only during the week, the number of appointments was specified). Furthermore, we modified the range of costs per training unit proposed in the DCE. Initially we considered a price range between 0 € and 5 € per training unit, with 1 € intervals (the levels were: 0, 1, 2, 3, 4, 5 €). However, the price was perceived as an irrelevant attribute by the participants, since even the highest level was considered very affordable and comparable with the levels below. Based on specific indications of participating women on the price of courses that they currently participate in (up to 8€ per course unit), we decided to increase the price range up to 9,5 €. Furthermore, we received mixed feedbacks regarding including the level 0 €:

For peer review only - http://bmjopen.bmj.com/site/about/guidelines.xhtml

while some women perceived this as very advantageous, some others interpreted this as a sign of bad quality. Since we assume linearity in the cost attribute, we decided to eliminate this level. The final cost levels thus range between 2 € and 9,5 €, with 1,5 € intervals.

Final attributes and levels included

The final list of attributes and levels included in the DCE is reported in Table 1 (original language version in Appendix 4). We included 5 attributes, with varying number of levels. The first attribute is the course time, i.e. when the course takes place during the day. For this attribute we considered four levels: early morning, morning, afternoon and evening. The second attribute represents the travel time needed to reach the course location. This encompasses three levels: maximum 10, 20 or 30 minutes. The third attribute represents the possibility of organized social activities within the BIG course, such as a "women´s breakfast" or joint dinners where all participants of the course are invited to participate to socialize beyond the course. This attribute has two levels: activities are organized or not. The fourth attribute considers the possibility of listening to participants´ interests and wishes, to be considered in the further planning of courses. Also, this attribute is a binary one, either interests/wishes are considered or not. The final attribute is the costs of a course unit, ranging from 2 € to 9,5 € in 1,5 € intervals. Besides a clear preference for lower costs per course unit, a priori we cannot hypothesize which levels will be preferred by women.

2.3.3 DCE questionnaire design and construction of choice sets

We constructed the final DCE experimental design following best practice guidelines [38]. The number of attributes and levels included in our DCE would generate 288 potential combinations (2^2 x 3 x 4 x 6). The attribute costs per course unit entered were considered as a continuous variable in the experimental design. Due to a decentralized administration of questionnaires, we were not able to implement a block design. Since the number of possible choice scenarios is very large, we used a D-efficient design to select the most efficient combination of choice sets, while at the same time allowing the estimation of all the main effects [27, 39].

We employed SAS macros (SAS V.9.4) by Kuhfeld 2010 [40] to derive the optimal number of choice sets. The final efficient factorial fractional design (D-Efficiency=5.8409, Relative D-Efficiency=73.0117, D-error=0.1712) consisted of eight unique choice tasks.

All attributes and level combinations were checked for plausibility. In order to test reliability of the choices, we repeated one item (choice set 4), leading to nine choice sets included in the

For peer review only - http://bmjopen.bmj.com/site/about/guidelines.xhtml

final DCE. Participants are asked to choose between course A and course B. To avoid increasing the cognitive effort for participants, we did not include any opt-out option.

2.3.4 The DCE questionnaire

The DCE will be included in the NU-BIG paper-based questionnaire, after a general questionnaire introduction and questions regarding health behavior, health status, self-efficacy and integration and sociodemographic aspects. The DCE includes one page of detailed information on the content of the DCE. Participants are asked to choose out of two options the one they prefer most, i.e. the one they would less likely reject. An example of choice set is presented in Fig.1, the full questionnaire in the original language is available in Appendix 5.

*** Insert Fig. 1 about here ***

2.4 Data analysis

2.4.1 Planned analyses

The included population will be described with respect to their sociodemographic characteristics drawing from the information collected in the NU-BIG questionnaire.

Respondents with missing information in more than half of the choice sets (> 4) will be excluded from the analysis. All other women will be eligible for the analysis.

In order to estimate the preference weight of each level within each attribute we will analyze the DCE data using a conditional logit model. Thereby we will calculate the relative importance of attributes, using an effects-coding procedure [41]. In a further step, we will express the preference weights as money equivalents (i.e. as the marginal rate of substitution between the attributes and the cost attribute), in order to compute the marginal willingness to pay for a change in the level of the attributes [31].

The target population of women who participates in BIG courses is likely to be a heterogeneous one, especially with regard to sociodemographic characteristics, which might impact preferences (e.g. presence of children in the household, migration background and linguistic barriers, religious affiliation etc.). First, we will describe how the surveyed population (i.e. women who participate in BIG courses between summer 2022 and winter 2022/23) coincides with the intended target population of women in difficult life situations targeted by the BIG program. Secondly, we will carry out a latent class analysis in order to explore the underlying heterogeneity and identify classes of women with different preferences. We will also analyze whether these classes will be different according to age, employment status, education, income,

For peer review only - http://bmjopen.bmj.com/site/about/guidelines.xhtml

number of children, marital status, health status, migration status and religious affiliation (see Table 2 for details) [31]. All analyses will be conducted using R-studio[42].

Table 2: socio-demographic variables used in the latent class analysis

Socio-demographic characteristics	Measurement	Type of variable	Range/Categories
age	self-reported	continuous	unknown
employment status	self-reported	categorical	employed, retired, unemployed, other
education (highest qualification)	self-reported	categorical	no school diploma, mandatory school diploma, middle school diploma, high school diploma
education (school years)	self-reported	continuous	unknown
household monthly income	self-reported	categorical	below 500 €/month up to 6.000 € /month (in 500 € steps)
number of children (below 14 years of age)	self-reported	continuous	unknown
living alone/with partner	self-reported	categorical	living alone vs. with partner
health status	self-reported		1 (very good) - 5 (very bad)
migration status	self-reported	categorical	German born vs. Foreign born
religious affiliation	self-reported	categorical	evangelic church, catholic church, Islam, Judaism, Russian-orthodox church, no religious affiliation, other

2.4.2 Sample size calculation

All participating women in the active BIG sites will be offered the chance to participate to the NU-BIG survey. Therefore, we will have limited possibilities of influencing the sample size for the DCE. Nevertheless, computing the minimum required size of the sample, also taking into account different response rates, is crucial for ensuring the feasibility of the DCE.

In order to compute the minimum sample size, we used the following rule of thumb [43, 44]:

$$n > \frac{1000\, c}{t\, \times a}$$

Here n is the number of participants, c is the highest number of levels (in our case, c=4), t is the number of choice tasks (in our case, t=8) and a is the number of alternatives per choice task (in our case, a=2). In our case, this formula yields a minimum of 250 participants.

Based on participation prior to the Covid-19 pandemic, the number of participants to the BIG courses was estimated to be around 800. However, the context and participation are likely to be

For peer review only - http://bmjopen.bmj.com/site/about/guidelines.xhtml

different now, so that a conservative approach for the estimated target group is necessary. Even though assuming a 50% reduction in the sample, or in the response rate, the sample size is still higher than the minimum suggestion of 250.

2.5 Patient and public involvement

The BIG project is carried out using a "cooperative planning approach" [19], where women from the target group are involved in the planning and implementation of physical activity courses. Furthermore, in the NU-BIG project, as a comprehensive evaluation of BIG, the point of view of women from the target group was always taken into account by regularly involving the group of "BIG experts" in every step of the project for targeted feedback [21, 32].

Concretely, the idea for the DCE sparked from the meetings with the "BIG experts", as a way of formally investigating the preferences of participating women. Both the "BIG experts" and women from the target group of participants were involved in setting up of the DCE in the pre-test phases. Their helpful critical feedback was used to further develop the instrument.

3. Ethics and dissemination

The NU-BIG project was approved by the Ethics Commission of the Friedrich-Alexander-University of Erlangen-Nurnberg (Application Nr. 20-247_1-B). The DCE described in this protocol was included as part of the NU-BIG project and described in detail, including also recruitment for the pre-test with women from the target group.

All women participating in the pre-test read an extensive information sheet regarding the DCE and gave written informed consent and received a compensation (20 €). All participants to the final NU-BIG paper questionnaire will receive an information sheet and will be required to sign an informed consent form.

The DCE questionnaire and the relative data analysis will be carried out following the principles of good scientific practice [31, 38]. We will compile and attach to the final publication the checklist for conjoint analysis applications in healthcare [33]. The results will not only be disseminated in academic journals and conferences, but also among participants groups and organizing bodies, with the explicit aim of improving the courses offered by BIG in all sites.

For peer review only - http://bmjopen.bmj.com/site/about/guidelines.xhtml

Funding: This work was supported by the German Federal Ministry of Education and Research (grant no. 01EL20122D)

Author Contributions: SP and ML identified the research question. SP, MV and VG planned the described DCE in all phases. AHM and RS worked as coordinators of the whole NU-BIG project and contributed to the organization of preparatory steps of the DCE. SP, AHM, AS and SL carried out the necessary steps for the identification of potential attributes and interviewed the experts. SP and AS carried out the pre-test with participants. SP wrote a first draft of the manuscript, all authors read, commented and approved the manuscript. KAO, HZ, AT, AHM and RH were responsible for funding application and work as coordinators of the NU-BIG evaluation. They contributed with expert knowledge to the identification of the research question, the setup of the DCE and the final manuscript.

SP – Sara Pedron

ML – Michael Laxy

MV – Markus Vomhof

VG – Veronika Gontscharuk

AHM – Annika Herbert-Maul

AS – Alexandra Sauter

SL – Stephanie Linder

RS – Raluca Sommer

KAO – Karim Abu-Omar

HZ – Heiko Ziemainz

AT – Ansgar Thiel

RH – Rolf Holle

Competing interests statement: the authors declare no competing interests.

Data statement section: after publication of the main analysis paper, all data will be made available upon request and after anonymization to researchers who provide a sound analysis plan to replicate our results.

For peer review only

For peer review only - http://bmjopen.bmj.com/site/about/guidelines.xhtml

References

1. Gidlow C, Johnston LH, Crone D, Ellis N, James D. A systematic review of the relationship between socio-economic position and physical activity. Health Educ J. 2016;65:338–67.

2. Beenackers MA, Kamphuis CB, Giskes K, Brug J, Kunst AE, Burdorf A, et al. Socioeconomic inequalities in occupational, leisure-time, and transport related physical activity among European adults: A systematic review. Int J Behav Nutr Phys Act 2012 91. 2012;9:1–23.

3. Abu-Omar K, Messing S, Sarshar M, Gelius P, Ferschl S, Finger J, et al. Sociodemographic correlates of physical activity and sport among adults in Germany: 1997–2018. Ger J Exerc Sport Res. 2021;51:170–82.

4. Petrovic D, de Mestral C, Bochud M, Bartley M, Kivimäki M, Vineis P, et al. The contribution of health behaviors to socioeconomic inequalities in health: A systematic review. Prev Med (Baltim). 2018;113:15–31.

5. Ben-Shlomo Y, Kuh D. A life course approach to chronic disease epidemiology: conceptual models, empirical challenges and interdisciplinary perspectives. Int J Epidemiol. 2002;31:285–93.

6. Benjamin K, Donnelly TT. Barriers and facilitators influencing the physical activity of Arabic adults: A literature review. Avicenna. 2013;2013:8.

7. Bhatnagar P, Foster C, Shaw A. Barriers and facilitators to physical activity in second-generation British Indian women: A qualitative study. PLoS One. 2021;16:e0259248.

8. Siefken K, Schofield G, Schulenkorf N. Laefstael Jenses: An Investigation of Barriers and Facilitators for Healthy Lifestyles of Women in an Urban Pacific Island Context. J Phys Act Heal. 2014;11:30–7.

9. Frahsa A, Streber A, Wolff AR, Rütten A. Capabilities for Physical Activity by Turkish- and Russian-Speaking Immigrants Aged 65 Years and Older in Germany: A Qualitative Study. J Aging Phys Act. 2020;28:567–79.

10. Withall J, Jago R, Fox KR. Why some do but most don't. Barriers and enablers to engaging low-income groups in physical activity programmes: A mixed methods study. BMC Public Health. 2011;11:1–13.

For peer review only

11. Rütten A, Abu-Omar K, Adlwarth W, Meierjürgen R. Bewegungsarme lebensstile - Zur klassifizierung unterschiedlicher zielgruppen für eine gesundheitsförderliche körperliche aktivierung. Gesundheitswesen. 2007;69:393–400.

12. Hoebel J, Finger JD, Kuntz B, Kroll LE, Manz K, Lange C, et al. Changing educational inequalities in sporting inactivity among adults in Germany: a trend study from 2003 to 2012. BMC Public Health. 2017;17:1–10.

13. Krug S, Jordan S, Mensink GBM, Müters S, Finger J, Lampert T. Körperliche Aktivität: Ergebnisse der Studie zur Gesundheit Erwachsener in Deutschland (DEGS1). Bundesgesundheitsblatt - Gesundheitsforsch - Gesundheitsschutz. 2013;56:765–71.

14. Robert-Koch-Institut. Gesundheitliche Lage der Frauen in Deutschland. Gesundheitsberichterstattung des Bundes Gem getragen von RKI und Destatis. 2020.

15. Rütten A, Schow D, Breda J, Galea G, Kahlmeier S, Oppert JM, et al. Three types of scientific evidence to inform physical activity policy: results from a comparative scoping review. Int J Public Health. 2016;61:553–63.

16. Rutter H, Bes-Rastrollo M, De Henauw S, Lahti-Koski M, Lehtinen-Jacks S, Mullerova D, et al. Balancing Upstream and Downstream Measures to Tackle the Obesity Epidemic: A Position Statement from the European Association for the Study of Obesity. Obes Facts. 2017;10:61–3.

17. Rütten A, Abu-Omar K, Frahsa A, Morgan A. Assets for policy making in health promotion: Overcoming political barriers inhibiting women in difficult life situations to access sport facilities - ScienceDirect. Soc Sci Med. 2009;69:1667–73.

18. Leung MW, Yen IH, Minkler M. Community based participatory research: a promising approach for increasing epidemiology's relevance in the 21st century. Int J Epidemiol. 2004;33:499–506.

19. Rütten A, Röger U, Abu-Omar K, Frahsa A. Empowerment von frauen in sozial benachteiligten lebenslagen: Das BIG-projekt. Gesundheitswesen. 2008;70:742–7.

20. Röger U, Rütten A, Frahsa A, Abu-Omar K, Morgan A. Differences in individual empowerment outcomes of socially disadvantaged women: Effects of mode of participation and structural changes in a physical activity promotion program. Int J Public Health. 2011;56:465–73.

For peer review only - http://bmjopen.bmj.com/site/about/guidelines.xhtml

21. Herbert-Maul A, Abu-Omar K, Frahsa A, Streber A, Reimers AK. Transferring a Community-Based Participatory Research Project to Promote Physical Activity Among Socially Disadvantaged Women—Experiences From 15 Years of BIG. Front Public Heal. 2020;8:546.

22. Haldane V, Chuah FLH, Srivastava A, Singh SR, Koh GCH, Seng CK, et al. Community participation in health services development, implementation, and evaluation: A systematic review of empowerment, health, community, and process outcomes. PLoS One. 2019;14:e0216112.

23. O'Mara-Eves A, Brunton G, Oliver S, Kavanagh J, Jamal F, Thomas J. The effectiveness of community engagement in public health interventions for disadvantaged groups: A meta-analysis. BMC Public Health. 2015;15:1–23.

24. Rütten A, Gelius P. Building policy capacities: an interactive approach for linking knowledge to action in health promotion. Health Promot Int. 2014;29:569–82.

25. Gelius PC, Jansen M, King AC. Cooperative planning and its utilization in German physical activity promotion: a brief introduction. Health Promot Int. 2021;36 Supplement_2:ii1–7.

26. Sanz-Remacha M, García-González L, Sevil Serrano J, Aibar Solana A. A Qualitative Evaluation of a Community-Based Intervention on Health-Related Behaviors in Disadvantaged Women. Res Q Exerc Sport. 2022. https://doi.org/10.1080/02701367.2021.1971149.

27. Bekker-Grob EW de, Ryan M, Gerard K. Discrete choice experiments in health economics: a review of the literature. Health Econ. 2012;21:145–72.

28. Lancaster KJ. A New Approach to Consumer Theory. J Polit Econ. 1966;74:132–57.

29. Louviere JJ, Hensher DA, Swait JD, Adamowicz W. Stated Choice Methods: Analysis and Applications. Cambridge University Press; 2000.

30. Lancsar E, Savage E. Deriving welfare measures from discrete choice experiments: inconsistency between current methods and random utility and welfare theory. Health Econ. 2004;13:901–7.

31. Hauber AB, González JM, Groothuis-Oudshoorn CGM, Prior T, Marshall DA, Cunningham C, et al. Statistical Methods for the Analysis of Discrete Choice Experiments: A

For peer review only

For peer review only - http://bmjopen.bmj.com/site/about/guidelines.xhtml

Report of the ISPOR Conjoint Analysis Good Research Practices Task Force. Value Heal. 2016;19:300–15.

32. Abu-Omar K, Ziemainz H, Loss J, Laxy M, Holle R, Thiel A, et al. The Long-Term Public Health Impact of a Community-Based Participatory Research Project for Health Promotion Among Socially Disadvantaged Women—A Case Study Protocol. Front Public Heal. 2021;9:317.

33. Bridges JFP, Hauber AB, Marshall D, Lloyd A, Prosser LA, Regier DA, et al. Conjoint analysis applications in health - A checklist: A report of the ISPOR Good Research Practices for Conjoint Analysis Task Force. Value Heal. 2011;14:403–13.

34. Telser H. Nutzenmessung im Gesundheitswesen: Die Methode der Discrete-Choice-Experimente. Hamburg: Verlag Dr. Kovač; 2002.

35. Drennan J. Cognitive interviewing: verbal data in the design and pretesting of questionnaires. J Adv Nurs. 2003;42:57–63.

36. Charters E. The Use of Think-aloud Methods in Qualitative Research An Introduction to Think-aloud Methods. Brock Educ J. 2003;12.

37. Clarke V, Braun V. Thematic Analysis. Encycl Crit Psychol. 2014;:1947–52.

38. Johnson FR, Lancsar E, Marshall D, Kilambi V, Mühlbacher A, Regier DA, et al. Constructing Experimental Designs for Discrete-Choice Experiments: Report of the ISPOR Conjoint Analysis Experimental Design Good Research Practices Task Force. Value Heal. 2013;16:3–13.

39. Lancsar E, Louviere J. Conducting discrete choice experiments to inform healthcare decision making: A user's guide. Pharmacoeconomics. 2008;26:661–77.

40. Kuhfeld WF. Marketing Research Methods in SAS: Experimental Design, Choice, Conjoint, and Graphical Techniques. MR-2010. 2010.

41. Bech M, Gyrd-Hansen D. Effects coding in discrete choice experiments. Health Econ. 2005;14:1079–83.

42. RStudio Team. RStudio: Integrated Development for R. 2020.

43. de Bekker-Grob EW, Donkers B, Jonker MF, Stolk EA. Sample Size Requirements for Discrete-Choice Experiments in Healthcare: a Practical Guide. Patient. 2015;8:373–84.

For peer review only - http://bmjopen.bmj.com/site/about/guidelines.xhtml

44. Orme BK. Getting started with conjoint analysis : strategies for product design and pricing research. 2nd edition. Madison, WI: Research Publishers; 2010.

For peer review only

Figure legends

Fig. 1: Example of choice set

Note: own translation from German.

For peer review only

For peer review only - http://bmjopen.bmj.com/site/about/guidelines.xhtml

	Course A	Course B
**Course times**	Morning (10 am – 12 pm)	Early morning (8-10 am)
**Travel time to BIG course**	Max. 20 minutes	Max. 10 minutes
**Additional activities orgsnized by BIG**	No additional activity	Additional social activities
**Consideration of wishes and interests for the further planning of courses**	Interests/wishes are considered	Interests/wishes are not considered
**Costs per course unit**	8,0 €	5,0 €
**Which course do you prefer? (Please cross the relative box)**	☐	☐

Fig. 1: Example of choice set

318x117mm (120 x 120 DPI)

Preferences of women in difficult life situations for a physical activity program: protocol of a discrete choice experiment in the NU-BIG project

Sara Pedron, Annika Herbert-Maul, Alexandra, Stephanie Linder, Raluca Sommer, Markus Vomhof, Veronika Gontscharuk, Karim Abu-Omar, Ansgar, Heiko Ziemainz, Rolf Holle & Michael Laxy

Supplementary material

For peer review only

Appendix 1: course description in each BIG-site

Table A.1: description of courses in active sites in 2019

	time unit	Number of participants	Number of fitness courses	Number of water courses	Additional offers	Additional social activities	Planning & participation	mean number of participants per course
Erlangen	year	695	57	13	women-only bathing time, additional sport activities (dancing 2x per year, archery, climbing,…)	women´s breakfast (8x year), healthy diet course (2x year), arabic cuisine tasting (1x year)	Planning meeting (2x year)	10
Regensburg	I	166	34	3	women-only bathing time, biking course			10
	II	132						
	III	88						
Bayreuth	I	127	18	8	biking course			13
	II	112						
	III	108						
Straubing		144	10	0	boxing	integration course		14
Berlin (Treptow-Köpenick)		4	1					4
Nürnberg	I	63	0	3				21
	II	61	0	3				
	III	81	0	4				
Neustadt a.d. Aisch		20		2				10

Note: the table shows the number of participants in each active site in 2019, either for the whole years or for different course seasons (I, II or III).

For peer review only - http://bmjopen.bmj.com/site/about/guidelines.xhtml

Appendix 2: Systematic literature search protocol and results

Goal: finding studies which evaluated individual preferences for lifestyle programs/physical activity programs

Search in PubMed:

(physical activity[Title/Abstract]) AND (discrete choice[Title/Abstract])

((physical activity[Title/Abstract]) AND (conjoint analysis[Title/Abstract])

(lifestyle[Title/Abstract]) AND (discrete choice[Title/Abstract])

(coaching[Title/Abstract]) AND (discrete choice[Title/Abstract])

(coaching[Title/Abstract]) AND (conjoint analysis[Title/Abstract])

(sport[Title/Abstract]) AND (discrete choice[Title/Abstract])

(sport[Title/Abstract]) AND (conjoint analysis[Title/Abstract])

(empowerment[Title/Abstract]) AND (discrete choice[Title/Abstract])

(empowerment[Title/Abstract]) AND (conjoint analysis[Title/Abstract])

(exercise[Title/Abstract]) AND (discrete choice[Title/Abstract])

(exercise[Title/Abstract]) AND (conjoint analysis[Title/Abstract])

The two search phrases in gray did not lead to any result.

For peer review only

Flowchart of the retrieved studies and screening process

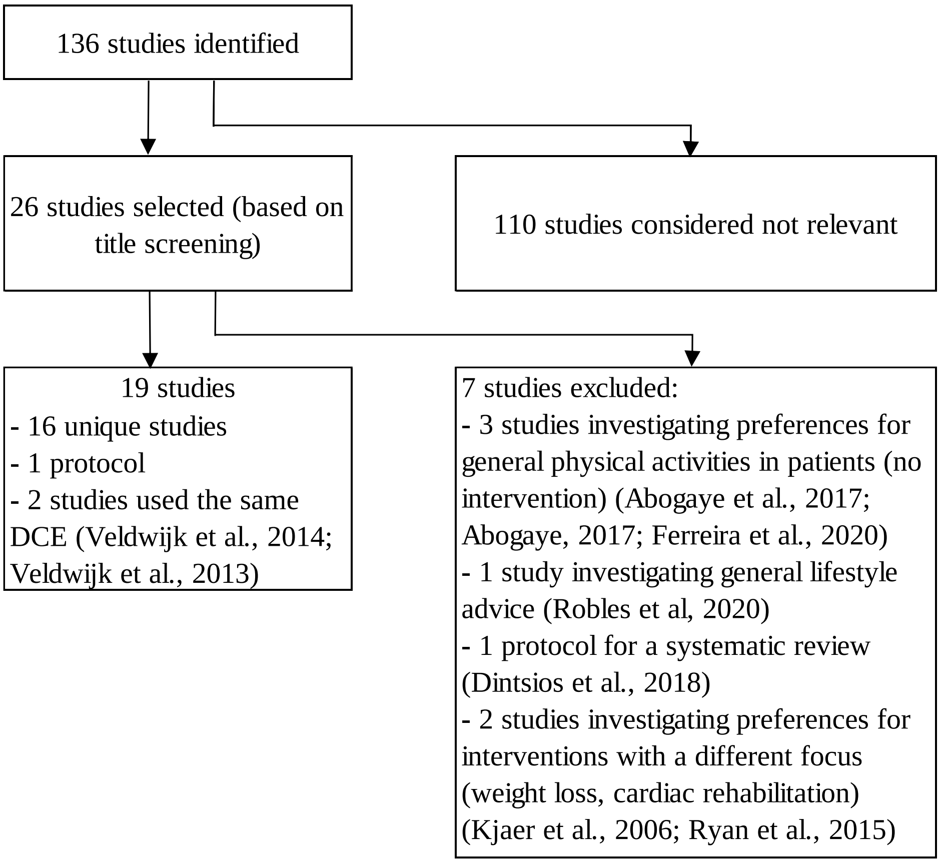

General overview of the retrieved studies

The search led to 18 studies and one protocol of one of the studies (Pinto et al., 2017). Two studies (Veldwijk et al., 2013; Veldwijk et al., 2014) used the same DCE, so we will refer to only one of them for simplicity. The studies focused on different topics but were all concerned in deploying DCE techniques to understand preferences for a physical activity intervention (sometimes labeled as “lifestyle intervention”, including also diet). They dealt with the following topics:

- Lifestyle intervention for:
  - Diabetes prevention (Owen et al., 2010)
  - Stroke survivors (Geidl et al., 2018)
  - Knee osteoarthritis (Pinto et al., 2017; Pinto et al., 2019) [Study + Protocol]
  - Parkinson´s disease (Paul et al., 2021)
  - Diabetes patients (Salampessy et al., 2015; Sommer et al., 2020; Veldwijk et al., 2014, Veldwijk et al., 2013, Ramirez & Beale, 2016; van Gils et al., 2011) [two studies by Veldwijk using the same DCE]
  - Older adults (Brown, 2009)
- Focus on outcomes of health promotion interventions (Alayli-Goebbels, 2013)
- Focus on goals of health promotion interventions (Benning, 2020)
- Focus on financial incentives for physical activity (Farooqui, 2014; Giles, 2016; Molema, 2019, Wanders, 2014, Matsushita, 2017)

For peer review only - http://bmjopen.bmj.com/site/about/guidelines.xhtml

Systematic extraction of attributes and levels

We collected all attributes and levels from the selected studies. We eliminated those who were specific of the context analyzed and not transferrable to our context (diet related items, items related to a specific disease). Furthermore, we excluded those that were potentially relevant for a general physical activity intervention but were not applicable to our context (mixed groups including men, exercising alone/at home, schedule and goal setting by coach/physician, monetary interventions, goal setting, consultations with coach or physician, etc.). The remaining attributes are presented in Table A.2.

For peer review only

Table A.2: attributes and levels retrieved via systematic literature search

Attribute	Levels	Study
**Exercise frequency and duration**		
Exercise structure	Daily 30 min<br>daily 45 min<br>weekly 3h<br>weekly 5h	Owen et al., 2020
Frequency	1-2 sessions per week<br>3 sessions per week<br>4-5 sessions per week	Geidl et al., 2018
Duration	20-30 minutes per session<br>45-60 minutes per session	Geidl et al., 2018
Number of 45-minute exercise sessions per week	1x45-minute session per week<br>2x45-minute sessions per week<br>3x45-minute sessions per week<br>4x45-minute sessions per week	Paul et al., 2021
Total time required	12 hours/week<br>10 hours/week<br>8 hours/week<br>6 hours/week	Sommer et al., 2020
Time spent on the program	2.5 hours per week<br>4 hours per week	van Gils et al., 2011
Number of walking days per week for 3 months	2 days<br>3 days<br>5 days	Brown et al., 2009
walking time perday	20 min<br>30 min<br>45 min<br>75 min	Brown et al., 2009
time per week	0 h<br>2 h<br>4 h<br>6 h	Alayli-Goebbels et al., 201
Exercise per week	2 h<br>4 h<br>6 h	Benning et al., 2020
Average number of sessions required per week	1 session<br>2 sessions	Farooqui et al., 2014
Time per physical activity occasion	Low<br>Medium<br>High	Pinto et al., 2019

For peer review only - http://bmjopen.bmj.com/site/about/guidelines.xhtml

Attribute	Levels	Study
**Context**		
Group activities	No group activities<br>Group activities	Sommer et al., 2020
**Type of exercise**		
Type of exercise	Endurance<br>Muscular strength<br>Neuromuscular and flexibility<br>Mixed program	Geidl et al., 2018
Intensity	Light activity<br>Moderate activity<br>Vigorous activity	Geidl et al., 2018
Physical activity effort	Low<br>Medium<br>High	Pinto et al., 2019
Sports activity	Walking/cycling<br>Fitness (treadmill, rowing machine, bicycle)	van Gils et al., 2011
Exercise type	Dance program (eg, tango or waltz/foxtrot or Irish set dancing)<br>Balance exercise program (exercises to challenge balance, performed while standing, may include Tai-chi)<br>Muscle strength exercise program (exercises aimed at increasing muscle strength, eg, resistance exercises using free weights, exercises using your body weight, weight machines, weighted vests, TheraBand)<br>Aerobic exercise program (exercises to challenge the heart and lungs and make you puff, eg, fast walking, jogging, running, cycling)<br>Walking exercise program (eg, walking indoors and/or outdoors, treadmill walking, walking using visual and/or auditory cues)<br>Multimodal exercise program (including at least 2 of the following exercises): balance exercises, strength exercises, aerobic exercises, walking exercises	Paul et al., 2021
**Outcomes & goals**		
Outcomes	0.25 kg, no body change<br>0.5kg no body change<br>0.5kg body change<br>0.25 kg, body change	Owen et al., 2020
Expected outcome	No weight loss but feeling fitter 0<br>Weight loss of 5 kilograms and feeling fitter 5<br>Weight loss of 10 kilograms and feeling fitter 10	Wanders et al., 2014

For peer review only - http://bmjopen.bmj.com/site/about/guidelines.xhtml

Attribute	Levels	Study
Expected outcomes	No weights loss (reference) but you feel fitter<br>A weight loss of 5 kg and you feel fitter<br>A weight loss of 10 kg and you feel fitter	Salampessy et al., 2015
Expected outcomes	No weight loss but feeling more healthy<br>5 kilograms of weight lost and feeling more healthy<br>10 kilograms of weight lost and feeling more healthy	Veldwijk et al., 2013
expected weight loss	5 kg<br>10 kg	Benning et al., 2020
Goal	Looking better by losing weight<br>Increasing life expectancy by losing weight	Benning et al., 2020
clothing size above ideal	0 sizes<br>1/2 size<br>1 size<br>2 sizes	Alayli-Goebbels et al., 2013
Health benefits	Low<br>Medium<br>High	Pinto et al., 2019
Effect on your overall feeling of wellbeing	You will experience no improvement in overall feeling of wellbeing<br>You will experience small improvement in overall feeling of wellbeing<br>You will experience moderate improvement in overall feeling of wellbeing<br>You will experience large improvement in overall feeling of wellbeing	Paul et al., 2021
Future health state value	0.5<br>0.7<br>0.8<br>1	Alayli-Goebbels et al., 2013
start point of future health state	in 2 yrs<br>in 5 yrs<br>in 10 yrs<br>in 20 yrs	Alayli-Goebbels et al., 2013
life expectancy	80 yrs<br>81 yrs<br>82 yrs<br>83 yrs	Alayli-Goebbels et al., 2013
days without sufficient relaxation	0-1 per week<br>2-3 per week<br>4-5 per week<br>6-7 per week	Alayli-Goebbels et al., 2013

Attribute	Levels	Study
endurance	poor<br>modest<br>good<br>vey good	Alayli-Goebbels et al., 2013
experienced control over lifestyle choices	little<br>some<br>moderate<br>much	Alayli-Goebbels et al., 2013
Enjoyment	Low<br>Medium<br>High	Pinto et al., 2019
lifestyle improvemens of potential partner/children	yes<br>no	Alayli-Goebbels et al., 2013
**Schedule**		
Convenience (how well activity fits into your schedule)	Low<br>Medium<br>High	Pinto et al., 2019
**Costs**		
Enrollment fee	Free<br>$20<br>$50	Farooqui et al., 2014
Additional cost of gym/healthier food options	no additional cost<br>AU$ 15/week	Owen et al., 2020
Monthly cost	Low<br>Medium<br>High	Pinto et al., 2019
Out of pocket cost (in AU$) including travel costs per session	Out of pocket cost = $0 per session<br>Out of pocket cost = $10 per session<br>Out of pocket cost = $25 per session<br>Out of pocket cost = $50 per session<br>Out of pocket cost = $100 per session<br>Out of pocket cost = $150 per session	Paul et al., 2021
OOP costs	OOP costs of €75 per 3–6 months<br>OOP costs of €150 per 3–6 months<br>OOP costs of €225 per 3–6 months	Salampessy et al., 2015
Out-of-pocket costs	75 euro per year<br>150 euro per year<br>225 euro per year	Veldwijk et al., 2013
money per month	0 €<br>25 €<br>50 €<br>100 €	Alayli-Goebbels et al., 2013

For peer review only - http://bmjopen.bmj.com/site/about/guidelines.xhtml

Attribute	Levels	Study
Cost to travel to sessions	3 sessions<br>Free<br>$2<br>$5	Farooqui et al., 2014
**Travel time & location**		
Exercise location	In the local neighbourhood (eg, local parks, streets or outdoor public spaces)<br>At a hospital or health centre/practice<br>At a community centre or facility (eg, gym, community hall)<br>At multiple locations including home<br>At multiple locations excluding home	Paul et al., 2021
Travel time per exercise session	Less than 5 minutes each way travel time<br>5 minutes each way travel time<br>10 minutes each way travel time<br>15 minutes each way travel time<br>30 minutes each way travel time<br>60 minutes or more each way travel time	Paul et al., 2021
Time to travel to sessions	15 minutes<br>25 minutes<br>30 minutes<br>45 minutes	Farooqui et al., 2014
**Support**		
Amount of supervision	All of the exercise is supervised<br>Some of the exercise is supervised<br>None of the exercise is supervised	Paul et al., 2021
**Other**		
other information provided	no other information<br>written leaflet on benefits of activity and ways to be more active<br>face-to-face discussions on benefits of activity and ways to be more active<br>written leaflet and face-to-face idscussion on benefits of activity and ways to be more active	Giles et al., 2016

For peer review only - http://bmjopen.bmj.com/site/about/guidelines.xhtml

Appendix 3: further definition of attributes and levels based on experts feedback

In the meeting with the expert, 10 attributes were presented:

- *Price per course unit*
- *Course times*
- *Travel time to hall/course*
- *Additional social activities*
- *Additional course content*
- *Participation in organization*
- *Course registration*
- *Group composition*
- *Presence of childcare*
- *Course duration (in minutes)*

The discussion led to a reformulation of two attributes and the identification of an additional one. The additional attribute identified was "flexible intensity of the course" (i.e. if the intensity of the exercises within a course could be flexibly adjusted or not). The two attributes that were reformulated were:

- "course registration" (i.e. if all costs for the whole course must be paid in advance or if each appointment can be paid separately) was substituted by the attribute "trial lecture" (i.e. if a trial lecture is offered or not).
- "group composition" (i.e. women from the same social background/migration background vs mixed group) was substituted by an attribute specifically targeting respect of religious standards (i.e. framework: the hall is visible from the outside or not).

Based on the insights gained during the consultation with the experts, we performed a selection of those attributes which respected the following characteristics:

- Relevant for the overall utility
- Influenceable
- Close to reality (in the BIG program)
- Substitutive relation
- No dominance

Based on the insights from the experts we considered *presence of childcare* and *presence of trial lessons* as dominant criteria for the decision. These attributes, together with the presence of a female coach were considered as fix characteristics for the DCE.

According to the experts, the attribute "*course duration (in minutes)*" was distributed between 60 and 90 minutes for almost all offered courses. Given the limited variation, we decided to discard this attribute and fix it at 60 Minutes along the previously mentioned course characteristics.

The attribute "*course registration*" (paying everything at the beginning or paying each lesson at a time) was perceived as not relevant by the experts. Therefore we decided to exclude this attribute altogether.

The attribute "*flexible intensity (yes or no)*" was also deemed unimportant for the present DCE, since basically in all courses offered the intensity can be flexibly adjusted. Women also have the possibility of visiting a trial lesson and receive individual advice regarding their fitness level. This information will be included also in the fix course components.

The attribute "*framework (hall is visible from the outside yes or no)*" was deemed as a dominant criterion for some women (especially Muslim women) and not relevant for some other women. In both cases the attribute should not be included in the DCE. We therefore decided to fix this attribute as well.

The remaining attributes were included in the DCE. These are:

For peer review only - http://bmjopen.bmj.com/site/about/guidelines.xhtml

- *Price per course unit*
- *Course times*
- *Travel time to hall/course*
- *Additional activities (social + content)*
- *Participation in organization*

The attributes "*additional social activities*" and "*additional course content*" were merged into one attribute for the following reasons:

- The attribute additional course content was not clearly formulated and led experts to different interpretations/questions of what it means (e.g. additional lectures or additional content in the same appointment as the sport course? Etc.)
- At the moment in most BIG courses additional social activities include also the informal exchange on other topics (healthy diet, mental health, integration, help with translations,…) so that the two additional activities (social and content) largely overlap at the moment

The final attributes and levels included are (original language):

Attribute	Levels
**Kurszeiten**	Morgens (8-10 Uhr)<br>Vormittags (10-12 Uhr)<br>Nachmittags (16-18 Uhr)<br>Abends (18-20 Uhr)
**Dauer Geh-/Fahrweg bis zum BIG-Kurs**	Maximal 10 Minuten<br>Maximal 20 Minuten<br>Maximal 30 Minuten
**zusätzliche soziale Aktivitäten im Rahmen von BIG organisiert**	Keine zusätzliche Aktivität wird organisiert<br>Zusätzliche soziale Aktivitäten werden organisiert
**Berücksichtigung von Interessen und Wünsche bei der weiteren Kursplanung**	Interessen/Wünsche werden nicht abgefragt/nicht berücksichtigt<br>Interessen/Wünsche werden abgefragt/berücksichtigt
**Kurskosten pro Kurseinheit**	0 €<br>1 €<br>2 €<br>3 €<br>4 €<br>5 €

For peer review only - http://bmjopen.bmj.com/site/about/guidelines.xhtml

Appendix 4: list of attributes and levels in the original language (German)

Attribute	Levels
**Kurszeiten**	Morgens (8-10 Uhr)
	Vormittags (10-12 Uhr)
	Nachmittags (16-18 Uhr)
	Abends (18-20 Uhr)
**Dauer Geh-/Fahrweg bis zum BIG-Kurs**	Maximal 10 Minuten
	Maximal 20 Minuten
	Maximal 30 Minuten
**Zusätzliche soziale Aktivitäten im Rahmen von BIG organisiert**	Keine zusätzliche Aktivität
	Zusätzliche soziale Aktivitäten
**Berücksichtigung von Interessen und Wünsche bei der weiteren Kursplanung**	Interessen/Wünsche werden nicht berücksichtigt
	Interesse/Wünsche werden berücksichtigt
**Kurskosten pro Kurseinheit**	2,0 €
	3,5 €
	5,0 €
	6,5 €
	8,0 €
	9,5 €

For peer review only - http://bmjopen.bmj.com/site/about/guidelines.xhtml

Appendix 5: final DCE questionnaire (original language)

For peer review only

For peer review only - http://bmjopen.bmj.com/site/about/guidelines.xhtml

BIG-Projekt

Fragebogen

Liebe Teilnehmerin,

„BIG“ steht für „Bewegung als Investition in Gesundheit“. Bei BIG werden Sport- und Bewegungskurse für Frauen angeboten. Bei diesen Kursen ist es sehr wichtig, dass jede interessierte Frau teilnehmen kann. Deswegen sollen die Kurse ihren Wünschen, Bedürfnissen und Interessen so gut wie möglich entsprechen. Vielleicht wussten Sie das noch nicht, aber auch der Kurs an dem Sie teilnehmen, ist aus dem BIG-Projekt entstanden.

Um BIG weiter zu verbessern, möchten wir gerne mehr über Ihre Wünsche und Interessen erfahren. Außerdem interessiert uns, ob BIG bei Ihnen eine Wirkung erzielt hat, z.B. auf Ihre Gesundheit. In dem Fragebogen werden deshalb Fragen zu den Kursen und Ihrer persönlichen Situation gestellt. Wir bitten Sie diese Fragen zu beantworten.

Wir bedanken uns herzlich für Ihre Unterstützung.

Ihr BIG-Team.

For peer review only

wichtige Informationen:

- Bitte beantworten Sie die Fragen, wie es Ihrer persönlichen Meinung entspricht.
- Es gibt keine richtigen oder falschen Antworten.
- Ihre Angaben sind anonym. Es sind keine Rückschlüsse auf Ihre Person möglich.
- Wir versichern Ihnen, dass Ihre Angaben vertraulich behandelt werden.
- Ihre Teilnahme ist freiwillig.
- Sollten Sie eine Frage nicht verstehen oder beantworten wollen, lassen Sie diese aus.

For peer review only - http://bmjopen.bmj.com/site/about/guidelines.xhtml

K. Kurspräferenzen

Wir möchten in diesem Teil herausfinden, welche Wünsche Sie haben, wie BIG-Sportkurse gestaltet sein sollen. Ziel ist es, Kurse anzubieten, an denen Sie auch weiterhin gerne teilnehmen.

In den folgenden Fragen werden jeweils zwei Kurse gegenübergestellt. Die Kurse unterscheiden sich in den Kurskosten, den Kurszeiten, der Dauer des Geh-/Fahrwegs, der Berücksichtigung von Interessen und zusätzlichen sozialen Aktivitäten.

Alle BIG-Sportkurse dauern ungefähr 60 Minuten und finden in Räumlichkeiten statt, die von außen nicht einsehbar sind. Sie können alle BIG-Sportkurse in einer Schnupperstunde testen, bevor Sie sich für den Kurs entscheiden. In allen BIG-Sportkursen besteht die Möglichkeit einer Kinderbetreuung. Ein Kurs hat jeweils 10 Termine. Die Termine finden unter der Woche statt.

Bitte kreuzen Sie jeweils an, ob Sie lieber an Kurs A oder Kurs B teilnehmen würden.

Sie können entweder Kurs A oder Kurs B wählen. Sie können nicht einen Kurs aus einzelnen Eigenschaften zusammenstellen. Bitte wählen Sie immer den Kurs, den Sie bevorzugen beziehungsweise den Kurs, den Sie weniger ablehnen.

Hier finden Sie eine Beschreibung der Eigenschaften, welche die zwei Kurse haben können:

Kurszeiten: Wann der Kurs stattfindet. Mögliche Uhrzeiten sind morgens (8-10 Uhr), vormittags (11-12 Uhr), nachmittags (16-18 Uhr) und abends (18-20 Uhr).

Dauer Fahrweg: Wie lange Sie zu der Halle/dem Kursort brauchen (zu Fuß oder mit dem für Sie bequemsten Verkehrsmittel): die Möglichkeiten sind 10, 20 oder 30 Minuten.

Zusätzliche soziale Aktivitäten im Rahmen von BIG organisiert: Ob zusätzliche soziale Aktivitäten von BIG organisiert werden oder nicht (z.B. Frauenfrühstück oder gemeinsame Kochabende einmal im Monat).

Berücksichtigung von Interessen und Wünsche: Ob Ihre persönlichen Interessen oder Wünsche bei der Planung von Kursen abgefragt und (wenn möglich) berücksichtigt werden oder nicht.

Kurskosten pro Termin: Wie viel Sie für jeden Kurstermin zahlen müssen (in €). In unserem Fragebogen liegt der Preis der Kurse zwischen 2,00 und 9,50 Euro pro Termin.

For peer review only - http://bmjopen.bmj.com/site/about/guidelines.xhtml

Bitte wählen Sie den Kurs aus, der Ihnen mehr zusagt. Bitte wählen Sie immer einen Kurs.

L1. Auswahlmöglichkeit	Kurs A	Kurs B
**Kurszeiten**	Vormittags (10-12 Uhr)	Morgens (8-10 Uhr)
**Dauer Geh-/Fahrweg bis zum BIG-Kurs**	Maximal 20 Minuten	Maximal 10 Minuten
**Zusätzliche soziale Aktivitäten im Rahmen von BIG organisiert**	Keine zusätzliche Aktivität	Zusätzliche soziale Aktivitäten
**Berücksichtigung von Interessen und Wünsche bei der weiteren Kursplanung**	Interessen/Wünsche werden berücksichtigt	Interessen/Wünsche werden nicht berücksichtigt
**Kurskosten (pro Termin)**	8,00 €	5,00 €
**Welchen Kurs bevorzugen Sie? (Kreuzen Sie bitte das entsprechende Kästchen an)**	☐	☐

Bitte wählen Sie den Kurs aus, der Ihnen mehr zusagt. Bitte wählen Sie immer einen Kurs.

L2. Auswahlmöglichkeit	Kurs A	Kurs B
**Kurszeiten**	Abends (18-20 Uhr)	Nachmittags (16-18 Uhr)
**Dauer Geh-/Fahrweg bis zum BIG-Kurs**	Maximal 30 Minuten	Maximal 20 Minuten
**Zusätzliche soziale Aktivitäten im Rahmen von BIG organisiert**	Keine zusätzliche Aktivität	Zusätzliche soziale Aktivitäten
**Berücksichtigung von Interessen und Wünsche bei der weiteren Kursplanung**	Interessen/Wünsche werden berücksichtigt	Interessen/Wünsche werden nicht berücksichtigt
**Kurskosten (pro Termin)**	5,00 €	9,50 €
**Welchen Kurs bevorzugen Sie? (Kreuzen Sie bitte das entsprechende Kästchen an)**	☐	☐

Bitte wählen Sie den Kurs aus, der Ihnen mehr zusagt. Bitte wählen Sie immer einen Kurs.

L3. Auswahlmöglichkeit	Kurs A	Kurs B
**Kurszeiten**	Abends (18-20 Uhr)	Vormittags (10-12 Uhr)
**Dauer Geh-/Fahrweg bis zum BIG-Kurs**	Maximal 20 Minuten	Maximal 10 Minuten
**Zusätzliche soziale Aktivitäten im Rahmen von BIG organisiert**	Keine zusätzliche Aktivität	Zusätzliche soziale Aktivitäten
**Berücksichtigung von Interessen und Wünsche bei der weiteren Kursplanung**	Interessen/Wünsche werden nicht berücksichtigt	Interessen/Wünsche werden berücksichtigt
**Kurskosten (pro Termin)**	3,50 €	9,50 €
**Welchen Kurs bevorzugen Sie? (Kreuzen Sie bitte das entsprechende Kästchen an)**	☐	☐

For peer review only - http://bmjopen.bmj.com/site/about/guidelines.xhtml

Bitte wählen Sie den Kurs aus, der Ihnen mehr zusagt. Bitte wählen Sie immer einen Kurs.

L4. Auswahlmöglichkeit	Kurs A	Kurs B
**Kurszeiten**	Morgens (8-10 Uhr)	Vormittags (10-12 Uhr)
**Dauer Geh-/Fahrweg bis zum BIG-Kurs**	Maximal 30 Minuten	Maximal 10 Minuten
**Zusätzliche soziale Aktivitäten im Rahmen von BIG organisiert**	Keine zusätzliche Aktivität	Keine zusätzliche Aktivität
**Berücksichtigung von Interessen und Wünsche bei der weiteren Kursplanung**	Interessen/Wünsche werden berücksichtigt	Interessen/Wünsche werden nicht berücksichtigt
**Kurskosten (pro Termin)**	6,50 €	5,00 €
**Welchen Kurs bevorzugen Sie? (Kreuzen Sie bitte das entsprechende Kästchen an)**	☐	☐

Bitte wählen Sie den Kurs aus, der Ihnen mehr zusagt. Bitte wählen Sie immer einen Kurs.

L5. Auswahlmöglichkeit	Kurs A	Kurs B
**Kurszeiten**	Nachmittags (16-18 Uhr)	Abends (18-20 Uhr)
**Dauer Geh-/Fahrweg bis zum BIG-Kurs**	Maximal 10 Minuten	Maximal 30 Minuten
**Zusätzliche soziale Aktivitäten im Rahmen von BIG organisiert**	Keine zusätzliche Aktivität	Zusätzliche soziale Aktivitäten
**Berücksichtigung von Interessen und Wünsche bei der weiteren Kursplanung**	Interessen/Wünsche werden berücksichtigt	Interessen/Wünsche werden nicht berücksichtigt
**Kurskosten (pro Termin)**	3,50 €	8,00 €
**Welchen Kurs bevorzugen Sie? (Kreuzen Sie bitte das entsprechende Kästchen an)**	☐	☐

Bitte wählen Sie den Kurs aus, der Ihnen mehr zusagt. Bitte wählen Sie immer einen Kurs.

L6. Auswahlmöglichkeit	Kurs A	Kurs B
**Kurszeiten**	Nachmittags (16-18 Uhr)	Morgens (8-10 Uhr)
**Dauer Geh-/Fahrweg bis zum BIG-Kurs**	Maximal 30 Minuten	Maximal 10 Minuten
**Zusätzliche soziale Aktivitäten im Rahmen von BIG organisiert**	Zusätzliche soziale Aktivitäten	Keine zusätzliche Aktivität
**Berücksichtigung von Interessen und Wünsche bei der weiteren Kursplanung**	Interessen/Wünsche werden berücksichtigt	Interessen/Wünsche werden nicht berücksichtigt
**Kurskosten (pro Termin)**	2,00 €	9,50 €
**Welchen Kurs bevorzugen Sie? (Kreuzen Sie bitte das entsprechende Kästchen an)**	☐	☐

Bitte wählen Sie den Kurs aus, der Ihnen mehr zusagt. Bitte wählen Sie immer einen Kurs.

For peer review only - http://bmjopen.bmj.com/site/about/guidelines.xhtml

L7. Auswahlmöglichkeit	Kurs A	Kurs B
**Kurszeiten**	Morgens (8-10 Uhr)	Nachmittags (16-18 Uhr)
**Dauer Geh-/Fahrweg bis zum BIG-Kurs**	Maximal 20 Minuten	Maximal 30 Minuten
**Zusätzliche soziale Aktivitäten im Rahmen von BIG organisiert**	Zusätzliche soziale Aktivitäten	Keine zusätzliche Aktivität
**Berücksichtigung von Interessen und Wünsche bei der weiteren Kursplanung**	Interessen/Wünsche werden berücksichtigt	Interessen/Wünsche werden nicht berücksichtigt
**Kurskosten (pro Termin)**	2,00 €	6,50 €
**Welchen Kurs bevorzugen Sie? (Kreuzen Sie bitte das entsprechende Kästchen an)**	☐	☐

Bitte wählen Sie den Kurs aus, der Ihnen mehr zusagt. Bitte wählen Sie immer einen Kurs.

L8. Auswahlmöglichkeit	Kurs A	Kurs B
**Kurszeiten**	Vormittags (10-12 Uhr)	Morgens (8-10 Uhr)
**Dauer Geh-/Fahrweg bis zum BIG-Kurs**	Maximal 10 Minuten	Maximal 30 Minuten
**Zusätzliche soziale Aktivitäten im Rahmen von BIG organisiert**	Keine zusätzliche Aktivität	Keine zusätzliche Aktivität
**Berücksichtigung von Interessen und Wünsche bei der weiteren Kursplanung**	Interessen/Wünsche werden nicht berücksichtigt	Interesse/Wünsche werden berücksichtigt
**Kurskosten (pro Termin)**	5,00 €	6,50 €
**Welchen Kurs bevorzugen Sie? (Kreuzen Sie bitte das entsprechende Kästchen an)**	☐	☐

Bitte wählen Sie den Kurs aus, der Ihnen mehr zusagt. Bitte wählen Sie immer einen Kurs.

L9. Auswahlmöglichkeit	Kurs A	Kurs B
**Kurszeiten**	Vormittags (10-12 Uhr)	Abends (18-20 Uhr)
**Dauer Geh-/Fahrweg bis zum BIG-Kurs**	Maximal 30 Minuten	Maximal 10 Minuten
**Zusätzliche soziale Aktivitäten im Rahmen von BIG organisiert**	Zusätzliche soziale Aktivitäten	Zusätzliche soziale Aktivitäten
**Berücksichtigung von Interessen und Wünsche bei der weiteren Kursplanung**	Interessen/Wünsche werden nicht berücksichtigt	Interessen/Wünsche werden berücksichtigt
**Kurskosten (pro Termin)**	2,00 €	8,00 €
**Welchen Kurs bevorzugen Sie? (Kreuzen Sie bitte das entsprechende Kästchen an)**	☐	☐

For peer review only - http://bmjopen.bmj.com/site/about/guidelines.xhtml

References

Aboagye, E. (2017). Valuing Individuals' Preferences and Health Choices of Physical Exercise. *Pain Ther*, 6(1), 85-91. https://doi.org/10.1007/s40122-017-0067-4

Aboagye, E., Hagberg, J., Axén, I., Kwak, L., Lohela-Karlsson, M., Skillgate, E., Dahlgren, G., & Jensen, I. (2017). Individual preferences for physical exercise as secondary prevention for non-specific low back pain: A discrete choice experiment. *PLoS One*, 12(12), e0187709. https://doi.org/10.1371/journal.pone.0187709

Alayli-Goebbels, A. F., Dellaert, B. G., Knox, S. A., Ament, A. J., Lakerveld, J., Bot, S. D., Nijpels, G., & Severens, J. L. (2013). Consumer preferences for health and nonhealth outcomes of health promotion: results from a discrete choice experiment. *Value Health*, 16(1), 114-123. https://doi.org/10.1016/j.jval.2012.08.2211

Benning, T. M., Dellaert, B. G. C., & Arentze, T. A. (2020). The impact of health vs. non-health goals on individuals' lifestyle program choices: a discrete choice experiment approach. *BMC Public Health*, 20(1), 411. https://doi.org/10.1186/s12889-020-8416-3

Brown, D. S., Finkelstein, E. A., Brown, D. R., Buchner, D. M., & Johnson, F. R. (2009). Estimating older adults' preferences for walking programs via conjoint analysis. *Am J Prev Med*, 36(3), 201-207.e204. https://doi.org/10.1016/j.amepre.2008.10.014

Dintsios, C. M., Chernyak, N., Grehl, B., & Icks, A. (2018). Quantified patient preferences for lifestyle intervention programs for diabetes prevention-a protocol for a systematic review. *Syst Rev*, 7(1), 214. https://doi.org/10.1186/s13643-018-0884-5

Farooqui, M. A., Tan, Y. T., Bilger, M., & Finkelstein, E. A. (2014). Effects of financial incentives on motivating physical activity among older adults: results from a discrete choice experiment. *BMC Public Health*, 14, 141. https://doi.org/10.1186/1471-2458-14-141

Ferreira, G. E., Howard, K., Zadro, J. R., O'Keeffe, M., Lin, C. C., & Maher, C. G. (2020). People considering exercise to prevent low back pain recurrence prefer exercise programs that differ from programs known to be effective: a discrete choice experiment. *J Physiother*, 66(4), 249-255. https://doi.org/10.1016/j.jphys.2020.09.011

Geidl, W., Knocke, K., Schupp, W., & Pfeifer, K. (2018). Measuring stroke patients' exercise preferences using a discrete choice experiment. *Neurol Int*, 10(1), 6993. https://doi.org/10.4081/ni.2018.6993

Giles, E. L., Becker, F., Ternent, L., Sniehotta, F. F., McColl, E., & Adams, J. (2016). Acceptability of Financial Incentives for Health Behaviours: A Discrete Choice Experiment. *PLoS One*, 11(6), e0157403. https://doi.org/10.1371/journal.pone.0157403

Kjaer, T., Gyrd-Hansen, D., & Willaing, I. (2006). Investigating patients' preferences for cardiac rehabilitation in Denmark. *Int J Technol Assess Health Care*, 22(2), 211-218. https://doi.org/10.1017/s0266462306051038

Matsushita, M., Harada, K., & Arao, T. (2017). [Incentive program to strengthen motivation for increasing physical activity via conjoint analysis]. *Nihon Koshu Eisei Zasshi*, 64(4), 197-206. https://doi.org/10.11236/jph.64.4_197

Molema, C., Veldwijk, J., Wendel-Vos, W., de Wit, A., van de Goor, I., & Schuit, J. (2019). Chronically ill patients' preferences for a financial incentive in a lifestyle intervention. Results of a discrete choice experiment. *PLoS One*, 14(7), e0219112. https://doi.org/10.1371/journal.pone.0219112

For peer review only - http://bmjopen.bmj.com/site/about/guidelines.xhtml

Owen, K., Pettman, T., Haas, M., Viney, R., & Misan, G. (2010). Individual preferences for diet and exercise programmes: changes over a lifestyle intervention and their link with outcomes. *Public Health Nutr*, 13(2), 245-252. https://doi.org/10.1017/s1368980009990784

Paul, S. S., Canning, C. G., Löfgren, N., Sherrington, C., Lee, D. C., Bampton, J., & Howard, K. (2021). People with Parkinson's disease are more willing to do additional exercise if the exercise program has specific attributes: a discrete choice experiment. *J Physiother*, 67(1), 49-55. https://doi.org/10.1016/j.jphys.2020.12.007

Pinto, D., Bockenholt, U., Lee, J., Chang, R. W., Sharma, L., Finn, D. J., Heinemann, A. W., Holl, J. L., & Hansen, P. (2019). Preferences for physical activity: a conjoint analysis involving people with chronic knee pain. *Osteoarthritis Cartilage*, 27(2), 240-247. https://doi.org/10.1016/j.joca.2018.10.002

Pinto, D., Danilovich, M. K., Hansen, P., Finn, D. J., Chang, R. W., Holl, J. L., Heinemann, A. W., & Bockenholt, U. (2017). Qualitative Development of a Discrete Choice Experiment for Physical Activity Interventions to Improve Knee Osteoarthritis. *Arch Phys Med Rehabil*, 98(6), 1210-1216.e1211. https://doi.org/10.1016/j.apmr.2016.11.024

Ramirez, M., Wu, S., & Beale, E. (2016). Designing a Text Messaging Intervention to Improve Physical Activity Behavior Among Low-Income Latino Patients With Diabetes: A Discrete-Choice Experiment, Los Angeles, 2014-2015. *Prev Chronic Dis*, 13, E171. https://doi.org/10.5888/pcd13.160035

Robles, L. A., Wright, S. J., Hackshaw-McGeagh, L., Shingler, E., Shiridzinomwa, C., Lane, J. A., Martin, R. M., & Burden, S. (2020). Prostate cancer survivors' preferences on the delivery of diet and lifestyle advice: a pilot best-worst discrete choice experiment. *Pilot Feasibility Stud*, 6, 2. https://doi.org/10.1186/s40814-019-0549-8

Ryan, M., Yi, D., Avenell, A., Douglas, F., Aucott, L., van Teijlingen, E., & Vale, L. (2015). Gaining pounds by losing pounds: preferences for lifestyle interventions to reduce obesity. *Health Econ Policy Law*, 10(2), 161-182. https://doi.org/10.1017/s1744133114000413

Salampessy, B. H., Veldwijk, J., Jantine Schuit, A., van den Brekel-Dijkstra, K., Neslo, R. E., Ardine de Wit, G., & Lambooij, M. S. (2015). The Predictive Value of Discrete Choice Experiments in Public Health: An Exploratory Application. *Patient*, 8(6), 521-529. https://doi.org/10.1007/s40271-015-0115-2

Sommer, J., Dyczmons, J., Grobosch, S., Gontscharuk, V., Vomhof, M., Roden, M., & Icks, A. (2020). Preferences of people with type 2 diabetes for telemedical lifestyle programmes in Germany: protocol of a discrete choice experiment. *BMJ Open*, 10(9), e036995. https://doi.org/10.1136/bmjopen-2020-036995

van Gils, P. F., Lambooij, M. S., Flanderijn, M. H., van den Berg, M., de Wit, G. A., Schuit, A. J., Struijs, J. N., & van den Berg, B. (2011). Willingness to participate in a lifestyle intervention program of patients with type 2 diabetes mellitus: a conjoint analysis. *Patient Prefer Adherence*, 5, 537-546. https://doi.org/10.2147/ppa.S16854

Veldwijk, J., Lambooij, M. S., de Bekker-Grob, E. W., Smit, H. A., & de Wit, G. A. (2014). The effect of including an opt-out option in discrete choice experiments. *PLoS One*, 9(11), e111805. https://doi.org/10.1371/journal.pone.0111805

Veldwijk, J., Lambooij, M. S., van Gils, P. F., Struijs, J. N., Smit, H. A., & de Wit, G. A. (2013). Type 2 diabetes patients' preferences and willingness to pay for lifestyle programs: a discrete choice experiment. *BMC Public Health*, 13, 1099. https://doi.org/10.1186/1471-2458-13-1099

For peer review only - http://bmjopen.bmj.com/site/about/guidelines.xhtml

Wanders, J. O., Veldwijk, J., de Wit, G. A., Hart, H. E., van Gils, P. F., & Lambooij, M. S. (2014). The effect of out-of-pocket costs and financial rewards in a discrete choice experiment: an application to lifestyle programs. *BMC Public Health*, 14, 870. https://doi.org/10.1186/1471-2458-14-870

For peer review only

For peer review only - http://bmjopen.bmj.com/site/about/guidelines.xhtml

BMJ Open

Preferences of women in difficult life situations for a physical activity programme: protocol of a discrete choice experiment in the German NU-BIG project

Journal:	*BMJ Open*
Manuscript ID	bmjopen-2022-067235.R2
Article Type:	Protocol
Date Submitted by the Author:	26-Jun-2023
Complete List of Authors:	Pedron, Sara; Technical University of Munich, Department of Sport and Health Sciences; Helmholtz Center Munich German Research Center for Environmental Health, Institute of Health Economics and Healthcare Management<br>Herbert-Maul, Annika; Friedrich-Alexander-Universitat Erlangen-Nurnberg, Department of Sport Science and Sport<br>Sauter, Alexandra; University of Regensburg, Department for Epidemiology and Preventive Medicine, Medical Sociology<br>Linder, Stephanie; Friedrich-Alexander-Universitat Erlangen-Nurnberg, Department of Sport Science and Sport<br>Sommer, Raluca; Friedrich-Alexander-Universitat Erlangen-Nurnberg, Department of Sport Science and Sport<br>Vomhof, Markus; Heinrich Heine University Düsseldorf; German Center for Diabetes Research<br>Gontscharuk, Veronika; Heinrich Heine University Düsseldorf; German Center for Diabetes Research<br>Abu-Omar, Karim ; Friedrich-Alexander-Universitat Erlangen-Nurnberg, Department of Sport Science and Sport<br>Thiel, Ansgar; Eberhard Karls Universitat Tubingen, Institute of Sports Science, Social and Public Health Sciences and Interfaculty Research Institute for Sport and Physical Activity<br>Ziemainz, Heiko; Friedrich-Alexander-Universitat Erlangen-Nurnberg, Department of Sport Science and Sport<br>Holle, Rolf; Ludwig-Maximilians-Universitat Munchen, Institute for Medical Informatics, Biometry and Epidemiology<br>Laxy, Michael; Technical University of Munich, Department of Sport and Health Sciences; Helmholtz Center Munich German Research Center for Environmental Health, Institute of Health Economics and Health Care Management
**Primary Subject Heading**:	Public health
Secondary Subject Heading:	Health economics, Sports and exercise medicine
Keywords:	HEALTH ECONOMICS, SPORTS MEDICINE, SOCIAL MEDICINE

SCHOLARONE™
Manuscripts

Preferences of women in difficult life situations for a physical activity programme: protocol of a discrete choice experiment in the German NU-BIG project

Sara Pedron [a,b,c], Annika Herbert-Maul [d], Alexandra Sauter [e], Stephanie Linder [d], Raluca Sommer [d], Markus Vomhof [f,c,g], Veronika Gontscharuk [f,c,g], Karim Abu-Omar [d], Ansgar Thiel [h], Heiko Ziemainz [d], Rolf Holle [h] & Michael Laxy [a,b,c]*

[a] Professorship of Public Health and Prevention, Department of Sport and Health Sciences, Technical University of Munich, Munich, Germany
[b] Institute of Health Economics and Health Care Management, Helmholtz Center Munich, German Research Center for Environmental Health (GmbH), Munich, Germany
[c] German Center for Diabetes Research, Neuherberg, Germany
[d] Department of Sport Science and Sport, Friedrich-Alexander-Universität Erlangen-Nuremberg, Erlangen, Germany
[e] Department for Epidemiology and Preventive Medicine, Medical Sociology, University of Regensburg, Regensburg, Germany
[f] Institute for Health Services Research and Health Economics, Centre for Health and Society, Medical Faculty and University Hospital Düsseldorf, Heinrich-Heine-University Düsseldorf, Düsseldorf, Germany.
[g] Institute for Health Services Research and Health Economics, German Diabetes Center (DDZ), Leibniz Center for Diabetes Research at the Heinrich-Heine-University Düsseldorf, Düsseldorf, Germany
[h] Institute of Sports Science, Social and Public Health Sciences and Interfaculty Research Institute for Sport and Physical Activity, Eberhard Karls University Tübingen, Tübingen, Germany
[h] Institute for Medical Informatics, Biometry and Epidemiology, University Hospital, Ludwig-Maximilian-University Munich, Munich, Germany

Sara Pedron (sara.pedron@tum.de)
Annika Herbert-Maul (annika.herbert-maul@fau.de)
Alexandra Sauter (alexandra.sauter@klinik.uni-regensburg.de)
Stephanie Linder (stephanie.linder@fau.de)
Raluca Sommer (raluca.sommer@fau.de)
Markus Vomhof (Markus.Vomhof@uni-duesseldorf.de)
Veronika Gontscharuk (Veronika.Gontscharuk@ddz.de)
Karim Abu-Omar (karim.abu-omar@fau.de)
Ansgar Thiel (ansgar.thiel@uni-tuebingen.de)
Heiko Ziemainz (heiko.ziemainz@fau.de)
Rolf Holle (rholle@ibe.med.uni-muenchen.de)
Michael Laxy (Michael.laxy@tum.de)

*Corresponding author:
Sara Pedron, PhD
Technical University of Munich, Professorship of Public Health and Prevention, Georg-Brauchle-Ring 60, 80992 München, Germany
sara.pedron@tum.de
+49 (0) 89/289 24979

Word count: 4667

For peer review only

Abstract

Introduction: The BIG project ('Bewegung als Investition in die Gesundheit', i.e. 'Movement as Investment in Health') was developed in 2005 as a community-based participatory research programme to offer accessible opportunities for physical activity to women in difficult life situations. Since then, the programme has been expanded to eight sites in Germany. A systematic evaluation of BIG is currently being conducted. As part of this effort, we strive to understand the preferences of participating women for different aspects of the programme, and to analyse their willingness to pay.

Methods and analysis: In this protocol, we describe the development and analysis plan of a discrete choice experiment (DCE) to investigate participants' preferences for a physical activity programme for women in difficult life situations. The experiment will be embedded in a questionnaire covering several aspects of participation in the programme (e.g. reach, efficacy, further effects) and the socioeconomic characteristics of all active participants. After a thorough search of the literature, BIG documents review and expert interviews, we identified five important attributes of the programme: course times, travel time to the course venue, additional social activities organised by BIG, consideration of wishes and interests for the further planning of courses, costs per course unit. Thereafter, we piloted the experiment with a sample of participants from the target group. After data collection, the experiment will be analysed using a conditional logit model and a latent class analysis to assess eventual heterogeneity in preferences.

Ethics and dissemination: Understanding women's preferences will provide useful insights for the further development of the programme and ultimately increase participation and retention. The questionnaire, the included DCE and the pre-test on participants received ethical approval (Application no. 20-247_1-B). We plan to disseminate the results of the DCE in peer-reviewed journals, national conferences and among participants and programme coordinators and organisers.

Keywords: discrete choice experiment, disadvantaged groups, physical activity, preferences, willingness to pay

For peer review only

Strengths and limitations

- This DCE is the first to evaluate participants' preferences for cooperative planning and social inclusion aspects of a community-based participatory research programme in the physical activity promotion field.
- This DCE is the first to target preferences for physical activity opportunities in a vulnerable group, namely women in difficult life situations.
- This DCE necessarily represents a simplification of the decision-making process of women participating in the programme, where several other aspects might play a role in driving women's decisions to keep participating.
- As the target group consists of women who already participate in the programme, we will analyse preferences for sustained participation, but will not be able to evaluate preferences for starting anew in the offered physical activity courses.

For peer review only

For peer review only - http://bmjopen.bmj.com/site/about/guidelines.xhtml

1. Introduction

Several studies have demonstrated that leisure time physical activity follows a socioeconomic gradient: individuals with a lower education, lower income and lower occupational position were consistently shown to engage less frequently in physical activity than their higher socioeconomic status (SES) counterparts [1–3]. In fact, socioeconomic factors are one of the major determinants of cardiovascular and metabolic conditions, which are among the most important drivers of morbidity and mortality worldwide [4]. The effect of socioeconomic factors on cardio-metabolic health is multifaceted, but runs mostly via physiological, psychosocial and behavioural factors, with varying degrees of influence in different contexts and life stages [5]. Furthermore, several reviews from different contexts have shown a gender gap in physical activity, demonstrating that women are more at risk of sedentary behaviour than men because of several barriers, such as family responsibilities, safety concerns or lack of culturally appropriate sport opportunities [6–10]. Therefore, the intersection of socioeconomic factors and sex-specific factors makes women from disadvantaged socioeconomic backgrounds a vulnerable group at serious risk of sedentary behaviour.

The fact that women from disadvantaged socioeconomic backgrounds are especially at risk for physical inactivity has also been demonstrated in the German context [11–14]. Therefore, policies and programmes aiming to reduce barriers to participation (economic, time-related, cultural and social) and increase the amount of leisure time physical activity, especially in socially disadvantaged populations, are needed. Although the majority of programmes involve individual cognitive-behavioural approaches to enhance physical activity [15], it has been argued that a broader approach also targeting structural aspects might be necessary to improve lifestyles effectively [16]. This is especially the case if such programmes manage to activate already existing assets in the communities and increase the offer of targeted possibilities, for example by directly considering and overcoming actual and perceived barriers in the targeted groups [17].

One example of such programmes from the German context is the 'BIG' project ('Bewegung als Investition in Gesundheit' – 'Movement as Investment in Health'). The programme is based on a community-based participatory research approach, i.e. a research paradigm that aims primarily at closer communication with and involvement of the communities that are the object of the investigation to shape, manage and evaluate the intervention or the context studied [18]. In this case, the programme specifically targets physical activity courses for women in difficult life situations, e.g. having a low household income, a migration background, being unemployed, reliant on welfare payments or single mothers [19, 20]. The programme has since expanded to

For peer review only - http://bmjopen.bmj.com/site/about/guidelines.xhtml

a further 20 communities (eight of them are still active). In each of these, a 'cooperative planning approach' was adopted, where women from the target group were explicitly and regularly included in the planning and organisation of the courses, with the aim of overcoming barriers and increasing participation and retention [21–26].

Even though the project strives to include women from the target group in the planning and organisational phase, little is known about their preferences. Understanding and evaluating the preferences of participating women is important in order to develop the programme further, make it more diversified to reach specific groups of the population and encourage participation over a longer period of time. In order to elicit patient or participant preferences, economic stated-preferences methods such as discrete choice experiments (DCEs) have been used increasingly in healthcare contexts [27]. This preference elicitation method relies on the economic theory of demand [28] and on random utility theory [29, 30]. According to these theories, when confronted with a choice, individuals will choose the option with the highest level of utility, i.e. the option whose characteristics combined generate the highest utility value for the individual. DCE is a choice-based method where individuals are confronted with a series of hypothetical choice scenarios to simulate realistic choices. Respondents have to trade-off one option with all its characteristics against a second option, finally indicating which one of the two they prefer. By evaluating the final choice pattern, the method allows the computation of the relative importance of attributes. If an attribute representing costs is also assessed, then the willingness to pay (WTP) for levels can be elicited, i.e. how much on average a participant is willing to pay for an improvement in one attribute [31].

Aims of this protocol and of the DCE

Using a DCE, we aim to understand and quantify the preferences of participating women for specific structural characteristics of the BIG programme and to investigate how they differ with respect to socio-demographic and cultural backgrounds. The aim of the present protocol is to outline the preparatory steps for the DCE and to present the final version of the experiment used in the questionnaire. This DCE is the first to evaluate the cooperative planning aspect and social activities component as structural characteristics of a community-based participatory research programme in the physical activity promotion field. Investigating preferences for the BIG physical activity courses will help to deduce possible reasons why women from disadvantaged social backgrounds participate, using a tool that mimics real life choices including a limited offer of alternatives and potential trade-offs. Furthermore, the findings will also help to develop the programme further at the active BIG sites and help to achieve a greater involvement of

For peer review only - http://bmjopen.bmj.com/site/about/guidelines.xhtml

participating women by aligning the programmes to preferences and thus fostering their integration and the benefits to women from participation. Finally, identifying components that are essential or highly valued by the target group can be useful in informing policymakers to design better strategies for physical activity promotion for women in difficult life situations.

2. Methods and analysis

In this section, we explain the background and context in which the DCE is embedded (2.1 and 2.2), the preparatory steps that were carried out to set up the DCE (2.3), the planned analyses (2.4) and patient involvement activities (2.5).

2.1 The BIG project

The BIG project ('Bewegung als Investition in Gesundheit', i.e. 'Movement as Investment in Health') was started in 2005 by the Friedrich-Alexander-Universität Erlangen-Nürnberg (FAU), Germany. The project specifically strives to increase physical activity among women in difficult life situations [19]. Since its start, the programme has been transferred to another 20 communities/municipalities, mostly but not only in Bavaria (Germany) [21]. Currently, the programme is being actively implemented at eight sites, with three sites in the starting phase. In the remaining nine sites, the programme was stopped because of different implementation and sustainability issues in the local communities.

The target groups of the BIG programmes are women in difficult life situations, which include women with a low income, a migration background, unemployed women, women relying on welfare payments or single mothers. However, BIG courses are open to all women who decide to participate.

Using a 'cooperative planning approach' [22–25], the programme also aims to involve women from the target group in the planning, organisation and evaluation stages of the project. In this way, highly tailored exercise programmes could be constructed by eliminating barriers (e.g. by offering childcare possibilities during the course, by offering courses that respect religious norms, etc.). Ultimately, the programme aims to maximise the participation and inclusion of women, thereby achieving broader empowerment, with potential effects on self-efficacy and other health behaviours (e.g. healthy diet and smoking). Furthermore, this approach facilitates the inclusion of other women from their social network with a similar socioeconomic background, fostering integration and social exchange within the programme itself.

At each site, the programme offers several courses, in both gyms/halls (fitness courses) and swimming pools (water courses). The fitness courses include, for example, Pilates, yoga and

For peer review only - http://bmjopen.bmj.com/site/about/guidelines.xhtml

aerobics. The water courses include water fitness and swimming courses. Furthermore, at some sites, additional social and cultural activities are organised, such as a 'breakfast for women' ('Frauenfrühstück') several times per year, cook-in evenings with healthy dishes from different cultures and further occasions for mutual exchange. Given the participatory nature of the programme and the active role that women have in shaping its content and forms, the courses offered and additional activities differ from site to site (e.g. workshops for conflict management, stress reduction or self-defence) [21]. A detailed overview of the types of courses offered at each active site in the last year before the pandemic (2019), the number of participants in each course and the additional activities can be found in Appendix 1.

For the success of the programme, women from the target group should be reached by these offers and should agree to participate. Furthermore, another very important aspect is their continued participation, as only sustained participation leads to health improvements. Therefore, understanding the factors that drive their sustained participation (e.g. via a DCE) is of paramount importance.

2.2 The NU-BIG evaluation and the target group

The present DCE is part of a general effort to evaluate the impact of the BIG programme, called the NU-BIG study ('Nachuntersuchung des BIG-Projekts'), funded by the German Federal Ministry of Education and Research. The evaluation will follow the RE-AIM framework and was described in detail in the study protocol for the NU-BIG project [32]. The evaluation focuses on investigating the long-term effects of BIG on health behaviour, inclusion and social participation, as well as factors facilitating long-term implementation and transfer. The study consists of a cross-sectional evaluation using a mixed-methods approach, including questionnaires for participating women, qualitative interviews for local organisers and focus groups with participating women.

The present DCE will be part of the questionnaire, which will be distributed to all women who participate in BIG courses between May 2022 and winter 2022/2023 at active sites (excluding sites in the starting phase). Based on pre-pandemic participation rates, it was estimated that the study population will amount to around 800 participating women at eight sites. The questionnaire will be administered as a 'paper and pen' survey, by handing the paper questionnaire to the target group in person during the BIG courses. Owing to the diversity of BIG activities at each site, an adjustment of the mode of administration will be required for several courses. In consultation with the trainers for each course, we will adapt the way we distribute the questionnaire to the specific requirements of each course (e.g. outdoor courses,

For peer review only - http://bmjopen.bmj.com/site/about/guidelines.xhtml

swimming classes) and to the specific linguistic background of participating women by making the questionnaire available in German, Arabic, English, Russian and Turkish.

2.3 Development of the DCE to assess participant preferences

To derive preferences, it is important to carefully develop and then transfer the DCE into an experimental design following methodological standards [29, 33]. The DCE is a type of conjoint analysis, i.e. a stated-preference method that involves an indirect comparison of choices, for example via ranking, rating or choice designs, to evaluate and quantify preferences for several attributes of an intervention [33]. Therefore, designing a DCE involves a thorough identification of attributes and levels that define the intervention, and the construction of choice sets within the experimental design. In the next paragraphs, we describe this process in detail in the context of the NU-BIG study.

2.3.1 Problem and target group definition

The main aim of the DCE is understanding which aspects of the programme favour sustained participation in the programme and help to keep participating women interested in new courses. The target group consists of all women who are actively participating in BIG courses, as the questionnaire will be handed in during classes starting from May 2022 until winter 2022/2023.

2.3.2 Identification of attributes and levels and transfer to the experimental design

The DCE will be embedded in the questionnaire and will present women with a series of hypothetical comparisons between two physical activity courses with predefined characteristics, asking them to choose which option they like the most.

In a first step, all programme characteristics (attributes) that might influence individual preferences for participation in BIG courses should be identified. At the same time, appropriate levels of these attributes should be determined. All included attributes should be relevant for the choice of course, should be easily influenced and close to reality, should have a substitutive relation and should not be dominant [34]. To identify attributes and their relative levels, we followed a systematic approach. We describe all steps below.

Compilation of evidence (literature search)

The literature was searched for physical activity programmes/interventions that were evaluated using a DCE. The full search strategy, flow chart and results can be browsed in Appendix 2. We identified 19 studies, for which we systematically retrieved attributes and corresponding

For peer review only - http://bmjopen.bmj.com/site/about/guidelines.xhtml

levels. We divided these attributes into meaningful groups: exercise frequency and duration, context, type of exercise, outcomes and goals, schedule, costs, travel time and location, support and other. It is important to note that no study specifically included women exclusively as a target group.

Compilation of evidence (programme specific)

We reviewed previous documents from the BIG programme (qualitative and quantitative data, protocol of the kick-off meeting) to identify potential attributes and levels in addition to those identified by the literature search. Each document was searched independently by two researchers from the group (AS, SL, AHM, SP). Resulting attributes and levels were then pooled, discussed by the group (SP, AHM, SL, AS) and compared with the results from the systematic literature search (full list available in Appendix 2).

In a further step, we selected only attributes that are applicable in the context of the BIG courses and that are already implemented or potentially implementable in the programme. For example, attributes and levels that included mixed classes with men or individual courses were excluded as BIG only offers group courses for women. Furthermore, attributes involving the presence of a personal coach were also excluded as this is not intended or feasible in BIG. Additionally, attributes referring to programmes targeting specific illnesses were also excluded.

This selection led to a list of 10 potentially important attributes (Appendix 3), which served as the basis for discussion with the experts in the following step.

Consultation with experts

This step involved an online discussion of the previously identified list of 10 attributes and their respective levels with five 'BIG experts'. They are BIG course instructors, organisers or long-term participants, who actively took part in shaping BIG courses at their site and who are routinely interviewed and involved in the planning phases of the NU-BIG evaluation. The expert group of women was created at the beginning of the NU-BIG evaluation to implement the grounding principles of community-based participatory research and includes highly motivated women who are active at their sites in different roles. They receive a small compensation payment for their participation and are involved in multiple projects within the NU-BIG evaluation.

In the meeting, which was headed by SP, AHM and AS, we explained to the experts the goals of the DCE, and we presented them with a first example of trade-off decisions within a fictitious choice set. After this, each attribute and the respective level was thoroughly discussed in the

For peer review only - http://bmjopen.bmj.com/site/about/guidelines.xhtml

group. Thereby, we specifically asked whether the 10 reported characteristics are relevant ones and whether they represent important drivers in the decision to participate. Furthermore, we discussed their clarity and understandability, and we explicitly asked whether some important aspects might be missing. At the end of the meeting, we also asked the five experts to rank the attributes based on the perceived relevance for participating women with an interactive tool to spark further discussion.

The discussion led to interesting insights into the reasons for participating from the point of view of the course instructors and organisers. Following this discussion, one additional attribute was identified as an important one ('flexible intensity of the course') and two others were re-framed in a different way. We report the results of this discussion, including the new list of attributes and levels, in Appendix 3.

Based on these insights, the list of 11 attributes was analysed further by researchers (SP, AHM, MV). In our screening of the attributes, we ensured that all attributes were relevant for the choice of course and were easily influenced within the BIG programme. Furthermore, we also ensured that they were in a substitutive relation (i.e. the worsening of one attribute can be compensated by an improvement in another attribute) and were not dominant (i.e. no attribute should be a necessary criterion for or against participation) [34]. The detailed decision steps and the resulting list of attributes and levels are reported in Appendix 3. The resulting list includes five attributes, i.e. (1) course time, (2) travel time to the BIG course, (3) additional activities organised by BIG, (4) consideration of wishes or interests and (5) costs per course unit. Based on discussion with the experts, we decided to keep some attributes fixed, i.e. to introduce an initial statement clarifying that all courses would have specific characteristics. These include the presence of childcare, the presence of trial lessons, course duration and the framework (e.g. the hall is not visible from the outside). These fixed attributes are in fact usual components of all BIG courses that are already implemented to reduce barriers to participation.

Pre-test with participants

Based on the selected attributes and levels, a preliminary experimental design was conducted to prepare a full DCE for a pre-test. We tested the full DCE questionnaire with a set of voluntary participants from the target group. To this end, we recruited participating women and performed semi-structured qualitative interviews using the think aloud technique based on a topic guide until a saturation of new content was achieved [35, 36]. This led us to include six women from two BIG sites (Erlangen and Nürnberg). The interviews were carried out by telephone and were recorded after asking for the consent of the participants. The interviewee was asked to read a

For peer review only - http://bmjopen.bmj.com/site/about/guidelines.xhtml

paper version of the questionnaire that was previously sent by mail, and loudly report her thoughts and choices. The notes and recordings of the phone call were then revised by the interviewer (SP) and by a second researcher (MV), in order to identify the main problems and any improvements necessary.

We are aware that both samples selected for the expert interviews and the pre-test might not be representative of the targeted population of participating women. However, it was impossible for the study team to directly access participants from the study population (the study was planned before late spring 2022, when only selected courses were taking place because of Covid-19 restrictions). The potential bias induced by this issue will be considered in the final evaluation.

In the pre-test with participants, we paid particular attention to the following criteria:

(1) Are all the most important attributes included in the DCE?
(2) Is one or more of the included attributes dominant?
(3) Are all levels appropriate?
(4) Are the task and the questionnaire understandable and feasible?

The interviews followed a pre-specified outline and were recorded for quality assurance and analysed using content analysis methods by two researchers (SP and MV) [37]. This step led to a general confirmation of the understandability of the experiment among women with different backgrounds, linguistic skills and from different cities. The task was understood properly, and all women engaged in the trade-offs proposed in the DCE with ease. Drawing from the feedback of participating women, we made some adjustments in missing context information (e.g. the courses take place only during the week, the number of appointments was specified). Furthermore, we modified the range of costs per training unit proposed in the DCE. Initially, we considered a price range between 0 € and 5 € per training unit, with 1 € intervals (the levels were: 0, 1, 2, 3, 4, 5 €). However, the price was perceived as an irrelevant attribute by the participants, as even the highest level was considered very affordable and comparable with the levels below. Based on specific indications of participating women on the price of courses that they currently participate in (up to 8 € per course unit), we decided to increase the price range to 9.5 €. Furthermore, we received mixed feedback regarding including the level 0 €: although some women perceived this as very advantageous, others interpreted this as a sign of poor quality. As we assume linearity in the cost attribute, we decided to eliminate this level. The final cost levels thus range between 2 € and 9.5 €, with 1.5 € intervals.

For peer review only - http://bmjopen.bmj.com/site/about/guidelines.xhtml

Table 1: Attributes and levels included

Attributes	Levels
**Course time**	Early morning (8–10 am)
	Morning (10 am–12 pm)
	Afternoon (4–6 pm)
	Evening (6–8 pm)
**Travel time to the BIG course**	Maximum 10 minutes
	Maximum 20 minutes
	Maximum 30 minutes
**Additional activities by BIG**	No additional social activity is organised
	Additional social activities are organised
**Consideration of wishes and interests for the further planning of courses**	Interests/wishes are not asked/considered
	Interests/wishes are asked/considered
**Costs per course unit**	2.00 €
	3.50 €
	5.00 €
	6.50 €
	8.00 €
	9.50 €

Notes: the original version in the German language is available in Appendix 4.

Final attributes and levels included

The final list of attributes and levels included in the DCE is reported in Table 1 (original language version in Appendix 4). We included five attributes, with a varying number of levels. The first attribute is the course time, i.e. when the course takes place during the day. For this attribute, we considered four levels: early morning, morning, afternoon and evening. The second attribute represents the travel time needed to reach the course location. This encompasses three levels: maximum 10, 20 or 30 minutes. The third attribute represents the possibility of organised social activities within the BIG course, such as joint breakfasts (called 'women's breakfasts') or dinners where all participating women are invited to socialise outside of the courses. This attribute has two levels: activities are organised or not. The fourth attribute considers the possibility of listening to participants' interests and wishes, to be considered in the further planning of courses. Also, this attribute is a binary one, i.e. either interests/wishes are considered or not. The final attribute is the cost of a course unit, ranging from 2 € to 9.5 €

For peer review only - http://bmjopen.bmj.com/site/about/guidelines.xhtml

in 1.5 € intervals. Besides a clear preference for lower costs per course unit, a priori we cannot hypothesise which levels will be preferred by women.

2.3.3 DCE questionnaire design and construction of choice sets

We constructed the final DCE experimental design following best practice guidelines [38]. The number of attributes and levels included in our DCE would generate 288 potential combinations ($2^2 \times 3 \times 4 \times 6$). The attribute costs per course unit entered were considered as a continuous variable in the experimental design. Owing to a decentralised administration of questionnaires, we were not able to implement a block design. As the number of possible choice scenarios is very large, we used a D-efficient design to select the most efficient combination of choice sets, while at the same time allowing the estimation of all the main effects [27, 39].

We employed SAS macros (SAS V.9.4) by Kuhfeld 2010 [40] to derive the optimal number of choice sets. The final efficient factorial fractional design (D-efficiency=5.8409, relative D-efficiency=73.0117, D-error=0.1712) consisted of eight unique choice tasks.

All attributes and level combinations were checked for plausibility. In order to test the reliability of the choices, we repeated one item (choice set 4), leading to nine choice sets included in the final DCE. Participants are asked to choose between course A and course B. To avoid increasing the cognitive effort for participants, we did not include any opt-out option.

2.3.4 The DCE questionnaire

The DCE will be included in the NU-BIG paper-based questionnaire, after a general questionnaire introduction and questions regarding health behaviour, health status, self-efficacy and integration and socio-demographic aspects. The DCE includes one page of detailed information on the content of the DCE. Participants are asked to choose from two options the one they prefer most, i.e. the one they would be less likely to reject. An example of a choice set is presented in Fig.1, and the full questionnaire in the original language is available in Appendix 5.

*** Insert Fig. 1 about here ***

2.4 Data analysis

2.4.1 Planned analyses

The included population will be described with respect to their socio-demographic characteristics drawing from the information collected in the NU-BIG questionnaire.

For peer review only - http://bmjopen.bmj.com/site/about/guidelines.xhtml

Respondents with missing information in more than half of the choice sets (> 4) will be excluded from the analysis. All other women will be eligible for the analysis.

In order to estimate the preference weight of each level within each attribute, we will analyse the DCE data using a conditional logit model. Thereby, we will calculate the relative importance of attributes, using an effects-coding procedure [41]. In a further step, we will express the preference weights as money equivalents (i.e. as the marginal rate of substitution between the attributes and the cost attribute), in order to compute the marginal willingness to pay for a change in the level of the attributes [31].

The target population of women who participate in BIG courses is likely to be a heterogeneous one, especially with regard to socio-demographic characteristics, which might impact preferences (e.g. presence of children in the household, migration background and linguistic barriers, religious affiliation, etc.). First, we will describe how the surveyed population (i.e. women who participate in BIG courses between summer 2022 and winter 2022/23) coincides with the intended target population of women in difficult life situations targeted by the BIG programme. Second, we will carry out a latent class analysis in order to explore the underlying heterogeneity and identify classes of women with different preferences. We will also analyse whether these classes will be different according to age, employment status, education, income, number of children, marital status, health status, migration status and religious affiliation (see Table 2 for details) [31]. All analyses will be conducted using R-studio [42].

2.4.2 Sample size calculation

All participating women at the active BIG sites will be offered the chance to participate in the NU-BIG survey. Therefore, we will have limited possibilities to influence the sample size for the DCE. Nevertheless, computing the minimum required size of the sample, also taking into account different response rates, is crucial for ensuring the feasibility of the DCE.

In order to compute the minimum sample size, we used the following rule of thumb [43, 44]:

$$n > \frac{1000\,c}{t \times a}$$

Here, n is the number of participants, c is the highest number of levels (in our case, c=4), t is the number of choice tasks (in our case, t=8) and a is the number of alternatives per choice task (in our case, a=2). In our case, this formula yields a minimum of 250 participants.

Based on participation prior to the Covid-19 pandemic, the number of participants in the BIG courses was estimated to be around 800. However, the context and participation are likely to be different now, so that a conservative approach for the estimated target group is necessary. Even

For peer review only - http://bmjopen.bmj.com/site/about/guidelines.xhtml

assuming a 50% reduction in the sample, or in the response rate, the sample size is still higher than the minimum suggestion of 250.

Table 2: Socio-demographic variables used in the latent class analysis

Socio-demographic characteristics	Measurement	Type of variable	Range/categories
Age	Self-reported	Continuous	Unknown
Employment status	Self-reported	Categorical	Employed, retired, unemployed, other
Education (highest qualification)	Self-reported	Categorical	No school diploma, mandatory school diploma, middle school diploma, high school diploma
Education (school years)	Self-reported	Continuous	Unknown
Household monthly income	Self-reported	Categorical	Below 500 €/month up to 6,000 € /month (in 500 € steps)
Number of children (below 14 years of age)	Self-reported	Continuous	Unknown
Living alone/with partner	Self-reported	Categorical	Living alone vs. with partner
Health status	Self-reported		1 (very good)–5 (very bad)
Migration status	Self-reported	Categorical	German born vs. foreign born
Religious affiliation	Self-reported	Categorical	Evangelical church, Catholic church, Islam, Judaism, Russian Orthodox church, no religious affiliation, other

2.5 Patient and public involvement

The BIG project is carried out using a ‘cooperative planning approach’ [19], where women from the target group are involved in the planning and implementation of physical activity courses. Furthermore, in the NU-BIG project, as a comprehensive evaluation of BIG, the point of view of women from the target group was always taken into account by regularly involving the group of ‘BIG experts’ in every step of the project for targeted feedback [21, 32].
Concretely, the idea for the DCE sparked from the meetings with the ‘BIG experts’ as a way of formally investigating the preferences of participating women. Both the ‘BIG experts’ and women from the target group of participants were involved in setting up the DCE in the pre-test phases. Their helpful critical feedback was used to further develop the instrument.

For peer review only - http://bmjopen.bmj.com/site/about/guidelines.xhtml

2.6 Strengths and limitations

The described DCE is the first to attempt to understand participants' preferences for structural characteristics of a community-based participatory research programme in the physical activity promotion field. Furthermore, it is the first DCE targeting the preferences of women in difficult life situations, a high-risk group when it comes to prevention efforts.

However, we anticipate the presence of some limitations. First, a DCE is based on the most important attributes relevant in the decision-making process of the actors. The trade-off between the number of attributes and the complexity of the choice task may result in the omission of attributes that are still relevant for some participants. Nonetheless, due to the thorough development and testing of the DCE, we expect that the experiment includes the most relevant attributes for the majority of participants.

Second, as recruitment of the experts and women participating in the pre-test were convenient samples, there might be a selection bias for these groups.

Third, we might face a further selection bias as we are not able to measure the preferences of women who left the courses. The preferences of this group might be different from those of women who are still participating in the course. Considering the preferences of women quitting the courses might have led to very interesting insights into the reasons for quitting and which course components might have helped to sustained participation. Therefore, the interpretation of our results is limited to the preferences of women who are still participating in the course.

3. Ethics and dissemination

The NU-BIG project was approved by the Ethics Commission of the Friedrich-Alexander-University of Erlangen-Nurnberg (Application no. 20-247_1-B). The DCE described in this protocol was included as part of the NU-BIG project and described in detail, also including recruitment for the pre-test with women from the target group.

All women participating in the pre-test read an extensive information sheet regarding the DCE and gave written informed consent and received an incentive (20 €). All participants in the final NU-BIG paper questionnaire will receive an information sheet and will be required to sign an informed consent form.

The DCE questionnaire and the relative data analysis will be carried out following the principles of good scientific practice [31, 38]. We will compile and attach to the final publication the

For peer review only - http://bmjopen.bmj.com/site/about/guidelines.xhtml

checklist for conjoint analysis applications in healthcare [33]. The results will not only be disseminated in academic journals and conferences, but also among participants groups and organising bodies, with the explicit aim of improving the courses offered by BIG at all sites.

For peer review only

For peer review only - http://bmjopen.bmj.com/site/about/guidelines.xhtml

Funding: This work was supported by the German Federal Ministry of Education and Research (grant no. 01EL20122D)

Author contributions: SP and ML identified the research question. SP, MV and VG planned the described DCE in all phases. AHM and RS worked as coordinators of the whole NU-BIG project and contributed to the organisation of the preparatory steps in the DCE. SP, AHM, AS and SL carried out the necessary steps for the identification of potential attributes and interviewed the experts. SP and AS carried out the pre-test with participants. SP wrote a first draft of the manuscript, and all authors read, commented on and approved the manuscript. KAO, HZ, AT, AHM and RH were responsible for the funding application and work as coordinators for the NU-BIG evaluation. They contributed expert knowledge to the identification of the research question, the setting up of the DCE and the final manuscript.

SP – Sara Pedron
ML – Michael Laxy
MV – Markus Vomhof
VG – Veronika Gontscharuk
AHM – Annika Herbert-Maul
AS – Alexandra Sauter
SL – Stephanie Linder
RS – Raluca Sommer
KAO – Karim Abu-Omar
HZ – Heiko Ziemainz
AT – Ansgar Thiel
RH – Rolf Holle

Competing interests statement: the authors declare no competing interests.

Data Sharing statement: after publication of the main analysis paper, all data will be made available upon request and after anonymisation to researchers who provide a sound analysis plan to replicate our results.

For peer review only

References

1. Gidlow C, Johnston LH, Crone D, Ellis N, James D. A systematic review of the relationship between socio-economic position and physical activity. Health Educ J. 2016;65:338–67.

2. Beenackers MA, Kamphuis CB, Giskes K, Brug J, Kunst AE, Burdorf A, et al. Socioeconomic inequalities in occupational, leisure-time, and transport related physical activity among European adults: A systematic review. Int J Behav Nutr Phys Act. 2012;9:1–23.

3. Abu-Omar K, Messing S, Sarshar M, Gelius P, Ferschl S, Finger J, et al. Sociodemographic correlates of physical activity and sport among adults in Germany: 1997–2018. Ger J Exerc Sport Res. 2021;51:170–82.

4. Petrovic D, de Mestral C, Bochud M, Bartley M, Kivimäki M, Vineis P, et al. The contribution of health behaviors to socioeconomic inequalities in health: A systematic review. Prev Med (Baltim). 2018;113:15–31.

5. Ben-Shlomo Y, Kuh D. A life course approach to chronic disease epidemiology: conceptual models, empirical challenges and interdisciplinary perspectives. Int J Epidemiol. 2002;31:285–93.

6. Benjamin K, Donnelly TT. Barriers and facilitators influencing the physical activity of Arabic adults: A literature review. Avicenna. 2013;2013:8.

7. Bhatnagar P, Foster C, Shaw A. Barriers and facilitators to physical activity in second-generation British Indian women: A qualitative study. PLoS One. 2021;16:e0259248.

8. Siefken K, Schofield G, Schulenkorf N. Laefstael Jenses: an investigation of barriers and facilitators for healthy lifestyles of women in an urban Pacific Island context. J Phys Act Health. 2014;11:30–7.

9. Frahsa A, Streber A, Wolff AR, Rütten A. Capabilities for physical activity by Turkish- and Russian-speaking immigrants aged 65 years and older in Germany: A qualitative study. J Aging Phys Act. 2020;28:567–79.

10. Withall J, Jago R, Fox KR. Why some do but most don't. Barriers and enablers to engaging low-income groups in physical activity programmes: A mixed methods study. BMC Public Health. 2011;11:1–13.

For peer review only

For peer review only - http://bmjopen.bmj.com/site/about/guidelines.xhtml

11. Rütten A, Abu-Omar K, Adlwarth W, Meierjürgen R. Bewegungsarme lebensstile – Zur klassifizierung unterschiedlicher zielgruppen für eine gesundheitsförderliche körperliche aktivierung. Gesundheitswesen. 2007;69:393–400.

12. Hoebel J, Finger JD, Kuntz B, Kroll LE, Manz K, Lange C, et al. Changing educational inequalities in sporting inactivity among adults in Germany: a trend study from 2003 to 2012. BMC Public Health. 2017;17:1–10.

13. Krug S, Jordan S, Mensink GBM, Müters S, Finger J, Lampert T. Körperliche Aktivität: Ergebnisse der Studie zur Gesundheit Erwachsener in Deutschland (DEGS1). Bundesgesundheitsblatt - Gesundheitsforsch - Gesundheitsschutz. 2013;56:765–71.

14. Robert-Koch-Institut. Gesundheitliche Lage der Frauen in Deutschland. Gesundheitsberichterstattung des Bundes Gem getragen von RKI und Destatis. 2020.

15. Rütten A, Schow D, Breda J, Galea G, Kahlmeier S, Oppert JM, et al. Three types of scientific evidence to inform physical activity policy: results from a comparative scoping review. Int J Public Health. 2016;61:553–63.

16. Rutter H, Bes-Rastrollo M, De Henauw S, Lahti-Koski M, Lehtinen-Jacks S, Mullerova D, et al. Balancing upstream and downstream measures to tackle the obesity epidemic: A position statement from the European Association for the Study of Obesity. Obes Facts. 2017;10:61–3.

17. Rütten A, Abu-Omar K, Frahsa A, Morgan A. Assets for policy making in health promotion: Overcoming political barriers inhibiting women in difficult life situations to access sport facilities – ScienceDirect. Soc Sci Med. 2009;69:1667–73.

18. Leung MW, Yen IH, Minkler M. Community based participatory research: a promising approach for increasing epidemiology's relevance in the 21st century. Int J Epidemiol. 2004;33:499–506.

19. Rütten A, Röger U, Abu-Omar K, Frahsa A. Empowerment von Frauen in Sozial benachteiligten Lebenslagen: Das BIG-projekt. Gesundheitswesen. 2008;70:742–7.

20. Röger U, Rütten A, Frahsa A, Abu-Omar K, Morgan A. Differences in individual empowerment outcomes of socially disadvantaged women: Effects of mode of participation and structural changes in a physical activity promotion program. Int J Public Health. 2011;56:465–73.

For peer review only - http://bmjopen.bmj.com/site/about/guidelines.xhtml

21. Herbert-Maul A, Abu-Omar K, Frahsa A, Streber A, Reimers AK. Transferring a community-based participatory research project to promote physical activity among socially disadvantaged women—experiences from 15 Years of BIG. Front Public Health. 2020;8:546.

22. Haldane V, Chuah FLH, Srivastava A, Singh SR, Koh GCH, Seng CK, et al. Community participation in health services development, implementation, and evaluation: A systematic review of empowerment, health, community, and process outcomes. PLoS One. 2019;14:e0216112.

23. O'Mara-Eves A, Brunton G, Oliver S, Kavanagh J, Jamal F, Thomas J. The effectiveness of community engagement in public health interventions for disadvantaged groups: A meta-analysis. BMC Public Health. 2015;15:1–23.

24. Rütten A, Gelius P. Building policy capacities: an interactive approach for linking knowledge to action in health promotion. Health Promot Int. 2014;29:569–82.

25. Gelius PC, Jansen M, King AC. Cooperative planning and its utilization in German physical activity promotion: a brief introduction. Health Promot Int. 2021;36 Supplement 2:ii1–7.

26. Sanz-Remacha M, García-González L, Sevil Serrano J, Aibar Solana A. A qualitative evaluation of a community-based intervention on health-related behaviors in disadvantaged women. Res Q Exerc Sport. 2022. https://doi.org/10.1080/02701367.2021.1971149.

27. Bekker-Grob EW de, Ryan M, Gerard K. Discrete choice experiments in health economics: a review of the literature. Health Econ. 2012;21:145–72.

28. Lancaster KJ. A new approach to consumer theory. J Polit Econ. 1966;74:132–57.

29. Louviere JJ, Hensher DA, Swait JD, Adamowicz W. Stated Choice Methods: Analysis and Applications. Cambridge University Press; 2000.

30. Lancsar E, Savage E. Deriving welfare measures from discrete choice experiments: inconsistency between current methods and random utility and welfare theory. Health Econ. 2004;13:901–7.

31. Hauber AB, González JM, Groothuis-Oudshoorn CGM, Prior T, Marshall DA, Cunningham C, et al. Statistical methods for the analysis of discrete choice experiments: A report of the ISPOR Conjoint Analysis Good Research Practices Task Force. Value Health. 2016;19:300–15.

For peer review only - http://bmjopen.bmj.com/site/about/guidelines.xhtml

32. Abu-Omar K, Ziemainz H, Loss J, Laxy M, Holle R, Thiel A, et al. The long-term public health impact of a community-based participatory research project for health promotion among socially disadvantaged women—a case study protocol. Front Public Health. 2021;9:317.

33. Bridges JFP, Hauber AB, Marshall D, Lloyd A, Prosser LA, Regier DA, et al. Conjoint analysis applications in health – A checklist: A report of the ISPOR Good Research Practices for Conjoint Analysis Task Force. Value Health. 2011;14:403–13.

34. Telser H. Nutzenmessung im Gesundheitswesen: Die Methode der Discrete-Choice-Experimente. Hamburg: Verlag Dr. Kovač; 2002.

35. Drennan J. Cognitive interviewing: verbal data in the design and pretesting of questionnaires. J Adv Nurs. 2003;42:57–63.

36. Charters E. The use of think-aloud methods in qualitative research. An introduction to think-aloud methods. Brock Educ J. 2003;12.

37. Clarke V, Braun V. Thematic analysis. Encycl Crit Psychol. 2014;:1947–52.

38. Johnson FR, Lancsar E, Marshall D, Kilambi V, Mühlbacher A, Regier DA, et al. Constructing experimental designs for discrete-choice experiments: Report of the ISPOR Conjoint Analysis Experimental Design Good Research Practices Task Force. Value Health. 2013;16:3–13.

39. Lancsar E, Louviere J. Conducting discrete choice experiments to inform healthcare decision making: A user's guide. Pharmacoeconomics. 2008;26:661–77.

40. Kuhfeld WF. Marketing Research Methods in SAS: Experimental Design, Choice, Conjoint, and Graphical Techniques. MR-2010. 2010.

41. Bech M, Gyrd-Hansen D. Effects coding in discrete choice experiments. Health Econ. 2005;14:1079–83.

42. RStudio Team. RStudio: Integrated Development for R. 2020.

43. de Bekker-Grob EW, Donkers B, Jonker MF, Stolk EA. Sample size requirements for discrete-choice experiments in healthcare: a practical guide. Patient. 2015;8:373–84.

44. Orme BK. Getting started with conjoint analysis: strategies for product design and pricing research. 2nd edn. Madison, WI: Research Publishers; 2010.

For peer review only

For peer review only

For peer review only - http://bmjopen.bmj.com/site/about/guidelines.xhtml

Figure legends

Fig. 1: Example of choice set

Note: own translation from German.

For peer review only

For peer review only - http://bmjopen.bmj.com/site/about/guidelines.xhtml

	Class A		Class B
**Course time**	Morning (10 am - 12 pm)		Early morning (8 - 10 am)
**Travel time to the BIG course**	Maximum 20 minutes		Maximum 10 minutes
**Additional activities by BIG**	No additional social activity is organised		Additional social activities are organised
**Consideration of wishes and interests for the further planning of courses**	Interests/wishes are asked/considered		Interests/wishes are not asked/considered
**Cost per course unit**	8.00 €		5.00 €
**Which class do you prefer? (Please tick the corresponding box)**	☐		☐

Fig. 1: Example of choice set

274x96mm (120 x 120 DPI)

Preferences of women in difficult life situations for a physical activity program: protocol of a discrete choice experiment in the NU-BIG project

Sara Pedron, Annika Herbert-Maul, Alexandra, Stephanie Linder, Raluca Sommer, Markus Vomhof, Veronika Gontscharuk, Karim Abu-Omar, Ansgar, Heiko Ziemainz, Rolf Holle & Michael Laxy

Supplementary material

For peer review only

Appendix 1: course description in each BIG-site

Table A.1: description of courses in active sites in 2019

	time unit	Number of participants	Number of fitness courses	Number of water courses	Additional offers	Additional social activities	Planning & participation	mean number of participants per course
Erlangen	year	695	57	13	women-only bathing time, additional sport activities (dancing 2x per year, archery, climbing,…)	women´s breakfast (8x year), healthy diet course (2x year), arabic cuisine tasting (1x year)	Planning meeting (2x year)	10
Regensburg	I	166	34	3	women-only bathing time, biking course			10
	II	132						
	III	88						
Bayreuth	I	127	18	8	biking course			13
	II	112						
	III	108						
Straubing		144	10	0	boxing	integration course		14
Berlin (Treptow-Köpenick)		4	1					4
Nürnberg	I	63	0	3				21
	II	61	0	3				
	III	81	0	4				
Neustadt a.d. Aisch		20		2				10

Note: the table shows the number of participants in each active site in 2019, either for the whole years or for different course seasons (I, II or III).

For peer review only - http://bmjopen.bmj.com/site/about/guidelines.xhtml

Appendix 2: Systematic literature search protocol and results

Goal: finding studies which evaluated individual preferences for lifestyle programs/physical activity programs

Search in PubMed:

(physical activity[Title/Abstract]) AND (discrete choice[Title/Abstract])

((physical activity[Title/Abstract]) AND (conjoint analysis[Title/Abstract])

(lifestyle[Title/Abstract]) AND (discrete choice[Title/Abstract])

(coaching[Title/Abstract]) AND (discrete choice[Title/Abstract])

(coaching[Title/Abstract]) AND (conjoint analysis[Title/Abstract])

(sport[Title/Abstract]) AND (discrete choice[Title/Abstract])

(sport[Title/Abstract]) AND (conjoint analysis[Title/Abstract])

(empowerment[Title/Abstract]) AND (discrete choice[Title/Abstract])

(empowerment[Title/Abstract]) AND (conjoint analysis[Title/Abstract])

(exercise[Title/Abstract]) AND (discrete choice[Title/Abstract])

(exercise[Title/Abstract]) AND (conjoint analysis[Title/Abstract])

The two search phrases in gray did not lead to any result.

For peer review only

For peer review only - http://bmjopen.bmj.com/site/about/guidelines.xhtml

Flowchart of the retrieved studies and screening process

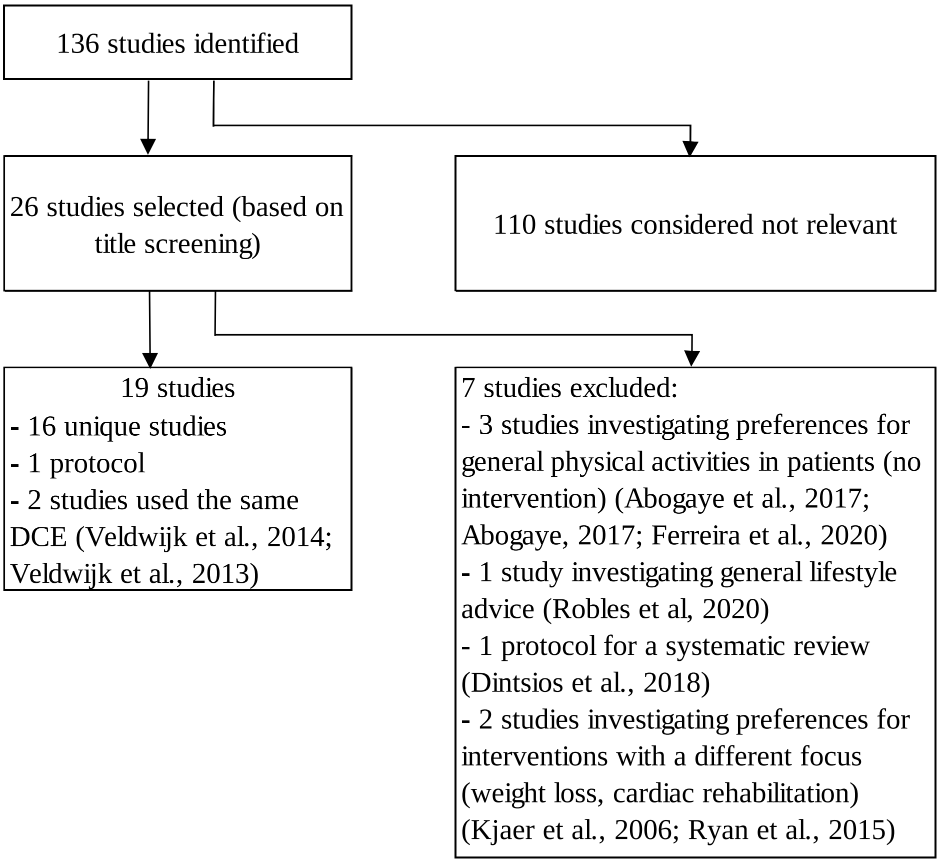

General overview of the retrieved studies

The search led to 18 studies and one protocol of one of the studies (Pinto et al., 2017). Two studies (Veldwijk et al., 2013; Veldwijk et al., 2014) used the same DCE, so we will refer to only one of them for simplicity. The studies focused on different topics but were all concerned in deploying DCE techniques to understand preferences for a physical activity intervention (sometimes labeled as "lifestyle intervention", including also diet). They dealt with the following topics:

- Lifestyle intervention for:
  - Diabetes prevention (Owen et al., 2010)
  - Stroke survivors (Geidl et al., 2018)
  - Knee osteoarthritis (Pinto et al., 2017; Pinto et al., 2019) [Study + Protocol]
  - Parkinson´s disease (Paul et al., 2021)
  - Diabetes patients (Salampessy et al., 2015; Sommer et al., 2020; Veldwijk et al., 2014, Veldwijk et al., 2013, Ramirez & Beale, 2016; van Gils et al., 2011) [two studies by Veldwijk using the same DCE]
  - Older adults (Brown, 2009)
- Focus on outcomes of health promotion interventions (Alayli-Goebbels, 2013)
- Focus on goals of health promotion interventions (Benning, 2020)
- Focus on financial incentives for physical activity (Farooqui, 2014; Giles, 2016; Molema, 2019, Wanders, 2014, Matsushita, 2017)

For peer review only - http://bmjopen.bmj.com/site/about/guidelines.xhtml

Systematic extraction of attributes and levels

We collected all attributes and levels from the selected studies. We eliminated those who were specific of the context analyzed and not transferrable to our context (diet related items, items related to a specific disease). Furthermore, we excluded those that were potentially relevant for a general physical activity intervention but were not applicable to our context (mixed groups including men, exercising alone/at home, schedule and goal setting by coach/physician, monetary interventions, goal setting, consultations with coach or physician, etc.). The remaining attributes are presented in Table A.2.

For peer review only

For peer review only - http://bmjopen.bmj.com/site/about/guidelines.xhtml

Table A.2: attributes and levels retrieved via systematic literature search

Attribute	Levels	Study
**Exercise frequency and duration**		
Exercise structure	Daily 30 min<br>daily 45 min<br>weekly 3h<br>weekly 5h	Owen et al., 2020
Frequency	1-2 sessions per week<br>3 sessions per week<br>4-5 sessions per week	Geidl et al., 2018
Duration	20-30 minutes per session<br>45-60 minutes per session	Geidl et al., 2018
Number of 45-minute exercise sessions per week	1x45-minute session per week<br>2x45-minute sessions per week<br>3x45-minute sessions per week<br>4x45-minute sessions per week	Paul et al., 2021
Total time required	12 hours/week<br>10 hours/week<br>8 hours/week<br>6 hours/week	Sommer et al., 2020
Time spent on the program	2.5 hours per week<br>4 hours per week	van Gils et al., 2011
Number of walking days per week for 3 months	2 days<br>3 days<br>5 days	Brown et al., 2009
walking time perday	20 min<br>30 min<br>45 min<br>75 min	Brown et al., 2009
time per week	0 h<br>2 h<br>4 h<br>6 h	Alayli-Goebbels et al., 201
Exercise per week	2 h<br>4 h<br>6 h	Benning et al., 2020
Average number of sessions required per week	1 session<br>2 sessions	Farooqui et al., 2014
Time per physical activity occasion	Low<br>Medium<br>High	Pinto et al., 2019

For peer review only - http://bmjopen.bmj.com/site/about/guidelines.xhtml

Attribute	Levels	Study
**Context**		
Group activities	No group activities<br>Group activities	Sommer et al., 2020
**Type of exercise**		
Type of exercise	Endurance<br>Muscular strength<br>Neuromuscular and flexibility<br>Mixed program	Geidl et al., 2018
Intensity	Light activity<br>Moderate activity<br>Vigorous activity	Geidl et al., 2018
Physical activity effort	Low<br>Medium<br>High	Pinto et al., 2019
Sports activity	Walking/cycling<br>Fitness (treadmill, rowing machine, bicycle)	van Gils et al., 2011
Exercise type	Dance program (eg, tango or waltz/foxtrot or Irish set dancing)<br>Balance exercise program (exercises to challenge balance, performed while standing, may include Tai-chi)<br>Muscle strength exercise program (exercises aimed at increasing muscle strength, eg, resistance exercises using free weights, exercises using your body weight, weight machines, weighted vests, TheraBand)<br>Aerobic exercise program (exercises to challenge the heart and lungs and make you puff, eg, fast walking, jogging, running, cycling)<br>Walking exercise program (eg, walking indoors and/or outdoors, treadmill walking, walking using visual and/or auditory cues)<br>Multimodal exercise program (including at least 2 of the following exercises): balance exercises, strength exercises, aerobic exercises, walking exercises	Paul et al., 2021
**Outcomes & goals**		
Outcomes	0.25 kg, no body change<br>0.5kg no body change<br>0.5kg body change<br>0.25 kg, body change	Owen et al., 2020
Expected outcome	No weight loss but feeling fitter 0<br>Weight loss of 5 kilograms and feeling fitter 5<br>Weight loss of 10 kilograms and feeling fitter 10	Wanders et al., 2014

For peer review only - http://bmjopen.bmj.com/site/about/guidelines.xhtml

Attribute	Levels	Study
Expected outcomes	No weights loss (reference) but you feel fitter<br>A weight loss of 5 kg and you feel fitter<br>A weight loss of 10 kg and you feel fitter	Salampessy et al., 2015
Expected outcomes	No weight loss but feeling more healthy<br>5 kilograms of weight lost and feeling more healthy<br>10 kilograms of weight lost and feeling more healthy	Veldwijk et al., 2013
expected weight loss	5 kg<br>10 kg	Benning et al., 2020
Goal	Looking better by losing weight<br>Increasing life expectancy by losing weight	Benning et al., 2020
clothing size above ideal	0 sizes<br>1/2 size<br>1 size<br>2 sizes	Alayli-Goebbels et al., 2013
Health benefits	Low<br>Medium<br>High	Pinto et al., 2019
Effect on your overall feeling of wellbeing	You will experience no improvement in overall feeling of wellbeing<br>You will experience small improvement in overall feeling of wellbeing<br>You will experience moderate improvement in overall feeling of wellbeing<br>You will experience large improvement in overall feeling of wellbeing	Paul et al., 2021
Future health state value	0.5<br>0.7<br>0.8<br>1	Alayli-Goebbels et al., 2013
start point of future health state	in 2 yrs<br>in 5 yrs<br>in 10 yrs<br>in 20 yrs	Alayli-Goebbels et al., 2013
life expectancy	80 yrs<br>81 yrs<br>82 yrs<br>83 yrs	Alayli-Goebbels et al., 2013
days without sufficient relaxation	0-1 per week<br>2-3 per week<br>4-5 per week<br>6-7 per week	Alayli-Goebbels et al., 2013

Attribute	Levels	Study
endurance	poor<br>modest<br>good<br>vey good	Alayli-Goebbels et al., 2013
experienced control over lifestyle choices	little<br>some<br>moderate<br>much	Alayli-Goebbels et al., 2013
Enjoyment	Low<br>Medium<br>High	Pinto et al., 2019
lifestyle improvemens of potential partner/children	yes<br>no	Alayli-Goebbels et al., 2013
**Schedule**		
Convenience (how well activity fits into your schedule)	Low<br>Medium<br>High	Pinto et al., 2019
**Costs**		
Enrollment fee	Free<br>$20<br>$50	Farooqui et al., 2014
Additional cost of gym/healthier food options	no additional cost<br>AU$ 15/week	Owen et al., 2020
Monthly cost	Low<br>Medium<br>High	Pinto et al., 2019
Out of pocket cost (in AU$) including travel costs per session	Out of pocket cost = $0 per session<br>Out of pocket cost = $10 per session<br>Out of pocket cost = $25 per session<br>Out of pocket cost = $50 per session<br>Out of pocket cost = $100 per session<br>Out of pocket cost = $150 per session	Paul et al., 2021
OOP costs	OOP costs of €75 per 3–6 months<br>OOP costs of €150 per 3–6 months<br>OOP costs of €225 per 3–6 months	Salampessy et al., 2015
Out-of-pocket costs	75 euro per year<br>150 euro per year<br>225 euro per year	Veldwijk et al., 2013
money per month	0 €<br>25 €<br>50 €<br>100 €	Alayli-Goebbels et al., 2013

For peer review only - http://bmjopen.bmj.com/site/about/guidelines.xhtml

Attribute	Levels	Study
Cost to travel to sessions	3 sessions<br>Free<br>$2<br>$5	Farooqui et al., 2014
**Travel time & location**		
Exercise location	In the local neighbourhood (eg, local parks, streets or outdoor public spaces)<br>At a hospital or health centre/practice<br>At a community centre or facility (eg, gym, community hall)<br>At multiple locations including home<br>At multiple locations excluding home	Paul et al., 2021
Travel time per exercise session	Less than 5 minutes each way travel time<br>5 minutes each way travel time<br>10 minutes each way travel time<br>15 minutes each way travel time<br>30 minutes each way travel time<br>60 minutes or more each way travel time	Paul et al., 2021
Time to travel to sessions	15 minutes<br>25 minutes<br>30 minutes<br>45 minutes	Farooqui et al., 2014
**Support**		
Amount of supervision	All of the exercise is supervised<br>Some of the exercise is supervised<br>None of the exercise is supervised	Paul et al., 2021
**Other**		
other information provided	no other information<br>written leaflet on benefits of activity and ways to be more active<br>face-to-face discussions on benefits of activity and ways to be more active<br>written leaflet and face-to-face idscussion on benefits of activity and ways to be more active	Giles et al., 2016

For peer review only - http://bmjopen.bmj.com/site/about/guidelines.xhtml

Appendix 3: further definition of attributes and levels based on experts feedback

In the meeting with the expert, 10 attributes were presented:

- *Price per course unit*
- *Course times*
- *Travel time to hall/course*
- *Additional social activities*
- *Additional course content*
- *Participation in organization*
- *Course registration*
- *Group composition*
- *Presence of childcare*
- *Course duration (in minutes)*

The discussion led to a reformulation of two attributes and the identification of an additional one. The additional attribute identified was "flexible intensity of the course" (i.e. if the intensity of the exercises within a course could be flexibly adjusted or not). The two attributes that were reformulated were:

- "course registration" (i.e. if all costs for the whole course must be paid in advance or if each appointment can be paid separately) was substituted by the attribute "trial lecture" (i.e. if a trial lecture is offered or not).
- "group composition" (i.e. women from the same social background/migration background vs mixed group) was substituted by an attribute specifically targeting respect of religious standards (i.e. framework: the hall is visible from the outside or not).

Based on the insights gained during the consultation with the experts, we performed a selection of those attributes which respected the following characteristics:

- Relevant for the overall utility
- Influenceable
- Close to reality (in the BIG program)
- Substitutive relation
- No dominance

Based on the insights from the experts we considered *presence of childcare* and *presence of trial lessons* as dominant criteria for the decision. These attributes, together with the presence of a female coach were considered as fix characteristics for the DCE.

According to the experts, the attribute "*course duration (in minutes)*" was distributed between 60 and 90 minutes for almost all offered courses. Given the limited variation, we decided to discard this attribute and fix it at 60 Minutes along the previously mentioned course characteristics.

The attribute "*course registration*" (paying everything at the beginning or paying each lesson at a time) was perceived as not relevant by the experts. Therefore we decided to exclude this attribute altogether.

The attribute "*flexible intensity (yes or no)*" was also deemed unimportant for the present DCE, since basically in all courses offered the intensity can be flexibly adjusted. Women also have the possibility of visiting a trial lesson and receive individual advice regarding their fitness level. This information will be included also in the fix course components.

The attribute "*framework (hall is visible from the outside yes or no)*" was deemed as a dominant criterion for some women (especially Muslim women) and not relevant for some other women. In both cases the attribute should not be included in the DCE. We therefore decided to fix this attribute as well.

The remaining attributes were included in the DCE. These are:

For peer review only - http://bmjopen.bmj.com/site/about/guidelines.xhtml

- *Price per course unit*
- *Course times*
- *Travel time to hall/course*
- *Additional activities (social + content)*
- *Participation in organization*

The attributes "*additional social activities*" and "*additional course content*" were merged into one attribute for the following reasons:

- The attribute additional course content was not clearly formulated and led experts to different interpretations/questions of what it means (e.g. additional lectures or additional content in the same appointment as the sport course? Etc.)
- At the moment in most BIG courses additional social activities include also the informal exchange on other topics (healthy diet, mental health, integration, help with translations,…) so that the two additional activities (social and content) largely overlap at the moment

The final attributes and levels included are (original language):

Attribute	Levels
**Kurszeiten**	Morgens (8-10 Uhr)<br>Vormittags (10-12 Uhr)<br>Nachmittags (16-18 Uhr)<br>Abends (18-20 Uhr)
**Dauer Geh-/Fahrweg bis zum BIG-Kurs**	Maximal 10 Minuten<br>Maximal 20 Minuten<br>Maximal 30 Minuten
**zusätzliche soziale Aktivitäten im Rahmen von BIG organisiert**	Keine zusätzliche Aktivität wird organisiert<br>Zusätzliche soziale Aktivitäten werden organisiert
**Berücksichtigung von Interessen und Wünsche bei der weiteren Kursplanung**	Interessen/Wünsche werden nicht abgefragt/nicht berücksichtigt<br>Interessen/Wünsche werden abgefragt/berücksichtigt
**Kurskosten pro Kurseinheit**	0 €<br>1 €<br>2 €<br>3 €<br>4 €<br>5 €

For peer review only - http://bmjopen.bmj.com/site/about/guidelines.xhtml

Appendix 4: list of attributes and levels in the original language (German)

Attribute	Levels
**Kurszeiten**	Morgens (8-10 Uhr)
	Vormittags (10-12 Uhr)
	Nachmittags (16-18 Uhr)
	Abends (18-20 Uhr)
**Dauer Geh-/Fahrweg bis zum BIG-Kurs**	Maximal 10 Minuten
	Maximal 20 Minuten
	Maximal 30 Minuten
**Zusätzliche soziale Aktivitäten im Rahmen von BIG organisiert**	Keine zusätzliche Aktivität
	Zusätzliche soziale Aktivitäten
**Berücksichtigung von Interessen und Wünsche bei der weiteren Kursplanung**	Interessen/Wünsche werden nicht berücksichtigt
	Interesse/Wünsche werden berücksichtigt
**Kurskosten pro Kurseinheit**	2,0 €
	3,5 €
	5,0 €
	6,5 €
	8,0 €
	9,5 €

For peer review only - http://bmjopen.bmj.com/site/about/guidelines.xhtml

Appendix 5: final DCE questionnaire (original language)

For peer review only

For peer review only - http://bmjopen.bmj.com/site/about/guidelines.xhtml

BIG-Projekt

Fragebogen

Liebe Teilnehmerin,

„BIG“ steht für „Bewegung als Investition in Gesundheit“. Bei BIG werden Sport- und Bewegungskurse für Frauen angeboten. Bei diesen Kursen ist es sehr wichtig, dass jede interessierte Frau teilnehmen kann. Deswegen sollen die Kurse ihren Wünschen, Bedürfnissen und Interessen so gut wie möglich entsprechen. Vielleicht wussten Sie das noch nicht, aber auch der Kurs an dem Sie teilnehmen, ist aus dem BIG-Projekt entstanden.

Um BIG weiter zu verbessern, möchten wir gerne mehr über Ihre Wünsche und Interessen erfahren. Außerdem interessiert uns, ob BIG bei Ihnen eine Wirkung erzielt hat, z.B. auf Ihre Gesundheit. In dem Fragebogen werden deshalb Fragen zu den Kursen und Ihrer persönlichen Situation gestellt. Wir bitten Sie diese Fragen zu beantworten.

Wir bedanken uns herzlich für Ihre Unterstützung.

Ihr BIG-Team.

For peer review only

wichtige Informationen:

- Bitte beantworten Sie die Fragen, wie es Ihrer persönlichen Meinung entspricht.
- Es gibt keine richtigen oder falschen Antworten.
- Ihre Angaben sind anonym. Es sind keine Rückschlüsse auf Ihre Person möglich.
- Wir versichern Ihnen, dass Ihre Angaben vertraulich behandelt werden.
- Ihre Teilnahme ist freiwillig.
- Sollten Sie eine Frage nicht verstehen oder beantworten wollen, lassen Sie diese aus.

For peer review only - http://bmjopen.bmj.com/site/about/guidelines.xhtml

K. Kurspräferenzen

Wir möchten in diesem Teil herausfinden, welche Wünsche Sie haben, wie BIG-Sportkurse gestaltet sein sollen. Ziel ist es, Kurse anzubieten, an denen Sie auch weiterhin gerne teilnehmen.

In den folgenden Fragen werden jeweils zwei Kurse gegenübergestellt. Die Kurse unterscheiden sich in den Kurskosten, den Kurszeiten, der Dauer des Geh-/Fahrwegs, der Berücksichtigung von Interessen und zusätzlichen sozialen Aktivitäten.

Alle BIG-Sportkurse dauern ungefähr 60 Minuten und finden in Räumlichkeiten statt, die von außen nicht einsehbar sind. Sie können alle BIG-Sportkurse in einer Schnupperstunde testen, bevor Sie sich für den Kurs entscheiden. In allen BIG-Sportkursen besteht die Möglichkeit einer Kinderbetreuung. Ein Kurs hat jeweils 10 Termine. Die Termine finden unter der Woche statt.

Bitte kreuzen Sie jeweils an, ob Sie lieber an Kurs A oder Kurs B teilnehmen würden.

Sie können entweder Kurs A oder Kurs B wählen. Sie können nicht einen Kurs aus einzelnen Eigenschaften zusammenstellen. Bitte wählen Sie immer den Kurs, den Sie bevorzugen beziehungsweise den Kurs, den Sie weniger ablehnen.

Hier finden Sie eine Beschreibung der Eigenschaften, welche die zwei Kurse haben können:

Kurszeiten: Wann der Kurs stattfindet. Mögliche Uhrzeiten sind morgens (8-10 Uhr), vormittags (11-12 Uhr), nachmittags (16-18 Uhr) und abends (18-20 Uhr).

Dauer Fahrweg: Wie lange Sie zu der Halle/dem Kursort brauchen (zu Fuß oder mit dem für Sie bequemsten Verkehrsmittel): die Möglichkeiten sind 10, 20 oder 30 Minuten.

Zusätzliche soziale Aktivitäten im Rahmen von BIG organisiert: Ob zusätzliche soziale Aktivitäten von BIG organisiert werden oder nicht (z.B. Frauenfrühstück oder gemeinsame Kochabende einmal im Monat).

Berücksichtigung von Interessen und Wünsche: Ob Ihre persönlichen Interessen oder Wünsche bei der Planung von Kursen abgefragt und (wenn möglich) berücksichtigt werden oder nicht.

Kurskosten pro Termin: Wie viel Sie für jeden Kurstermin zahlen müssen (in €). In unserem Fragebogen liegt der Preis der Kurse zwischen 2,00 und 9,50 Euro pro Termin.

For peer review only - http://bmjopen.bmj.com/site/about/guidelines.xhtml

Bitte wählen Sie den Kurs aus, der Ihnen mehr zusagt. Bitte wählen Sie immer einen Kurs.

L1. Auswahlmöglichkeit	Kurs A	Kurs B
**Kurszeiten**	Vormittags (10-12 Uhr)	Morgens (8-10 Uhr)
**Dauer Geh-/Fahrweg bis zum BIG-Kurs**	Maximal 20 Minuten	Maximal 10 Minuten
**Zusätzliche soziale Aktivitäten im Rahmen von BIG organisiert**	Keine zusätzliche Aktivität	Zusätzliche soziale Aktivitäten
**Berücksichtigung von Interessen und Wünsche bei der weiteren Kursplanung**	Interessen/Wünsche werden berücksichtigt	Interessen/Wünsche werden nicht berücksichtigt
**Kurskosten (pro Termin)**	8,00 €	5,00 €
**Welchen Kurs bevorzugen Sie? (Kreuzen Sie bitte das entsprechende Kästchen an)**	☐	☐

Bitte wählen Sie den Kurs aus, der Ihnen mehr zusagt. Bitte wählen Sie immer einen Kurs.

L2. Auswahlmöglichkeit	Kurs A	Kurs B
**Kurszeiten**	Abends (18-20 Uhr)	Nachmittags (16-18 Uhr)
**Dauer Geh-/Fahrweg bis zum BIG-Kurs**	Maximal 30 Minuten	Maximal 20 Minuten
**Zusätzliche soziale Aktivitäten im Rahmen von BIG organisiert**	Keine zusätzliche Aktivität	Zusätzliche soziale Aktivitäten
**Berücksichtigung von Interessen und Wünsche bei der weiteren Kursplanung**	Interessen/Wünsche werden berücksichtigt	Interessen/Wünsche werden nicht berücksichtigt
**Kurskosten (pro Termin)**	5,00 €	9,50 €
**Welchen Kurs bevorzugen Sie? (Kreuzen Sie bitte das entsprechende Kästchen an)**	☐	☐

Bitte wählen Sie den Kurs aus, der Ihnen mehr zusagt. Bitte wählen Sie immer einen Kurs.

L3. Auswahlmöglichkeit	Kurs A	Kurs B
**Kurszeiten**	Abends (18-20 Uhr)	Vormittags (10-12 Uhr)
**Dauer Geh-/Fahrweg bis zum BIG-Kurs**	Maximal 20 Minuten	Maximal 10 Minuten
**Zusätzliche soziale Aktivitäten im Rahmen von BIG organisiert**	Keine zusätzliche Aktivität	Zusätzliche soziale Aktivitäten
**Berücksichtigung von Interessen und Wünsche bei der weiteren Kursplanung**	Interessen/Wünsche werden nicht berücksichtigt	Interessen/Wünsche werden berücksichtigt
**Kurskosten (pro Termin)**	3,50 €	9,50 €
**Welchen Kurs bevorzugen Sie? (Kreuzen Sie bitte das entsprechende Kästchen an)**	☐	☐

For peer review only - http://bmjopen.bmj.com/site/about/guidelines.xhtml

Bitte wählen Sie den Kurs aus, der Ihnen mehr zusagt. Bitte wählen Sie immer einen Kurs.

L4. Auswahlmöglichkeit	Kurs A	Kurs B
**Kurszeiten**	Morgens (8-10 Uhr)	Vormittags (10-12 Uhr)
**Dauer Geh-/Fahrweg bis zum BIG-Kurs**	Maximal 30 Minuten	Maximal 10 Minuten
**Zusätzliche soziale Aktivitäten im Rahmen von BIG organisiert**	Keine zusätzliche Aktivität	Keine zusätzliche Aktivität
**Berücksichtigung von Interessen und Wünsche bei der weiteren Kursplanung**	Interessen/Wünsche werden berücksichtigt	Interessen/Wünsche werden nicht berücksichtigt
**Kurskosten (pro Termin)**	6,50 €	5,00 €
**Welchen Kurs bevorzugen Sie? (Kreuzen Sie bitte das entsprechende Kästchen an)**	☐	☐

Bitte wählen Sie den Kurs aus, der Ihnen mehr zusagt. Bitte wählen Sie immer einen Kurs.

L5. Auswahlmöglichkeit	Kurs A	Kurs B
**Kurszeiten**	Nachmittags (16-18 Uhr)	Abends (18-20 Uhr)
**Dauer Geh-/Fahrweg bis zum BIG-Kurs**	Maximal 10 Minuten	Maximal 30 Minuten
**Zusätzliche soziale Aktivitäten im Rahmen von BIG organisiert**	Keine zusätzliche Aktivität	Zusätzliche soziale Aktivitäten
**Berücksichtigung von Interessen und Wünsche bei der weiteren Kursplanung**	Interessen/Wünsche werden berücksichtigt	Interessen/Wünsche werden nicht berücksichtigt
**Kurskosten (pro Termin)**	3,50 €	8,00 €
**Welchen Kurs bevorzugen Sie? (Kreuzen Sie bitte das entsprechende Kästchen an)**	☐	☐

Bitte wählen Sie den Kurs aus, der Ihnen mehr zusagt. Bitte wählen Sie immer einen Kurs.

L6. Auswahlmöglichkeit	Kurs A	Kurs B
**Kurszeiten**	Nachmittags (16-18 Uhr)	Morgens (8-10 Uhr)
**Dauer Geh-/Fahrweg bis zum BIG-Kurs**	Maximal 30 Minuten	Maximal 10 Minuten
**Zusätzliche soziale Aktivitäten im Rahmen von BIG organisiert**	Zusätzliche soziale Aktivitäten	Keine zusätzliche Aktivität
**Berücksichtigung von Interessen und Wünsche bei der weiteren Kursplanung**	Interessen/Wünsche werden berücksichtigt	Interessen/Wünsche werden nicht berücksichtigt
**Kurskosten (pro Termin)**	2,00 €	9,50 €
**Welchen Kurs bevorzugen Sie? (Kreuzen Sie bitte das entsprechende Kästchen an)**	☐	☐

Bitte wählen Sie den Kurs aus, der Ihnen mehr zusagt. Bitte wählen Sie immer einen Kurs.

For peer review only - http://bmjopen.bmj.com/site/about/guidelines.xhtml

L7. Auswahlmöglichkeit	Kurs A	Kurs B
**Kurszeiten**	Morgens (8-10 Uhr)	Nachmittags (16-18 Uhr)
**Dauer Geh-/Fahrweg bis zum BIG-Kurs**	Maximal 20 Minuten	Maximal 30 Minuten
**Zusätzliche soziale Aktivitäten im Rahmen von BIG organisiert**	Zusätzliche soziale Aktivitäten	Keine zusätzliche Aktivität
**Berücksichtigung von Interessen und Wünsche bei der weiteren Kursplanung**	Interessen/Wünsche werden berücksichtigt	Interessen/Wünsche werden nicht berücksichtigt
**Kurskosten (pro Termin)**	2,00 €	6,50 €
**Welchen Kurs bevorzugen Sie? (Kreuzen Sie bitte das entsprechende Kästchen an)**	☐	☐

Bitte wählen Sie den Kurs aus, der Ihnen mehr zusagt. Bitte wählen Sie immer einen Kurs.

L8. Auswahlmöglichkeit	Kurs A	Kurs B
**Kurszeiten**	Vormittags (10-12 Uhr)	Morgens (8-10 Uhr)
**Dauer Geh-/Fahrweg bis zum BIG-Kurs**	Maximal 10 Minuten	Maximal 30 Minuten
**Zusätzliche soziale Aktivitäten im Rahmen von BIG organisiert**	Keine zusätzliche Aktivität	Keine zusätzliche Aktivität
**Berücksichtigung von Interessen und Wünsche bei der weiteren Kursplanung**	Interessen/Wünsche werden nicht berücksichtigt	Interesse/Wünsche werden berücksichtigt
**Kurskosten (pro Termin)**	5,00 €	6,50 €
**Welchen Kurs bevorzugen Sie? (Kreuzen Sie bitte das entsprechende Kästchen an)**	☐	☐

Bitte wählen Sie den Kurs aus, der Ihnen mehr zusagt. Bitte wählen Sie immer einen Kurs.

L9. Auswahlmöglichkeit	Kurs A	Kurs B
**Kurszeiten**	Vormittags (10-12 Uhr)	Abends (18-20 Uhr)
**Dauer Geh-/Fahrweg bis zum BIG-Kurs**	Maximal 30 Minuten	Maximal 10 Minuten
**Zusätzliche soziale Aktivitäten im Rahmen von BIG organisiert**	Zusätzliche soziale Aktivitäten	Zusätzliche soziale Aktivitäten
**Berücksichtigung von Interessen und Wünsche bei der weiteren Kursplanung**	Interessen/Wünsche werden nicht berücksichtigt	Interessen/Wünsche werden berücksichtigt
**Kurskosten (pro Termin)**	2,00 €	8,00 €
**Welchen Kurs bevorzugen Sie? (Kreuzen Sie bitte das entsprechende Kästchen an)**	☐	☐

For peer review only - http://bmjopen.bmj.com/site/about/guidelines.xhtml

References

Aboagye, E. (2017). Valuing Individuals' Preferences and Health Choices of Physical Exercise. *Pain Ther*, 6(1), 85-91. https://doi.org/10.1007/s40122-017-0067-4

Aboagye, E., Hagberg, J., Axén, I., Kwak, L., Lohela-Karlsson, M., Skillgate, E., Dahlgren, G., & Jensen, I. (2017). Individual preferences for physical exercise as secondary prevention for non-specific low back pain: A discrete choice experiment. *PLoS One*, 12(12), e0187709. https://doi.org/10.1371/journal.pone.0187709

Alayli-Goebbels, A. F., Dellaert, B. G., Knox, S. A., Ament, A. J., Lakerveld, J., Bot, S. D., Nijpels, G., & Severens, J. L. (2013). Consumer preferences for health and nonhealth outcomes of health promotion: results from a discrete choice experiment. *Value Health*, 16(1), 114-123. https://doi.org/10.1016/j.jval.2012.08.2211

Benning, T. M., Dellaert, B. G. C., & Arentze, T. A. (2020). The impact of health vs. non-health goals on individuals' lifestyle program choices: a discrete choice experiment approach. *BMC Public Health*, 20(1), 411. https://doi.org/10.1186/s12889-020-8416-3

Brown, D. S., Finkelstein, E. A., Brown, D. R., Buchner, D. M., & Johnson, F. R. (2009). Estimating older adults' preferences for walking programs via conjoint analysis. *Am J Prev Med*, 36(3), 201-207.e204. https://doi.org/10.1016/j.amepre.2008.10.014

Dintsios, C. M., Chernyak, N., Grehl, B., & Icks, A. (2018). Quantified patient preferences for lifestyle intervention programs for diabetes prevention-a protocol for a systematic review. *Syst Rev*, 7(1), 214. https://doi.org/10.1186/s13643-018-0884-5

Farooqui, M. A., Tan, Y. T., Bilger, M., & Finkelstein, E. A. (2014). Effects of financial incentives on motivating physical activity among older adults: results from a discrete choice experiment. *BMC Public Health*, 14, 141. https://doi.org/10.1186/1471-2458-14-141

Ferreira, G. E., Howard, K., Zadro, J. R., O'Keeffe, M., Lin, C. C., & Maher, C. G. (2020). People considering exercise to prevent low back pain recurrence prefer exercise programs that differ from programs known to be effective: a discrete choice experiment. *J Physiother*, 66(4), 249-255. https://doi.org/10.1016/j.jphys.2020.09.011

Geidl, W., Knocke, K., Schupp, W., & Pfeifer, K. (2018). Measuring stroke patients' exercise preferences using a discrete choice experiment. *Neurol Int*, 10(1), 6993. https://doi.org/10.4081/ni.2018.6993

Giles, E. L., Becker, F., Ternent, L., Sniehotta, F. F., McColl, E., & Adams, J. (2016). Acceptability of Financial Incentives for Health Behaviours: A Discrete Choice Experiment. *PLoS One*, 11(6), e0157403. https://doi.org/10.1371/journal.pone.0157403

Kjaer, T., Gyrd-Hansen, D., & Willaing, I. (2006). Investigating patients' preferences for cardiac rehabilitation in Denmark. *Int J Technol Assess Health Care*, 22(2), 211-218. https://doi.org/10.1017/s0266462306051038

Matsushita, M., Harada, K., & Arao, T. (2017). [Incentive program to strengthen motivation for increasing physical activity via conjoint analysis]. *Nihon Koshu Eisei Zasshi*, 64(4), 197-206. https://doi.org/10.11236/jph.64.4_197

Molema, C., Veldwijk, J., Wendel-Vos, W., de Wit, A., van de Goor, I., & Schuit, J. (2019). Chronically ill patients' preferences for a financial incentive in a lifestyle intervention. Results of a discrete choice experiment. *PLoS One*, 14(7), e0219112. https://doi.org/10.1371/journal.pone.0219112

For peer review only - http://bmjopen.bmj.com/site/about/guidelines.xhtml

Owen, K., Pettman, T., Haas, M., Viney, R., & Misan, G. (2010). Individual preferences for diet and exercise programmes: changes over a lifestyle intervention and their link with outcomes. *Public Health Nutr*, 13(2), 245-252. https://doi.org/10.1017/s1368980009990784

Paul, S. S., Canning, C. G., Löfgren, N., Sherrington, C., Lee, D. C., Bampton, J., & Howard, K. (2021). People with Parkinson's disease are more willing to do additional exercise if the exercise program has specific attributes: a discrete choice experiment. *J Physiother*, 67(1), 49-55. https://doi.org/10.1016/j.jphys.2020.12.007

Pinto, D., Bockenholt, U., Lee, J., Chang, R. W., Sharma, L., Finn, D. J., Heinemann, A. W., Holl, J. L., & Hansen, P. (2019). Preferences for physical activity: a conjoint analysis involving people with chronic knee pain. *Osteoarthritis Cartilage*, 27(2), 240-247. https://doi.org/10.1016/j.joca.2018.10.002

Pinto, D., Danilovich, M. K., Hansen, P., Finn, D. J., Chang, R. W., Holl, J. L., Heinemann, A. W., & Bockenholt, U. (2017). Qualitative Development of a Discrete Choice Experiment for Physical Activity Interventions to Improve Knee Osteoarthritis. *Arch Phys Med Rehabil*, 98(6), 1210-1216.e1211. https://doi.org/10.1016/j.apmr.2016.11.024

Ramirez, M., Wu, S., & Beale, E. (2016). Designing a Text Messaging Intervention to Improve Physical Activity Behavior Among Low-Income Latino Patients With Diabetes: A Discrete-Choice Experiment, Los Angeles, 2014-2015. *Prev Chronic Dis*, 13, E171. https://doi.org/10.5888/pcd13.160035

Robles, L. A., Wright, S. J., Hackshaw-McGeagh, L., Shingler, E., Shiridzinomwa, C., Lane, J. A., Martin, R. M., & Burden, S. (2020). Prostate cancer survivors' preferences on the delivery of diet and lifestyle advice: a pilot best-worst discrete choice experiment. *Pilot Feasibility Stud*, 6, 2. https://doi.org/10.1186/s40814-019-0549-8

Ryan, M., Yi, D., Avenell, A., Douglas, F., Aucott, L., van Teijlingen, E., & Vale, L. (2015). Gaining pounds by losing pounds: preferences for lifestyle interventions to reduce obesity. *Health Econ Policy Law*, 10(2), 161-182. https://doi.org/10.1017/s1744133114000413

Salampessy, B. H., Veldwijk, J., Jantine Schuit, A., van den Brekel-Dijkstra, K., Neslo, R. E., Ardine de Wit, G., & Lambooij, M. S. (2015). The Predictive Value of Discrete Choice Experiments in Public Health: An Exploratory Application. *Patient*, 8(6), 521-529. https://doi.org/10.1007/s40271-015-0115-2

Sommer, J., Dyczmons, J., Grobosch, S., Gontscharuk, V., Vomhof, M., Roden, M., & Icks, A. (2020). Preferences of people with type 2 diabetes for telemedical lifestyle programmes in Germany: protocol of a discrete choice experiment. *BMJ Open*, 10(9), e036995. https://doi.org/10.1136/bmjopen-2020-036995

van Gils, P. F., Lambooij, M. S., Flanderijn, M. H., van den Berg, M., de Wit, G. A., Schuit, A. J., Struijs, J. N., & van den Berg, B. (2011). Willingness to participate in a lifestyle intervention program of patients with type 2 diabetes mellitus: a conjoint analysis. *Patient Prefer Adherence*, 5, 537-546. https://doi.org/10.2147/ppa.S16854

Veldwijk, J., Lambooij, M. S., de Bekker-Grob, E. W., Smit, H. A., & de Wit, G. A. (2014). The effect of including an opt-out option in discrete choice experiments. *PLoS One*, 9(11), e111805. https://doi.org/10.1371/journal.pone.0111805

Veldwijk, J., Lambooij, M. S., van Gils, P. F., Struijs, J. N., Smit, H. A., & de Wit, G. A. (2013). Type 2 diabetes patients' preferences and willingness to pay for lifestyle programs: a discrete choice experiment. *BMC Public Health*, 13, 1099. https://doi.org/10.1186/1471-2458-13-1099

For peer review only - http://bmjopen.bmj.com/site/about/guidelines.xhtml

Wanders, J. O., Veldwijk, J., de Wit, G. A., Hart, H. E., van Gils, P. F., & Lambooij, M. S. (2014). The effect of out-of-pocket costs and financial rewards in a discrete choice experiment: an application to lifestyle programs. *BMC Public Health*, 14, 870. https://doi.org/10.1186/1471-2458-14-870

For peer review only

For peer review only - http://bmjopen.bmj.com/site/about/guidelines.xhtml
